# Exposing Mechanisms for Defect Clearance in Supramolecular Self-Assembly: Palladium–Pyridine Coordination Revisited

**DOI:** 10.1021/acs.inorgchem.2c04404

**Published:** 2023-03-24

**Authors:** David A. Poole, Eduard O. Bobylev, Bas de Bruin, Simon Mathew, Joost N. H. Reek

**Affiliations:** Homogeneous, Supramolecular, and Bioinspired Catalysis group, van’t Hoff Institute for Molecular Science (HIMS), University of Amsterdam (UvA), Science Park 904, 1098 XH Amsterdam, The Netherlands

## Abstract

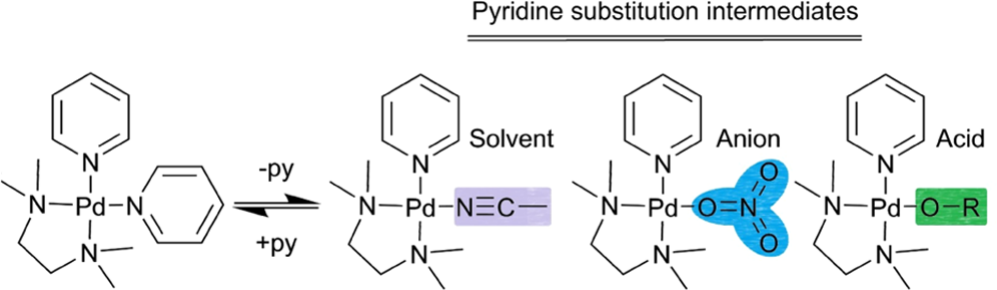

Spherical three-dimensional (3D) cages composed of palladium(II) and pyridyl ligands are a mainstay of supramolecular chemistry with demonstrated catalytic and optoelectronic applications. The widely reported self-assembly of these palladium-based cages exhibits sensitivity to the solvents, reagents, and/or reactants employed. This sensitivity, and the resulting inconsistency between synthetic protocols, hinders the development of desirable palladium-based cages. We have found that pyridyl ligand substitution—the rate-limiting step of self-assembly—is facilitated by endogenous supporting ligands derived from the solvents, reagents, and reactants employed in synthetic protocols of palladium- and platinum-based assemblies. Here, we present a systematic investigation combining ^1^H-NMR, electrospray ionization mass spectrometry (ESI—MS), and absorption spectroscopy to characterize the intermediates to support the mechanism of pyridyl ligand substitution on a model complex, **M**(**py**)_2_ (**M** = (*N,N,N*′,*N*′-tetramethylethylenediamine)palladium(II), **py** = pyridine), under simulated synthetic conditions for self-assembly. Our investigation exposes mechanisms for pyridyl ligand substitution, featuring intermediates stabilized by solvent, anion, or (*in situ* formed) alkoxide moieties. Interrogation of destabilizing agents (2,2,2-trifluoroethanol and tetra(*n*-butyl)ammonium chloride) reveal similar mechanisms that ultimately facilitate the self-assembly of coordination cages. These findings rationalize widely reported solvent and anion effects in the self-assembly of coordination cages (and similar constructs) while highlighting methodologies to understand the role of supporting ligands in coordination chemistry.

## Abstract 2

Self-assembled supramolecular cages derived from palladium(II) and pyridyl ligands are widely used in supramolecular chemistry, but the synthetic protocols exhibit sensitivity to solvents, reagents, and reactants, leading to inconsistencies. We present a systematic investigation using 1H-NMR, ESI-MS, and absorption spectroscopy to characterize intermediates and support the mechanism of pyridyl ligand substitution. Our findings rationalize solvent and anion effects and provides a basis for controlling the self-assembly of supramolecular cages under a variety of reaction conditions.

## Introduction

**Scheme 1:**
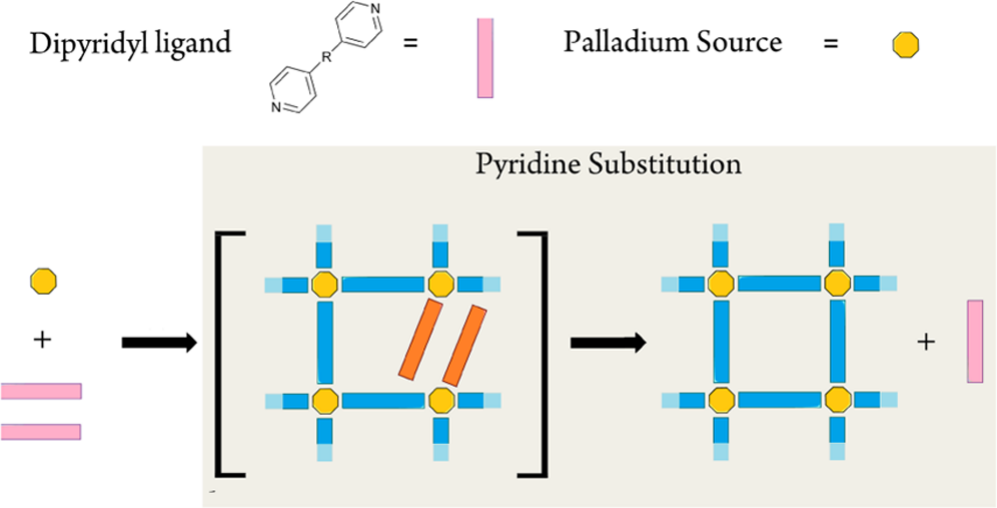
Defect Clearance from Pd^2+^ Frameworks

Discrete supramolecular three-dimensional (3D) cages featuring palladium (II) coordination nodes have garnered significant interest in contemporary chemistry^1−18^ due to their novel optoelectronic^19−28^ and catalytic applications.^29−42^ These Pd-based cages are synthesized by a self-assembly process that exploits the reversibility of coordination bonds between ditopic pyridyl ligands and Pd-containing coordination nodes to afford products bearing the minimum free energy (i.e., Δ*G*).^43−50^ The intermediates of this synthetic process include defective frameworks, wherein two ligands occupy a single ligand site during self-assembly (Scheme 1, orange).^51−60^ Importantly, the process of defect clearance via substitution of a coordinated ditopic ligand (i.e., pyridyl ligand substitution) is the rate-limiting step in the self-assembly of coordination cages.^51^

The literature proposes an associative mechanism for pyridyl ligand substitution, where an incoming ligand coordinates to an existing (four-coordinate) Pd complex. The result is the formation of a five-coordinate transition state, which ejects a ligand to yield a four-coordinate complex.^51^ This mechanism is supported by theoretical studies,^61,62^ but does not account for the significant steric hindrance incurred that can inhibit defect clearance within the framework (Scheme 1, orange).^63−66^ The nature of this steric encumbrance is exacerbated by the geometric constraints imposed on pyridyl ligands that are incorporated into the surrounding rigid framework (Scheme 1, blue).^67^

Reported self-assembly protocols for supramolecular cages utilize a diversity of Pd^2+^ salt precursors and reaction solvents, highlighting the significance of judicious pairing of reagent/reactant/solvent combinations in achieving desirable products with good synthetic yields.^68^ While many studies have detailed the effect of ditopic ligand design on the topology of product cages,^1−16,63−70^ few have considered the effect of specific solvents, reagents, or reactants (i.e., Pd^2+^ precursor). Hiraoka et al. reported that the use of coordinating solvents (specifically, MeCN) and elevated temperatures (343.15 K) improved the yield of cuboctahedral Pd_12_L_24_ coordination cages by facilitating pyridyl ligand substitution and promoting defect clearance.^71^ Fujita et al. demonstrated that 2,2,2-trifluoroethanol (TFE) as a co-solvent (80% v/v in DMSO) improved the formation of similar Pt_12_L_24_ cuboctahedral cages based on platinum (II) metal centers, acting through the destabilization of coordination bonds.^72^ Recently, we reported that trace quantities of chloride (Cl^–^) or the addition of carboxylic acids facilitate the self-assembly of both Pd_12_L_24_ and Pt_12_L_24_.^69,73^ We propose that these additive reagents catalyze the supramolecular self-assembly of Pd_*n*_L_2*n*_ cages through a common mechanism, namely, by facilitating the elementary step of pyridyl ligand substitution at Pd coordination nodes. Thus, a realization of reactant or reagent effects on the thermodynamics of pyridyl ligand substitution should generate a rationale that improves the synthesis of many Pd-based coordination cages.

In this work, we engage in a systematic investigation of pyridyl ligand substitution of a model complex **M**(**py**)_2_ (**M** = (*N,N,N*′,*N*′-tetramethylethylenediamine)palladium(II), **py** = pyridine). By employing variable-temperature (VT) ^1^H-NMR and 1D-exchange spectroscopy (EXSY), we determine the rates and activation energies (*E*_*a*_) for pyridyl ligand substitution under different reagent and reactant conditions. Specifically, we demonstrate that solvents, counterions, and additives (Cl^–^ and TFE) enable different mechanisms of pyridyl ligand substitution facilitated by endogenous supporting ligands. These mechanisms are further evidenced by electrospray ionization mass spectrometry (ESI—MS), computational, and VT absorption spectroscopy studies of the intermediate **M**(**py**)(**L**) complexes (where **L** is the endogenous supporting ligand, e.g., MeCN). We infer that analogous pyridyl ligand substitution mechanisms likely occur during the self-assembly of Pd-based coordination cages. This leads to improved synthetic yields observed in previous reports,^71−73^ as rate-limiting defect clearance within the framework is highly favored.^51−60^ Importantly, this mechanism relies upon small-molecular entities as supporting ligands, circumventing the steric hindrance evoked by the presence of axially-coordinated pyridyl ligands.^61−63^

## Results

### ^1^H-NMR Analysis of Mono- and Bis-Pyridyl Complexes in Noncoordinating Solvents

**Figure 1:**
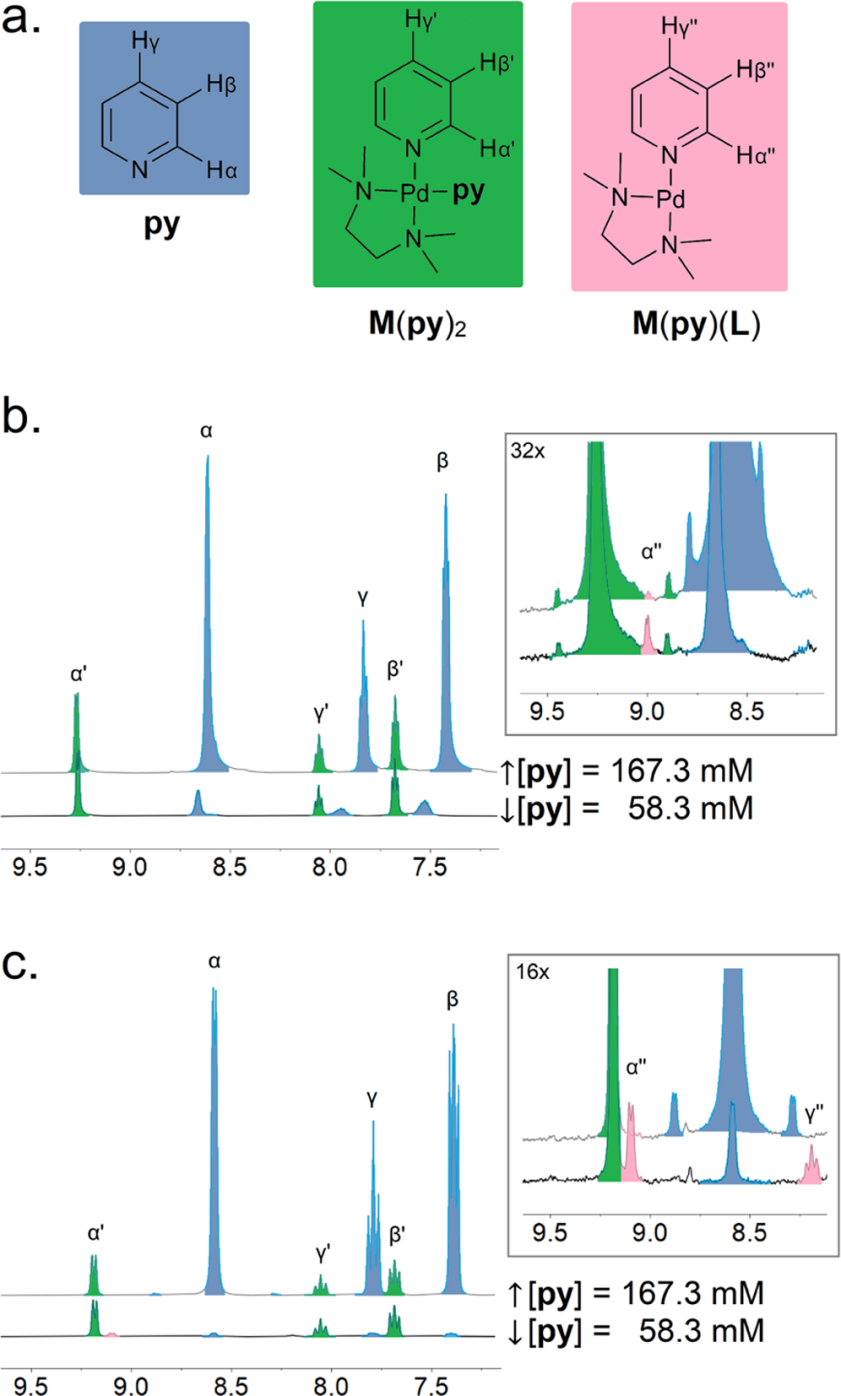
(a) Structure of pyridyl species **py**, **M**(**py**)_2_, and **M**(**py**)(**L**) occurring in our model system for pyridyl ligand substitution. ^1^H-NMR spectra of pyridyl species in both (b) MeNO_2_ and (c) DMSO. Different **py** concentrations ([**py**], listed alongside each trace) were used to affect the presence of **M**(**py**)(**L**) complexes (inset, pink). See Figures S2–S5 for spectra and acquisition details.

The literature proposes that pyridyl ligand substitution is limited by the slow, axial association of an incoming **py** ligand or coordinating solvent molecule.^51,61,62^ Therefore, we created a model system of **M** ([**M**] = 17.3 mM) and **py** at low ([**py**] = 58.3 mM) or high ([**py**] = 167.3 mM) concentrations, in either nitromethane-*d*_3_ (MeNO_2_) or dimethylsulfoxide-*d*_6_ (DMSO). These samples were analyzed by ^1^H-NMR to observe the speciation of mono-pyridyl **M**(**py**)(**L**) (where **L** is a supporting ligand such as a solvent or anion) and bis-pyridyl **M**(**py**)_2_ complexes (Figure 1).

In addition to **M**(**py**)_2_ and free **py** (Figure 1, resonances α′ and α, respectively), a second complex is observed (Figure 1, resonance α″) that was identified as **M**(**py**)(**L**) (where **L** is a supporting ligand) by diffusion-ordered spectroscopy in DMSO (DOSY, Figure S1). This species is observed only in samples containing a lower excess of **py** ([**py**] = 58.3 mM). The formation of **M**(**py**)(**L**) in the presence of an excess of **py** indicates that entropic penalties of forming **M**(**py**)_2_ are not countered by the enthalpic gains from bond formation between Pd^2+^ and **py**.^74^ In contrast, a greater excess of **py** ([**py**] = 167.3 mM) results in the quantitative formation of **M**(**py**)_2_.

While the literature proposes that the association of free **py** limits pyridyl ligand substitution in Pd-based complexes,^51^ we observe the formation of **M**(**py**)(**L**) is suggestive of a supporting ligand-dependent or dissociative mechanism (Figure 1b). These results are supported by CSI—HRMS measurements (Figure S6), and ^1^H-NMR spectra (Figure S39) that indicate the presence of **M**(**py**)(NO_3_^–^) (i.e., **L** = NO_3_^–^) under these conditions. Therefore, we propose that NO_3_^–^ acts as an endogenous ligand to encourage the formation of **M**(**py**)(**L**) complexes that are amenable to further substitution.

### EXSY Analysis of py Substitution in M(py)_2_ Complexes in Coordinating and Noncoordinating Solvents

**Figure 2:**
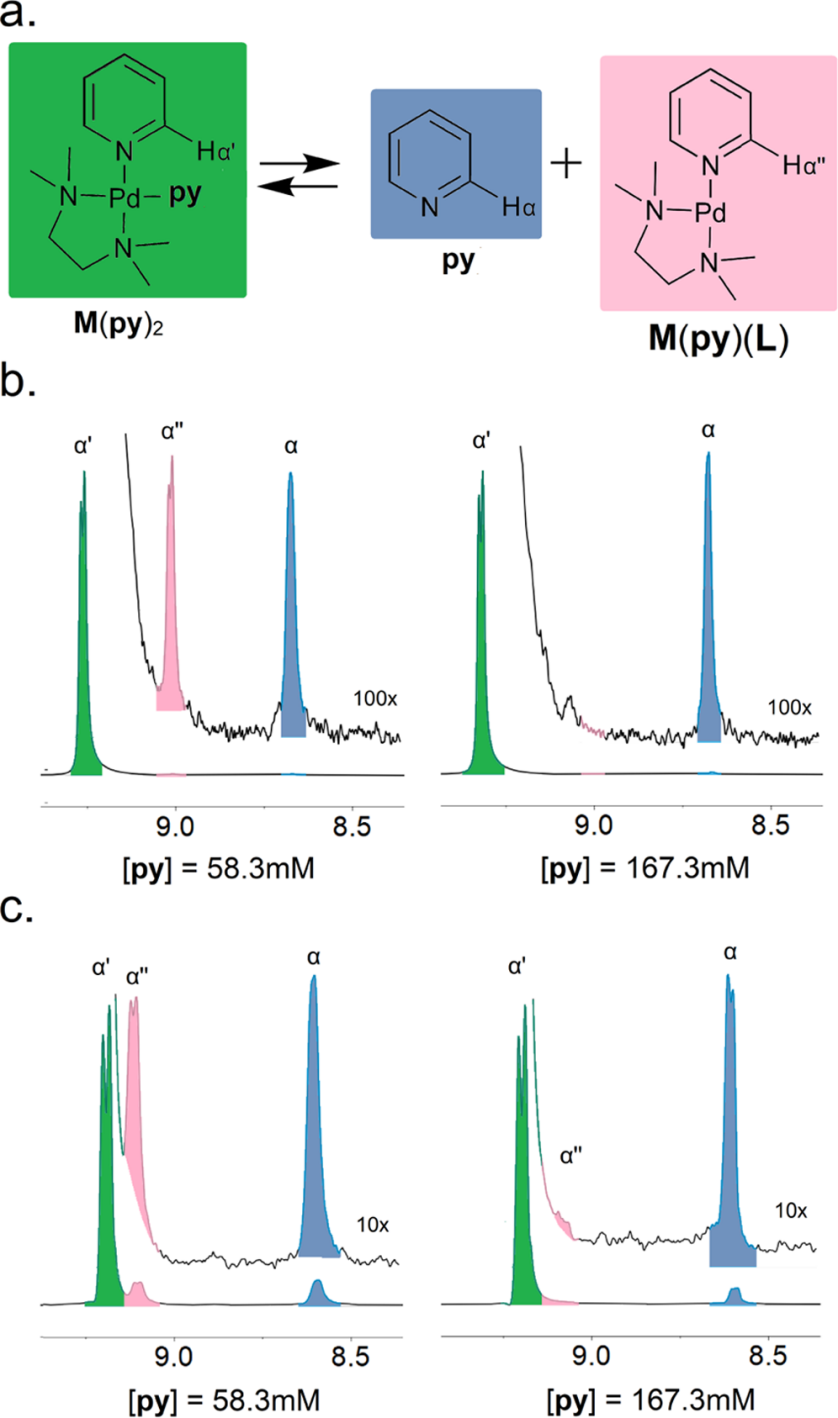
(a) Dissociation **M**(**py**)_2_ complexes observed with EXSY. Quantification of **py** dissociation of **M**(**py**)_2_ using EXSY to observe product peaks (α, α″) upon excitation of α′ in (b) MeNO_2_ or (c) DMSO; see Figures S8–S11 for full EXSY spectra.

The dissociation of **M**(**py**)_2_ affords an **M**(**py**)(**L**) complex (where L is a supporting ligand) and free py (eq 1).
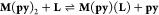
1With one-dimensional (1D) gradient-edited EXSY, the excitation of the α-proton of the **py** ligand within **M**(**py**)_2_ (Table 1, α′) was used to quantify the evolution of peaks for the products of **py** substitution (Figure 2). This approach is sensitive to the peaks arising from chemical exchange allowing for direct measurement of rate.^75^

**Table 1:** Select α-Pyridyl Proton Peaks for the Model System

peak	complex	δ_MeNO2_ (ppm)	δ_DMSO_ (ppm)
α	**py**	8.66	8.58
α′	**M**(**py**)_2_	9.26	9.18
α″	**M**(**py**)(**L**)	8.99^a^	9.10^a^

a Not observed by ^1^H-NMR when [**py**] = 167.3 mM.

The excitation of α-protons in coordinated **py** (Figure 2, green, α′) leads to the evolution of peaks corresponding to free py (Figure 2, blue, α) and **M**(**py**)(**L**) (Figure 2, pink, α″). Using a standard equation (eq 2),^75^ we computed the rate constant (*k*, s^–1^) of chemical exchange or reaction from the integrated areas of the excited peak (α′ as *I*_excited_) and an evolved peak (α or α″ as *I*_evolved_) arising over the mixing time of the EXSY experiment (*T*_m_, s).
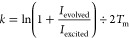
2From the data in Figure 2, we computed the rate constants for the production of free **py** (*k*_**py**_) and **M**(**py**)(**L**) (*k*_**M**(**py**)(**L**)_) afforded by the dissociation of **M**(**py**)_2_ complexes (Table 2). When our model complex is formed with a lower excess of **py** ([**py**] = 58.3 mM), *k*_**py**_ and *k*_**M**(**py**)(**L**)_ are identical, which is expected as these form from the same complex (Table 2, entries 1 and 2). In contrast, elevated concentrations of **py** ([**py**] = 167.3 mM) show that *k*_**M**(**py**)(**L**)_ is significantly diminished compared to *k*_**py**_ (Table 2, entries 1–2). This result parallels our ^1^H-NMR observations (Figure 1), where α″ diminished at the same concentration of **py** ([**py**] = 167.3 mM). The disappearance of α″ suggests that **M**(**py**)(**L**) is being converted to **M**(**py**)_2_ by the association of **py** faster than the timescale of the ^1^H-NMR measurement when [**py**] = 167.3 mM.^86^ A rapid association suggests that pyridyl ligand substitution is limited by the dissociation of the pyridyl ligand, which we observe directly as *k*_py_. This **py**-dissociation-limited mechanism is further supported by the invariance of *k***_py_** to changes in **py** concentration in both coordinating (DMSO, *k*_**py**_ = 0.035 s^–1^) and noncoordinating (MeNO_2_, *k*_**py**_ = 0.011 s^–1^) solvents. However, the difference in these rates points to the role of endogenous ligands (i.e., **L**) and the importance of solvent coordination (**L** = DMSO) in the pyridyl ligand substitution process.

**Table 2:** Measured Rate Constants of py Substitution at 300 K

entry	solvent	[**py**] (mM)	*k*_**py**_ (α, s^–1^)	*k*_**M**(**py**)(**L**)_ (α″, s^–1^)
1	DMSO	58.3	0.035	0.035
2	MeNO_2_	58.3	0.012	0.011
2	DMSO	167.3	0.035	0.004^a^
4	MeNO_2_	167.3	0.011	0.000^a^

a Poorly resolved peak (Figure 2, pink).

### Thermochemical Analysis of Pyridyl Ligand Substitution with M(py)_2_ Complexes in the Presence of Different Anions

**Figure 3:**
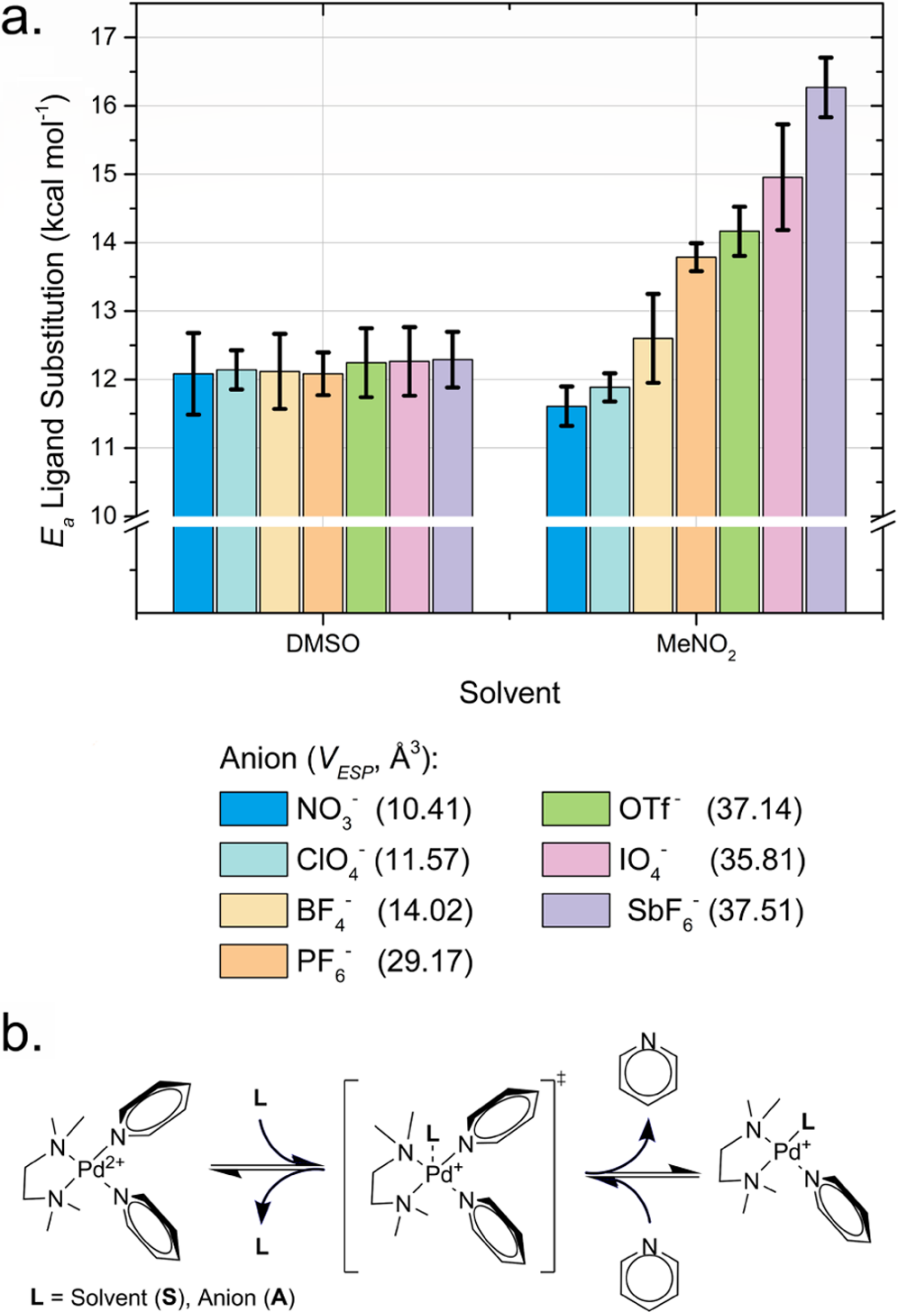
(a) Activation energies (*E*_a_) for pyridyl ligand substitution of **M**(**py**)_2_ complexes formed from **py** ([**py**] = 167.3 mM) and **M** ([**M**] = 17.3 mM), where **M** was prepared with a variety of anions (Scheme S1). (b) General mechanism proposed for pyridyl ligand substitution of **M**(**py**)_2_ facilitated by endogenous ligands (**L**), such as neutral solvent molecules (**L** = **S**) or anions (**L** = **A**). These *E*_a_ values were computed from the EXSY-determined rates across a range of temperatures (300–355 K, Figures S13–S26) by a direct fit of eq 3 (Figure S33a,b). Results are additionally provided numerically in Table S1. The electrostatic volumes (*V*_esp_, Å^3^) of anions were determined by fitting DFT-computed electron densities (Figure S35).

The use of VT–EXSY allows the measurement of pyridyl ligand substitution rates across a range of temperatures. Fitting these rate data with the Arrhenius equation (eq 3) affords both activation energy (*E*_a_), as a characteristic thermodynamic barrier for pyridyl ligand substitution at **M**(**py**)_2_, and a preexponential factor (*A*) representing environmental contributions.
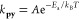
3Crucially, *E*_a_ reflects the transition state enthalpy of the rate-limiting step of the specific reaction mechanism, which is independent of concentration or entropic contributions. Therefore, these measured *E*_a_ values reflect both the mechanism and facility of pyridyl ligand substitution under different reactant and reagent conditions. To distinguish differences in pyridyl ligand substitution from solvent or anion selection, we determined the *E*_a_ of **py** dissociation in **M**(**py**)_2_ complexes in both MeNO_2_ and DMSO solvents, where the initial complexes were prepared with a range of anions (NO_3_^–^, ClO_4_^–^, BF_4_^–^, PF_6_^–^, OTf^–^, IO_4_^–^, and SbF_6_^–^) present as part of the metal precursor employed (Figure 3).

The measured *E*_a_ values for **py** substitution in DMSO indicate that the transition state of the rate-limiting step (see below) is invariant to the counterion of the **M**(**py**)_2_ complex (Figure 3a, left columns). This is consistent with the association of DMSO—a coordinating solvent—to form an intermediate **M**(**py**)(DMSO) complex. Prior computational studies demonstrated that the coordination of an acetonitrile molecule (MeCN) to a Pd^2+^ center may displace a bound **py** via a five-coordinate transition state (Figure 3b, **L** = MeCN).^51^ We propose that this mechanism of solvent-facilitated pyridyl ligand substitution occurs with all coordinating solvents (Figure 3b, **L** = **S**, generally), leading to their favorable use in self-assembly protocols.^68^

When a noncoordinating solvent (i.e., MeNO_2_) is used, the *E*_a_ of pyridyl ligand substitution of **M**(**py**)_2_ complexes becomes sensitive (i.e., dependent) on the anion used (Figure 3, right columns). As *E*_a_ varies only with the relative enthalpy of the five-coordinate transition state, these anion effects do not originate from the solubility of the anion. This leads us to surmise that each anion affords a unique intermediate complex, **M**(**py**)(**A**), via a five-coordinate transition state followed by ejection of a **py** ligand (Figure 3b, **L** = **A**).

The formation and subsequent degradation of **M**(**py**)(**A**) results in the substitution of a **py** ligand at the **M**(**py**)_2_ center. Stabilization of the five-coordinate transition state, **M**(**py**)_2_(**A**), leads to a favorable *E*_a_ (i.e., minimum *E*_a_). We found that the anion electrostatic volume (*V*_esp_) correlated to the measured *E*_a_ values (Figure S36), indicating that the steric volume (or charge density) of an ion serves to limit its efficacy as a supporting ligand.

Interestingly, NO_3_^–^ is smaller than DMSO (*V*_esp_ = 10.41 vs 14.71 Å^3^) and stabilizes the transition state more effectively (*E*_a_ = 11.61 vs 12.08 kcal mol^–1^). Despite these physical and thermodynamic metrics, solvent-facilitated ligand substitution is preferred. Presumably, this preference is due to the sheer abundance of DMSO compared to NO_3_^–^ (14.1 M vs 34.6 mM, respectively, ca. 400 equiv), but this also does not account for any elevation in local concentrations that arise from ion pairing in solution. From the reports of Hiraoka and co-workers on transient supramolecular cage formation complexes (Scheme 1),^71^ we considered that solvation may determine the mechanism of pyridyl ligand substitution.

### MD Simulation of Solvation Effects on Anion-Pair Formation

**Figure 4:**
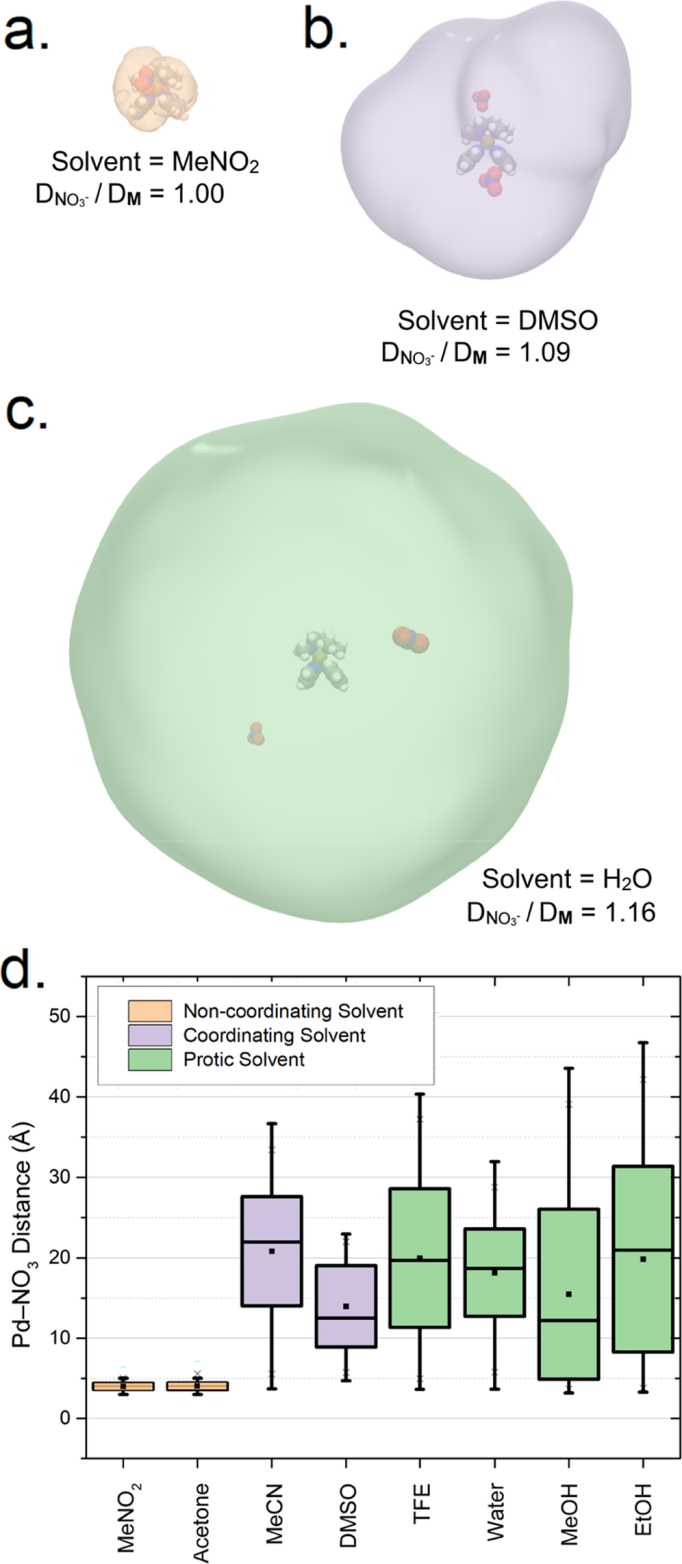
Renderings of the volume of space traversed by NO_3_^–^ anions over MD simulations (1 μs) with explicit solvent (a) MeNO_2_,^77^ (b) DMSO,^78^ and (c) OPC model water.^76^ (d) Box plot showing the distribution of Pd–N distances from the full range of solvents used in this study: the box represents the 25–75th percentiles with a line at the median, the whiskers include all values, the average is represented as black dot within the box. The renderings (a–c) are generated from identical virtual perspectives to enable visual comparison. All MD simulations featured at least 5000 explicit solvent molecules in a periodic box (cubic 2700 Å^3^) with **M**(**py**)_2_ neutralized by 2 × NO_3_^–^ anions. Parameters for **M**(**py**)_2_ complexes were developed by standard protocols (Figure S38), while solvent and ion models were adapted from the literature.^76−80^

The ion pairing that arises from the electrostatic attraction between **M**(**py**)_2_ and NO_3_^–^ may be mediated by intermediary polar-solvent molecules providing a basis for observed changes in ion interactions under different solvent conditions (Figure 3).^85^ Stronger ion pairing increases the local concentration of NO_3_^–^ near the **M**(**py**)_2_ complex and therefore elevates the collisional frequency of contact between them. Unfortunately, DOSY and ^1^H-NMR are not suited to directly observe ion pairing interactions between **M**(**py**)_2_ complexes and NO_3_^–^ in solution. While measurement with ^14^N-, or ^15^N-NMR may be possible, we could not obtain sufficiently well-resolved spectra at their respective frequencies with our instrumentation (data not provided). Instead, we developed MD simulations to observe how solvent interaction attenuates ion pairing (Scheme S2). From these MD trajectories (1 μs), we visualized the volumes traversed by NO_3_^–^ and quantified NO_3_^–^ ion pairing using computed diffusion- and distance-based metrics (Figure 4).

Our MD simulations directly show that ion pairing between NO_3_^–^ and **M**(**py**)_2_ is sensitive to solvent conditions, and the increasingly favorable solvation by DMSO/water enables NO_3_^–^ to traverse across larger volumes around the complex during the simulation (Figure 4b,c) compared to MeNO_2_ (Figure 4a). The mobility of NO_3_^–^ about the **M**(**py**)_2_ complex was measured as the quotient of the diffusion constants for the two species (*D*_NO_3__/*D*_***M***_, unitless),^85^ where a ratio of 1.00 observed for MeNO_2_ shows minimal translational freedom between the ions (Figure 4a). The NO_3_^–^ anions proved to be increasingly mobile in DMSO and water leading to diffusivity ratios of 1.09 and 1.16, consistent with solvent separation (Figure 4b,c).^85^

This effect is also visualized by the distribution of Pd–NO_3_^–^ distances (Figure 4d), demonstrating that coordinating or protic solvents, in general, diminish the frequency of contact between NO_3_^–^ and **M**. These results evidence that solvents drive mechanistic preferences by impairing anion-facilitated pyridyl ligand substitution. Since this effect may be independent of a solvent’s capacity for coordination, this effect may be exploited to control or halt self-assembly to isolate kinetically trapped assemblies.

### Thermochemical Investigation of Coordinating, Noncoordinating, and Protic Solvents on Pyridyl Ligand Substitution

**Figure 5:**
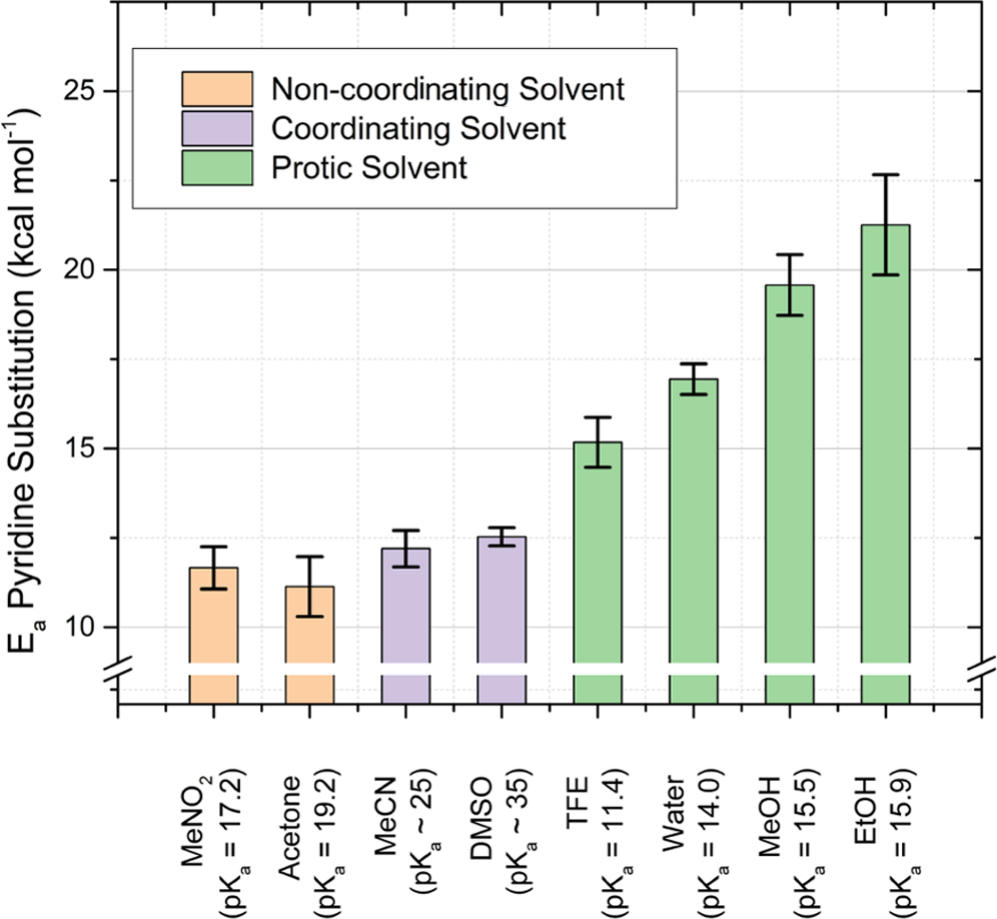
Plotted *E*_a_ values determined for pyridyl substitution of complexes formed from **M**(NO_3_^–^)_2_ (17.3 mM) and **py** (Figures S13b, S20b, S27b–S30b, S30c) in noncoordinating (orange), coordinating (purple), and protic solvents (green), shown with reference p*K*_a_ values.^82^ Activation energies were determined by the Arrhenius equation using EXSY-determined ligand substitution rates obtained over a range of temperatures appropriate for each solvent (i.e., from 300 K to within 5 K of solvent boiling point, see Figure S33c). These results are provided numerically in Table S2.

As a solvent or co-solvent, TFE is uniquely employed in the self-assembly of Pd_*n*_L_2*n*_ cages to improve synthetic yields.^72^ However, the effect of other protic solvents (e.g., water, MeOH, EtOH) on self-assembly or pyridyl ligand substitution is unclear. As protic solvents are weakly coordinating^81^ and readily solvate NO_3_^–^ (Figure 4c,d), it is uncertain if pyridyl ligand substitution is facilitated by endogenous ligands under these conditions or unaffected by solvent or anion choices via a dissociation-limited mechanism. Therefore, we determined the characteristic *E*_a_ of pyridine dissociation using our VT–NMR approach to gain insight into the role of solvent proticity in ligand substitution (Figure 5).

Our VT–NMR measurements reveal that aprotic solvents have a lower *E*_a_ value (11.1–12.5 kcal mol^–1^) for pyridyl ligand substitution compared to the studied protic solvents (15.2–21.3 kcal mol^–1^). The lower *E*_a_ of aprotic solvents suggests improved defect clearance and is consistent with their frequent use in synthetic protocols.^68^ Both of the noncoordinating aprotic solvents examined (MeNO_2_ and acetone) have a similar *E*_a_, (11.6 ± 0.6 and 11.1 ± 0.8 kcal mol^–1^, respectively), suggestive of a NO_3_^–^-facilitated mechanism (Figure 3b, **L** = NO_3_^–^). Interestingly, when a coordinating solvent facilitates pyridine exchange (i.e., with MeCN, and DMSO), the *E*_a_ values for pyridyl ligand substitution (12.2 ± 0.5 and 12.1 ± 0.6 kcal mol^–1^, respectively) have different coordination strengths.^81^ We surmise that this discrepancy may arise from weaker O^–^ coordination between the Pd^2+^ of **M**(**py**)_2_ and DMSO,^83^ promoted by steric hindrance within the **M**(**py**)_2_(DMSO) and its larger *V*_esp_ (Figure S35h, *V*_esp_ = 14.7 Å^3^).

Contrasting behavior was observed for pyridyl ligand substitution in protic media, with large differences in the *E*_a_ apparent that correlated to the respective proton association (i.e., p*K*_a_) of the solvent. Previous reports propose that TFE acts as an acid, protonating the **M**-coordinated **py** and disrupting the **py**-Pd^2+^ to yield **M**(**py**)(**L**) and **py**H^+^.^72^ However, quantum-chemical modeling of the H^+^ association to the bound **py** with GFN2-xTB^87^ suggests that electrostatic repulsion between the Pd^2+^ center of **M**(**py**)_2_ and H^+^ occurs with a higher energy barrier than the direct dissociation of **py** (Figure S37). Therefore, we propose that the dissociation of a proton from solvent affords an alkoxide (i.e., RO^–^) that acts as the anionic supporting ligand to facilitate pyridyl ligand substitution (Figure 3b, **L** = RO^–^). This proposed mode of action rationalizes the use of TFE (p*K*_a_ = 11.4) to promote the formation of large coordination cages by providing a reservoir of 2,2,2-trifluoroethanolate (**TFEO**^**–**^) as supporting ligands for pyridyl ligand substitution.^72^

### Characterization of Intermediate M(py)(L) Complexes Using VT Absorption Spectroscopy

**Figure 6:**
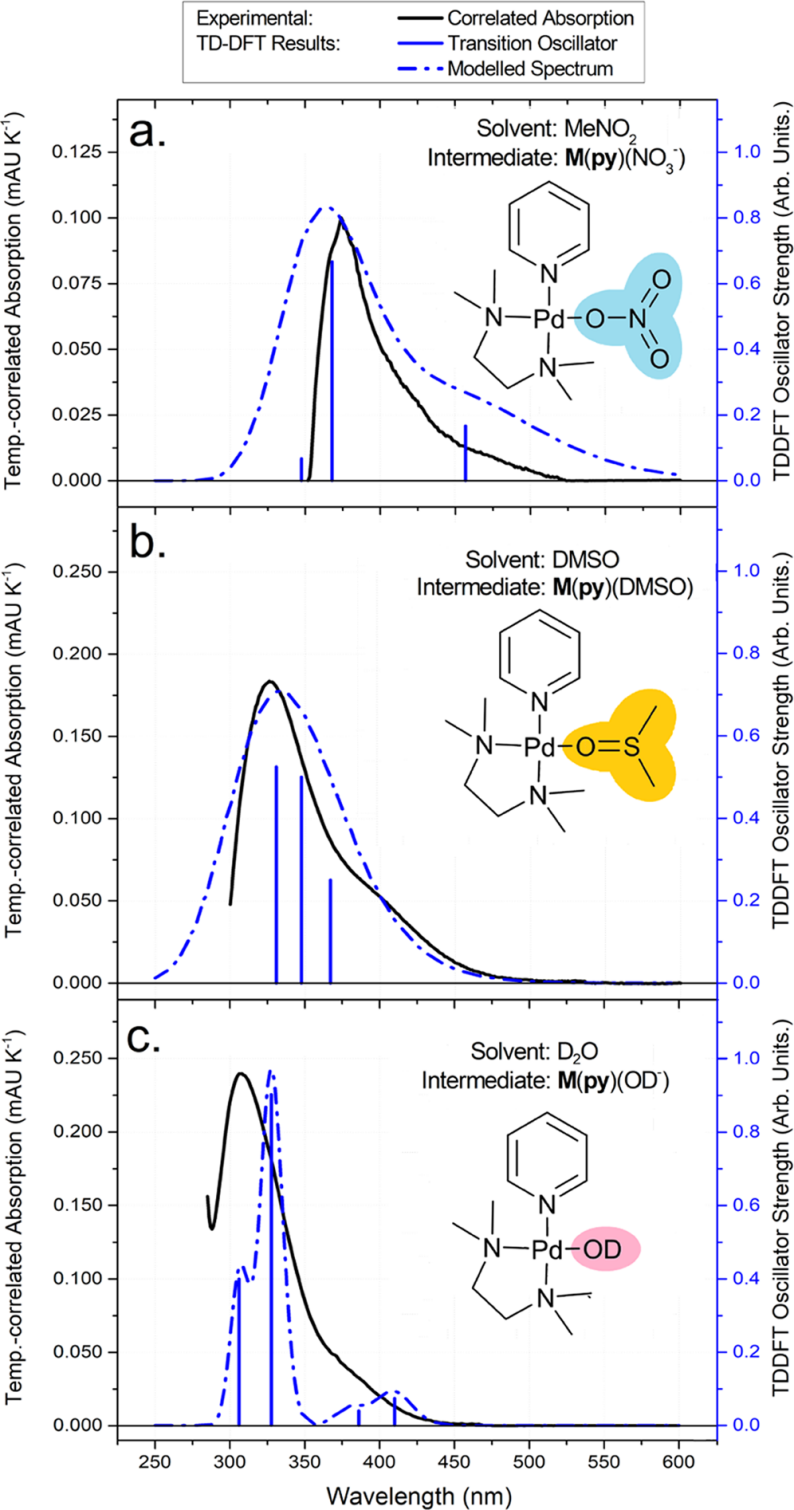
Temperature-correlated absorption spectra of complexes formed from **M**(NO_3_^–^)_2_ (17.3 mM) with excess **py** ([**py**] = 167.3 mM) dissolved in (a) MeNO_2_, (b) DMSO, or (c) D_2_O with TDDFT-computed vertical transitions (Figures S40–S47) of putative intermediate complexes **M**(**py**)(**L**) (**L** = NO_3_^–^, DMSO, or OD^–^, respectively). The TDDFT calculations were carried out at a b3lyp/def2tzv level of theory with CPCM implicit solvation to best replicate experimental conditions.

To support our mechanistic findings, we developed an experimental protocol to directly distinguish solvent or anion-coordinated intermediates that are inaccessible by current approaches; this approach is detailed in Scheme S2. This protocol uses a series of absorption spectra for samples of **M**(**py**)_2_ complexes in different solvents, starting at 300 K and followed by 5–10 elevated temperatures, to reveal regions of linearly increased absorption (Scheme S2). By comparing these temperature-dependent absorption differences to TDDFT calculation results, we could directly interpret the spectra and distinguish solvent or anion-coordinated complexes during pyridyl ligand substitution *in situ* (Figure 6).

The experimental absorption spectra feature narrow peaks within the UV region, suggesting the occurrence of metal-centered *d*–*d* transitions (Figure 6, black traces).^84^ The TDDFT-calculated vertical transitions and extrapolated spectra for the modeled **M**(**py**)(**L**) intermediate complexes (Figure 6a–c, inset) are in good agreement with the observed spectra, enabling qualitative comparison between the two (Figure 6, blue traces). To test the validity of our approach, we applied the same TDDFT methods to oppositional models (i.e., **M**(**py**)(NO_3_^–^) in DMSO), finding that these oppositional vertical transitions were a complete mismatch between coordinating solvents or anions (Figures S40–S44). This approach has a limited ability to distinguish **M**(**py**)(NO_3_^–^) and **M**(**py**)(RO^–^), which have nearly identical computed vertical transitions (Figures S45–S47). However, the consistency between observed spectra and TDDFT vertical transitions supports the presence of **M**(**py**)(**L**) complexes with a coordinated solvent (**L** = MeCN, DMSO) or anion molecules (**L** = NO_3_^–^, RO^–^) as intermediates of pyridyl ligand substitution. These intermediate **M**(**py**)(**L**) complexes are reminiscent of the supramolecular cage formation complexes proposed by Hiraoka et al. as the penultimate intermediate in the self-assembly of Pd-based coordination cages described by his quantitative approaches.^71^ However, the complexes we observe feature endogenous ligands rather than the 3-chloropyridine that were employed in the quantitative studies of cage formation.^71^ As the majority of synthetic protocols lack additive supporting ligands (i.e., 3-chloropyridine), supramolecular cage formation complexes similar to those observed by absorption spectroscopy may serve similarly as the penultimate intermediate in the self-assembly process.

### Thermodynamic Studies of Pyridine Substitution of Stable Intermediates

**Figure 7:**
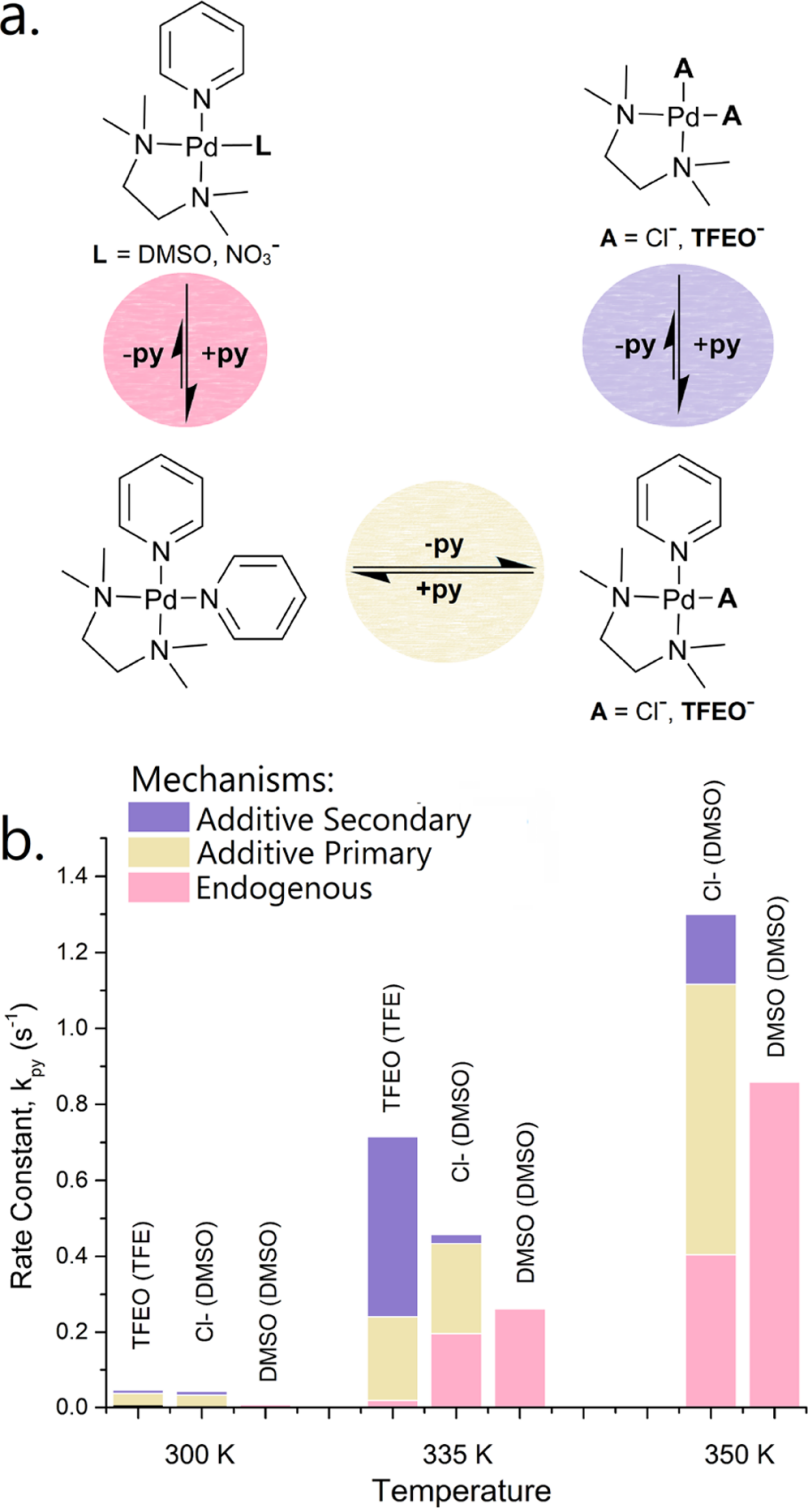
(a) Mechanisms for pyridyl ligand substitution in the presence of additives shaded in yellow and purple (**A** = Cl^–^, **TFEO**^**–**^) but also possibly facilitated by endogenous ligands shaded in pink (**L** = DMSO, NO_3_^–^). (b) Contribution of these three mechanisms to pyridyl ligand substitution at three different temperatures, and under three conditions: **A** = **TFEO**^-^ and TFE solvent; **A** = Cl^–^ and DMSO solvent, or without additive in DMSO for comparison. All three mechanisms were distinguished from EXSY measurements (Figure S34).

Reports describe that TFE and chloride (Cl^–^) catalyze the self-assembly of large Pd-based coordination cages by increasing the rate of ligand substitution at Pd coordination nodes.^69,72,73^ When **M**(**py**)_2_ complexes are formed from **py** (167.3 mM) and **M** (17.3 mM) in neat TFE, or with 8.2 mM tetra(*n*-butyl)ammonium chloride (i.e., Cl^–^) dissolved in DMSO, we observe well-resolved and intense ^1^H-NMR peaks with chemical shifts suggesting the presence of **M**(**py**) complexes bearing **TFEO**^**–**^ or Cl^–^ ligands (Figures S48–S52). Notably, ^19^F-NMR and related DOSY experiments evidence the formation of **M**(**py**)(**TFEO**^**–**^). These **M**(**py**)(**A**) (**A** = **TFEO**^**–**^, Cl^–^) complexes proved to be remarkably stable, resulting in a reliable means of observing the anion-coordinated complex in the solution state (i.e., ^1^H-NMR, EXSY). Therefore, we applied our VT–EXSY approach for monitoring the chemical exchange of **py** arising from **M**(**py**)_2_ and **M**(**py**)(**A**) complexes formed with pure TFE as solvent (**A** = **TFEO**^–^) or with additive tetra(*n*-butyl)ammonium (**A** = Cl^–^) in DMSO (Figure 7).

In our EXSY measurements, the excitation of α′ led to the observation of α and α″ peaks (Figures S31c–S32c), establishing the rate of **py** and **M**(**py**)(**A**) production during the measurement. In the absence of additives, these quantities would be the same (Figure 2); however, in their presence, pyridyl ligand substitution may be facilitated by either the endogenous ligand (Figure 7a, pink) or the additive anion (Figure 7a, yellow). Under these conditions, the **py** production in excess of **M**(**py**)(**A**) corresponds to the endogenously facilitated mechanism, allowing us to quantify the contribution of these two mechanisms to pyridyl ligand substitution (Figure 7b).

Surprisingly, we also observed the dissociation of **py** from **M**(**py**)(**A**) complexes in our VT–EXSY spectra (Figures S31b–S32b). We presume that the dissociation of **py** affords a neutral complex **M**(**A**)_2_, which could not be observed in ^1^H-NMR due to spectral overlap with either TFE or tetra(*n*-butyl)ammonium. Similar to the interconversion between **M**(**py**)_2_ and **M**(**py**)(**A**) (Figure 7a, pink), the interconversion between **M**(**py**)(**A**) and **M**(**A**)_2_ results in pyridyl ligand substitution by a secondary mechanism (Figure 7a, purple). Interestingly, the rate of this secondary **py** substitution at **M**(**py**)(**A**) complexes was similar to or greater than the primary anion-facilitated mechanism at **M**(**py**)_2_ (Figure 7b, yellow). Remarkably, when TFE was used as a solvent, the majority of observed ligand substitution occurred through this additional mechanism (Figure 7b, purple). In contrast, when Cl^–^ is used with DMSO as solvent, both Cl^–^ and DMSO independently facilitate pyridyl ligand substitution. While DMSO-facilitated pyridyl ligand substitution is hampered by additive Cl^–^ (Figure 7b, pink), the total rate is enhanced—by up to 52% at 350 K—from the additive effect of these mechanisms.

We propose that the formation of analogous complexes by Cl^–^/**TFEO**^–^ at Pd^2+^ coordination nodes facilitate defect clearance in the formation of Pd-based coordination cages and similar frameworks (Scheme 1), rationalizing a common mechanism for the reported effects of destabilizing additives in self-assembly.^71−73^ Importantly, the self-assembly of multicomponent constructs (e.g., large coordination cages)^1−60^ would benefit from similar intermediates as they facilitate rate-limiting defect clearance.^51−60^

## Conclusions

In this work, we have developed methods to investigate pyridyl ligand substitution as the rate-limiting process in the self-assembly of coordination cages. Using VT–EXSY and VT absorption spectroscopy, we demonstrated that pyridyl ligand substitution is facilitated by endogenous supporting ligands originating from solvents, counterions, or additives present in the reaction. We found that the polarity, proticity, and coordinating ability of the solvent used dictate the mechanism of pyridyl ligand substitution. When noncoordinating, apolar aprotic solvents are employed, anions present in the solution facilitate pyridyl ligand substitution with an *E*_a_ dependent on ion size (*V*_esp_). Similarly, with protic solvents, *in situ* formed hydroxide or alkoxides lead to an *E*_a_ dependent on solvent p*K*_a_. These anion-facilitated mechanisms of pyridyl ligand substitution are also found when 2,2,2-trifluoroethanol (neat, as a solvent) or tetra(*n*-butyl)ammonium chloride (as additive) are employed, rationalizing the reported effects of these reagents toward enhancing synthetic yields of Pd- and Pt-based coordination cages.

Importantly, our thermodynamic findings have implications to improve the self-assembly of spherical coordination cages and similar frameworks. The defect clearance by pyridyl ligand substitution is enhanced by: (a) the use of coordinating solvents (e.g., DMSO), (b) pairing a small, coordinating anion with a nonpolar, noncoordinating solvent (e.g., NO^3–^ and MeNO_2_), or (c) the use of destabilizing additives such as Cl^–^ or TFE. Our findings may be similarly leveraged to improve cage stability or induce the formation of kinetically trapped species by removing endogenous ligand sources to increase the *E*_a_ of pyridyl ligand substitution.

## Supporting Information Available

Method, experimental data (including NMR, VT absorption, and CSI-HRMS spectra), and computational model details (PDF)
