# Supplementary material for: Exposing Mechanisms for Defect Clearance in Supramolecular Self-Assembly: Palladium–Pyridine Coordination Revisited
Source: Inorg Chem. 2023 Mar 24;62(14):5458–67. doi: 10.1021/acs.inorgchem.2c04404 (PMC10091417; doi:10.1021/acs.inorgchem.2c04404)
Supplement: Supplementary file 1 — ic2c04404_si_001.pdf [file ic2c04404_si_001.pdf]

Supporting Information for:

Exposing mechanisms for defect clearance in supramolecular self-assembly: Palladium–pyridine coordination revisited.

David A. Poole III, Eduard O. Bobylev, Bas de Bruin, Simon Mathew, and Joost N. H. Reek*

Homogeneous, Supramolecular, and Bioinspired Catalysis group, van ’t Hoff Institute for Molecular Science (HIMS), University of Amsterdam (UvA), Science Park 904, 1098 XH Amsterdam, The Netherlands

J.N.H.Reek@uva.nl

Contents

A. Synthesis and preparation of **M** S3
B. DOSY Characterization of **M**(**py**) in DMSO. S4
C. ^{1}H-NMR and DOSY characterization of **M**(**py**)$_2$ complexes at different **py** concentrations. S5
D. ESI–HRMS analysis of **M**(**py**)(NO_3^-) complexes S7
E. EXSY analysis of **M**(**py**)$_2$ complexes at different **py** concentrations. S8
F. V_{esp} method and visualization S23
F. GFN2-xTB Simulation of pyridine protonation S24
G. Molecular dynamics simulations S24
H. ^{1}H-NMR characterization of **M**(**py**)(NO_3^-) intermediate complexes S25
I. Variable temperature absorption spectroscopy and TDDFT S26
J. ^{1}H-NMR and DOSY characterization of **M**(**py**)(Cl^-) S31
K. ^{1}H-, ^{19}F-NMR characterization of **M**(**py**)(**TFEO**$^-$) S32

Solvents (Acetonitrile, Acetone), drying agents (sodium sulfate) and nitric acid (conc. 15.6 M) were purchased from VWR. Acetone was dried directly before use by vigorous stirring with sodium sulfate (Na_2SO_4, 10 g L^{-1}), which was removed by vacuum filtration (glass frit, porosity 4). Nitric acid (100 µL, 15.6 M) was diluted with purified water (MilliQ, 18.1 mΩ) in a volumetric flask (100 mL). Deuterated DMSO (99.0 %D, DMSO-d_6) was purchased from Merck (SKU: 151874) and used as delivered.

Purchased reagents, dichloro(*N*,*N*,*N*',*N*'-tetramethylethylenediamine)palladium(II) (Merck, SKU: 345229), $AgNO_3$ (Fluorochem, SKU: 080282), and 4,4'-dipyridyl (TCI, B0469) were used as delivered.

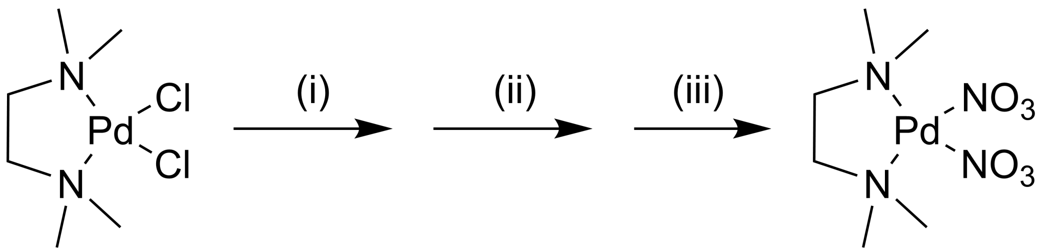

Scheme S1. Overview for the synthesis and purification of dinitrato(*N,N,N',N'*-tetramethylethylenediamine)palladium(II) (**M**), from dichloro(*N,N,N',N'*-tetramethylethylenediamine)palladium dichloride. Conditions: (i) 2.05 eq. $AgNO_3$, cat. HNO_3, MeCN/H_2O 1:1, dark, stir 72 h; (ii) filter to remote precipitated AgCl, dry; (iii) dissolve in acetone, filter to remove insoluble $AgNO_3$, dry.

M: The coordination node, dinitrato(*N*,*N*,*N*',*N*'-tetramethylethylenediamine)palladium(II) (**M**), was synthesized and purified following a modified literature procedure.[1] Briefly, dichloro(*N*,*N*,*N*',*N*'-tetramethylethylenediamine)palladium(II) (1.00 g, 3.4 mmol) was suspended in acetonitrile (100 mL) in a 250 mL foil-wrapped amber round-bottom flask and stirred until a clear yellow solution was obtained. Then, a solution of $AgNO_3$ (1.19 g, 7.0 mM) in dilute aqueous nitric acid (1.5 mM, 100 mL), was added by an ordinary funnel, resulting in the immediate formation of a white precipitate (AgCl). The flask was closed by glass stopper and left stirring under ambient conditions (ca. 72 h). The reaction mixture was then filtered by gravity to remove precipitated AgCl (Whatman Grade 313), and the filtrate dried to afford an off-yellow residue. This residue was suspended in dried acetone (500 mL) and vigorously stirred under a dry nitrogen atmosphere (2 h). The resulting yellow solution was filtered (0.44 µm), and dried to afford (1.01 g, 86% yield) the title compound as a yellow crystalline powder. ^{1}H-NMR (300 MHz, DMSO-d_6) δ 2.55 (6 H), 2.80 (2 H).

B. DOSY Characterization of **M**(**py**) in DMSO.

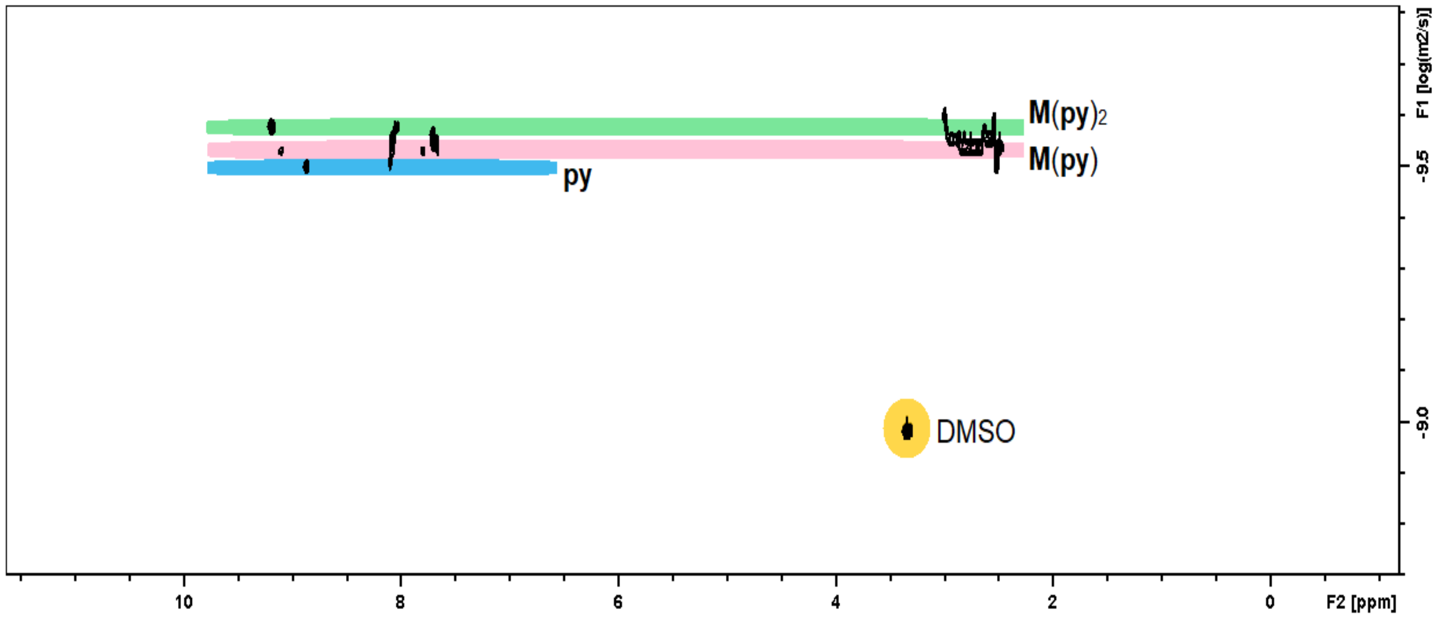

Figure S1. Diffusogram of **M**(**py**)$_2$ (green), **M**(**py**) (pink), and **py** (blue) computed from the variable gradient pulse sequence (300 MHz, DMSO-d_6, 298 K, Δt = 100 ms, δ = 1 ms, 19 points, ledbpgp2s) using the dosy2d program in Topspin 4.0.9.

C. ^{1}H-NMR and DOSY characterization of **M**(**py**)$_2$ complexes at different **py** concentrations.

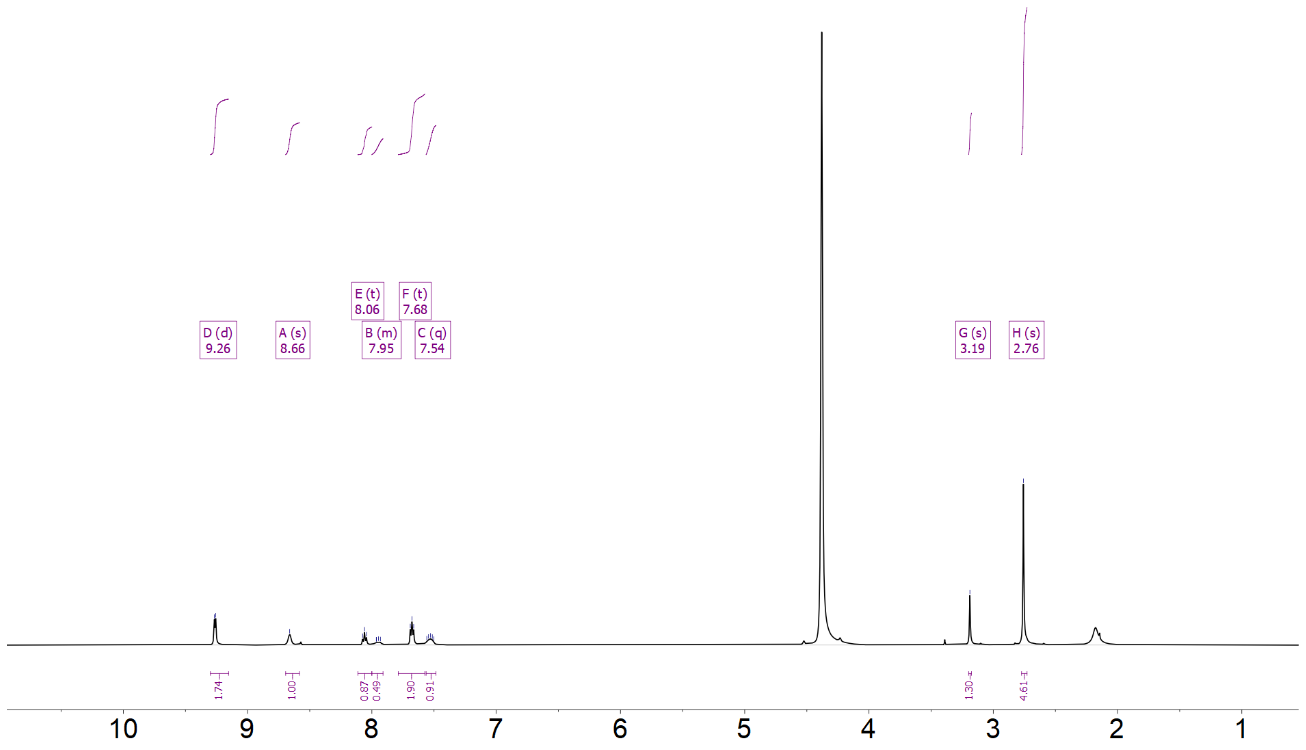

Figure S2. ^{1}H-NMR spectrum (300 MHz) of **M**(**py**)$_2$ complexes formed with **py** (58.3 mM) and **M** (17.3 mM) in MeNO$_2$-d_3.

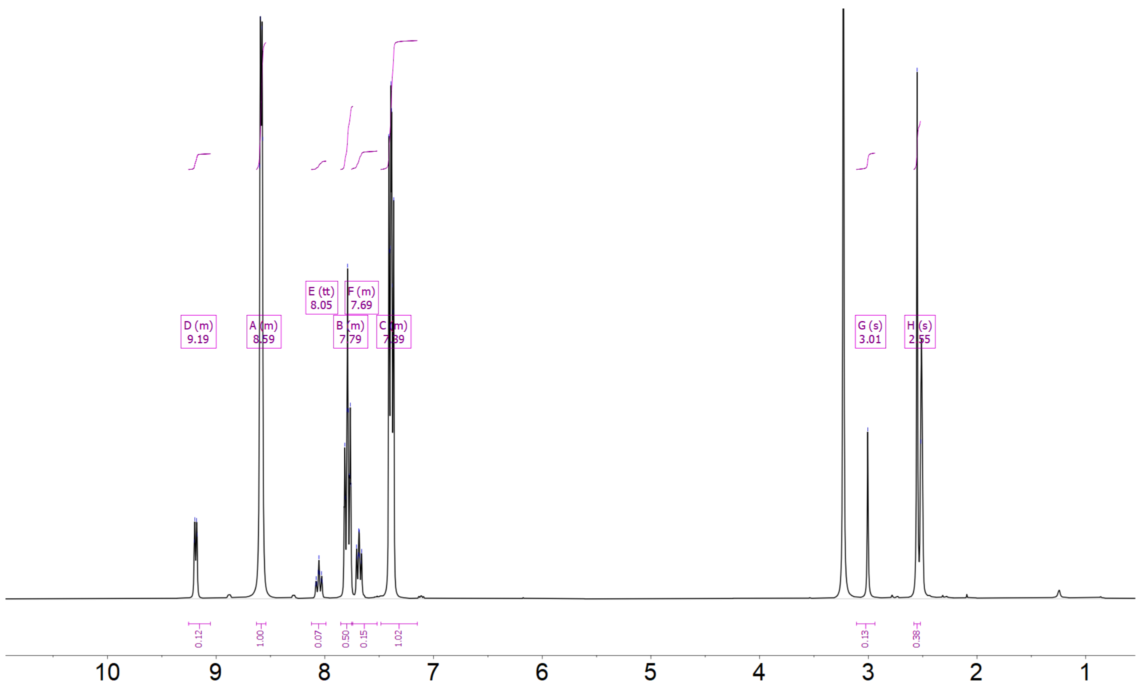

Figure S3. ^{1}H-NMR spectrum (300 MHz) of **M**(**py**)$_2$ complexes formed with **py** (167.3 mM) and **M** (17.3 mM) in MeNO$_2$-d_3.

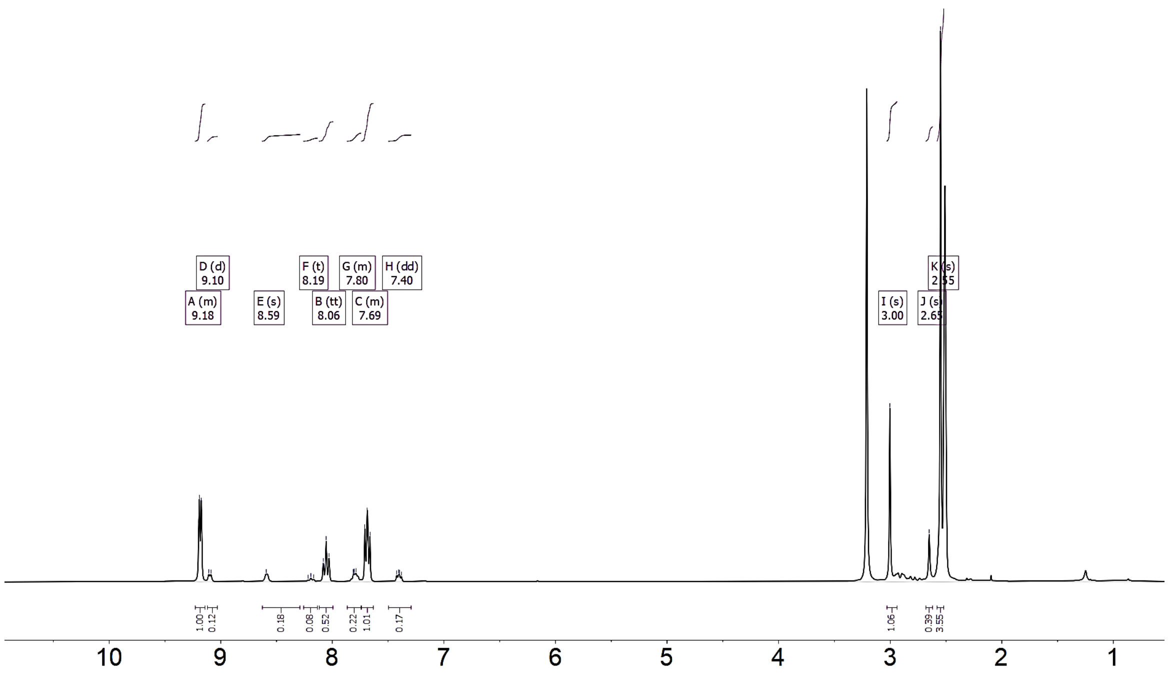

Figure S4.^{1}H-NMR spectrum (300 MHz) of **M**(**py**)$_2$ complexes formed with **py** (58.3 mM) and **M** (17.3 mM) in DMSO-d_6.

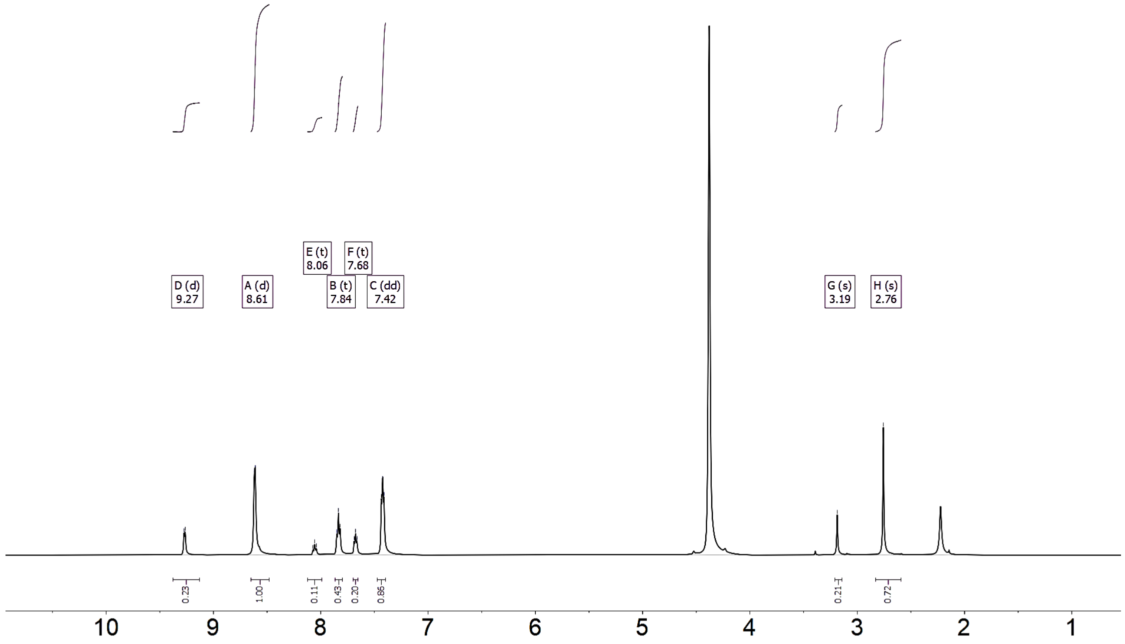

Figure S5. ^{1}H-NMR spectrum (300 MHz) of **M**(**py**)$_2$ complexes formed with **py** (167.3 mM) and **M** (17.3 mM) in DMSO-d_6.

D. ESI–HRMS analysis of **M**(**py**)(NO_3^-) complexes

Analysis method: Electrospray ionization mass spectrometry (ESI–MS): Mass spectra were collected on a HR-ToF Bruker Daltonik GmbH (Bremen, Germany) Impact II, an ESI–ToF–MS capable of resolution of at least 40000 FWHM, which was coupled to a Bruker cryospray unit. Detection was in positive-ion mode and the source voltage was between 4 and 6 kV. The sample was introduced with a syringe pump at a flow rate of 18 µL hr^{-1}. The drying gas (N_2) was held at 40°C and the spray gas was held at 60°C. The machine was calibrated prior to every experiment via direct infusion of a Na(TFA) solution, which provided a *m/z* range of singly charged peaks up to 3500 Da. in both ion modes. Software acquisition using Compass 2.0 for OTOF series with further processing by m-mass for simulation data.

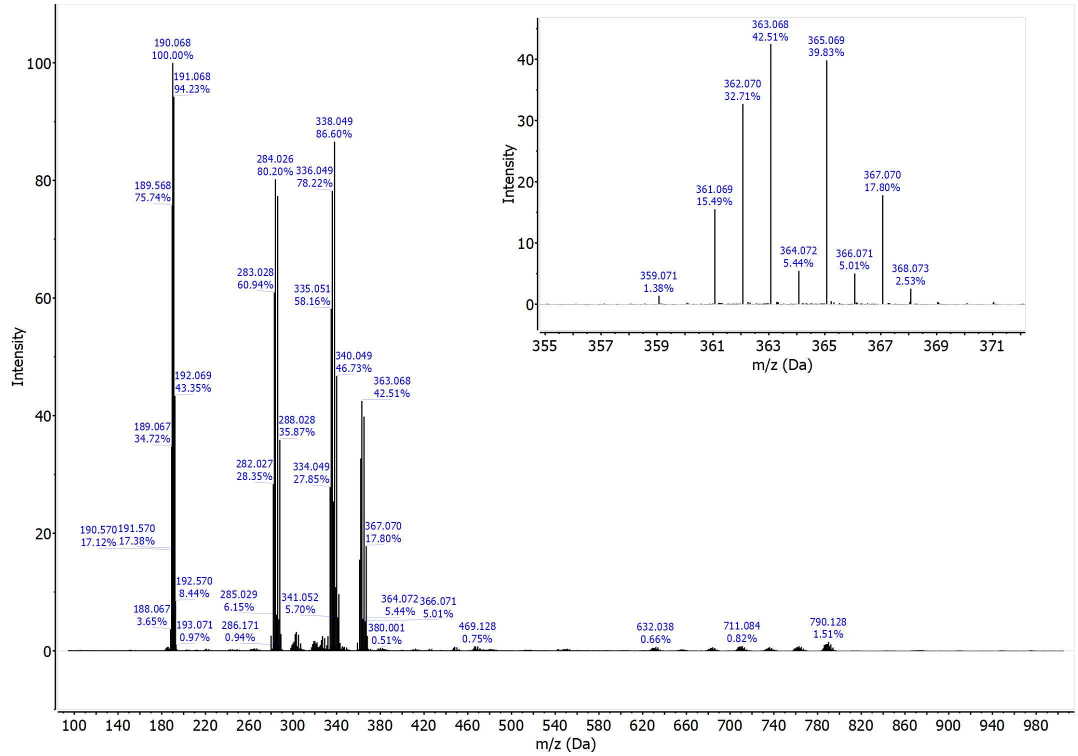

Figure S6. ESI–HRMS spectra obtained after the injection of the mixture of complexes afforded from **py** (58.3 mM) and **M** (17.3 mM) in $MeNO_2$. Inset shows a species with the isotope distribution pattern of **M**(**py**)(NO_3^-)$^+$, matched below.

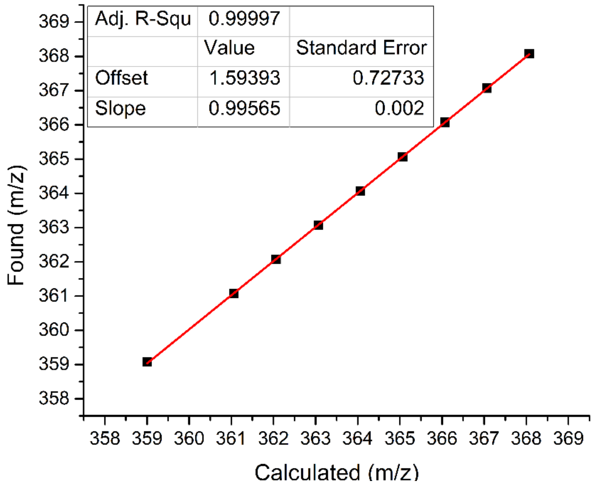

Figure S7. Plot comparing the found and calculated isotope distribution of **M**(**py**)(NO_3^-)$^+$ ($C_{11}H_{21}N_4O_3Pd^+$). The linearity of the plot indicates a good match between the calculated and observed mass spectrum.

E. EXSY analysis of **M**(**py**)$_2$ complexes at different **py** concentrations.

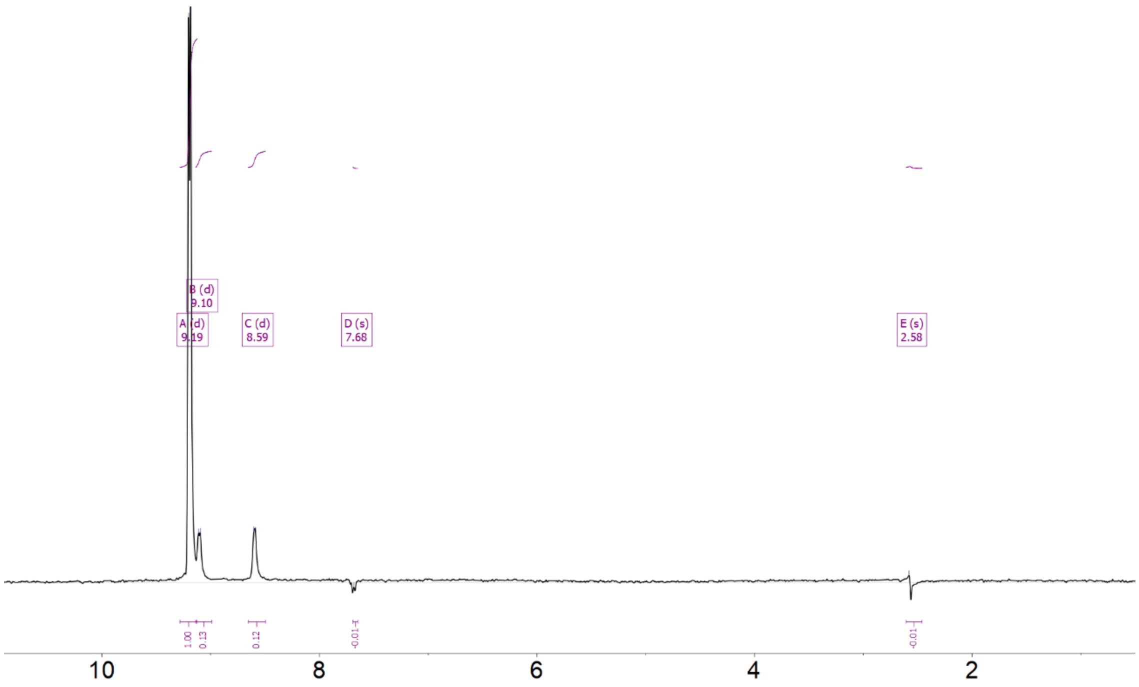

Figure S9. EXSY spectrum (300 MHz) showing chemical exchange (and NOE) arising from excitation of the α-pyridyl peak of **M**(**py**)$_2$ (i.e., α'). Complexes were formed with **py** (58.3 mM) and **M** (17.3 mM) in $MeNO_2$-d_3.

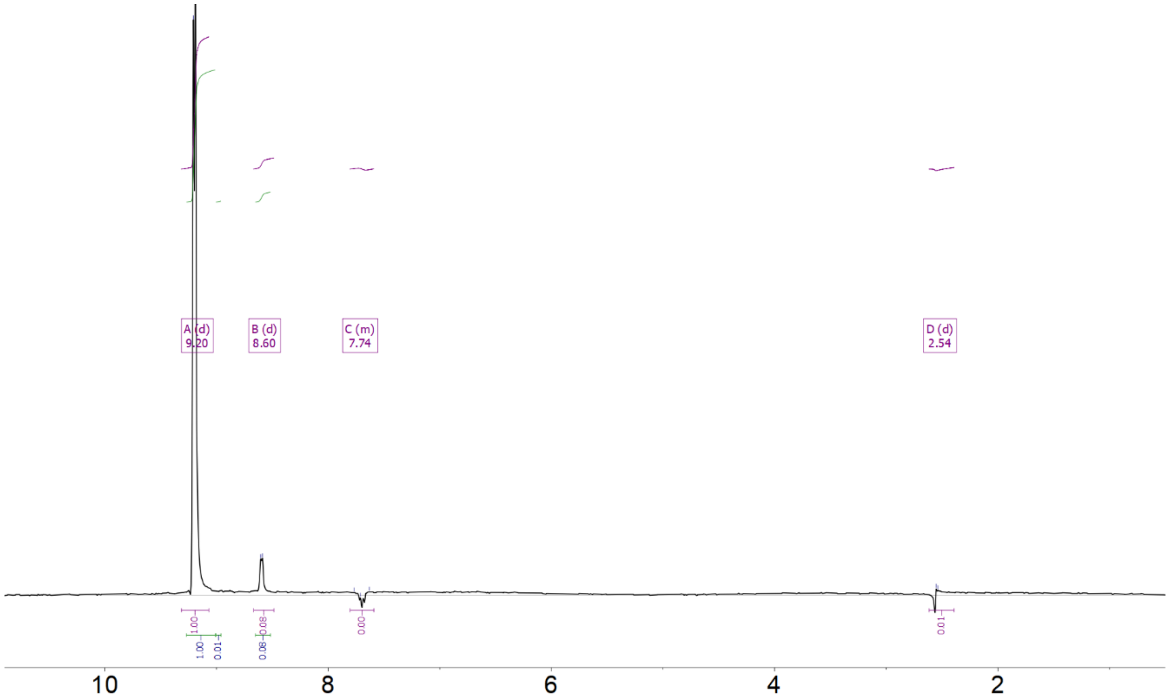

Figure S10. EXSY spectrum (300 MHz) showing chemical exchange (and NOE) arising from excitation of the α-pyridyl peak of **M**(**py**)$_2$ (i.e., α'). Complexes were formed 167.3 mM **py** and **M** (17.3 mM) in $MeNO_2$-d_3.

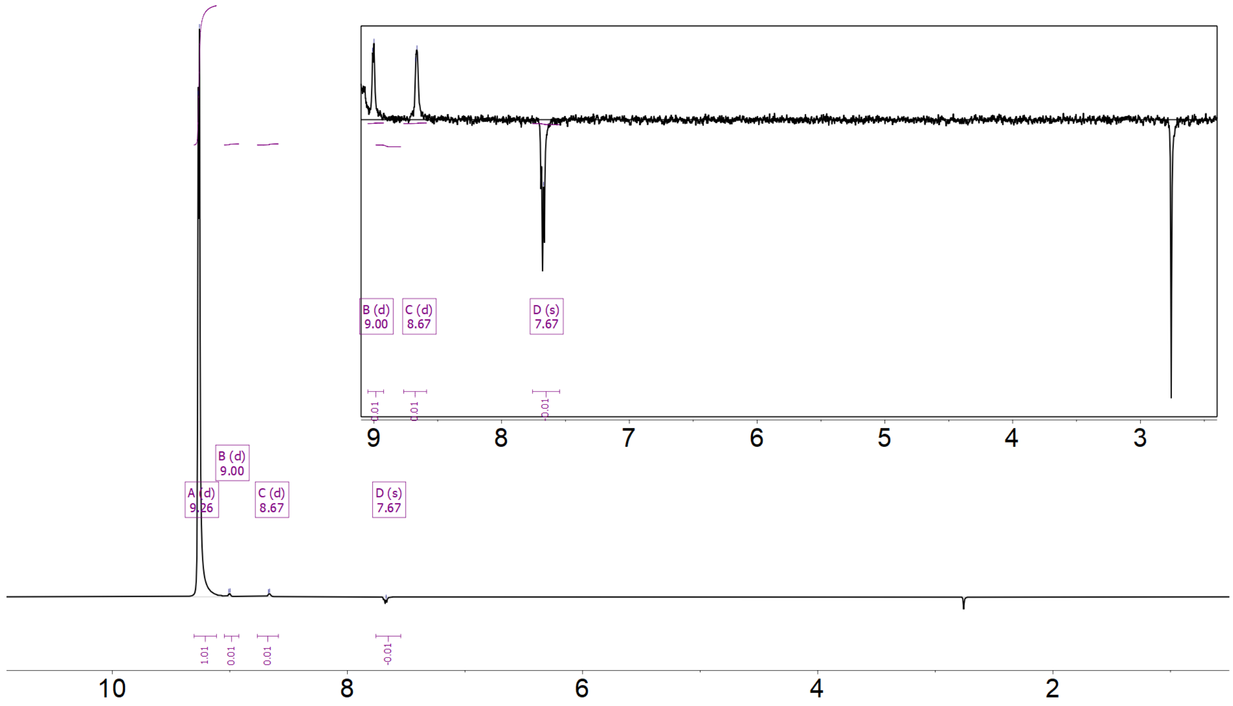

Figure S11. EXSY spectrum (300 MHz) showing chemical exchange (and NOE) arising from excitation of the α-pyridyl peak of **M**(**py**)$_2$ (i.e., α'). Complexes were formed with **py** (58.3 mM) and **M** (17.3 mM) in DMSO-d_6.

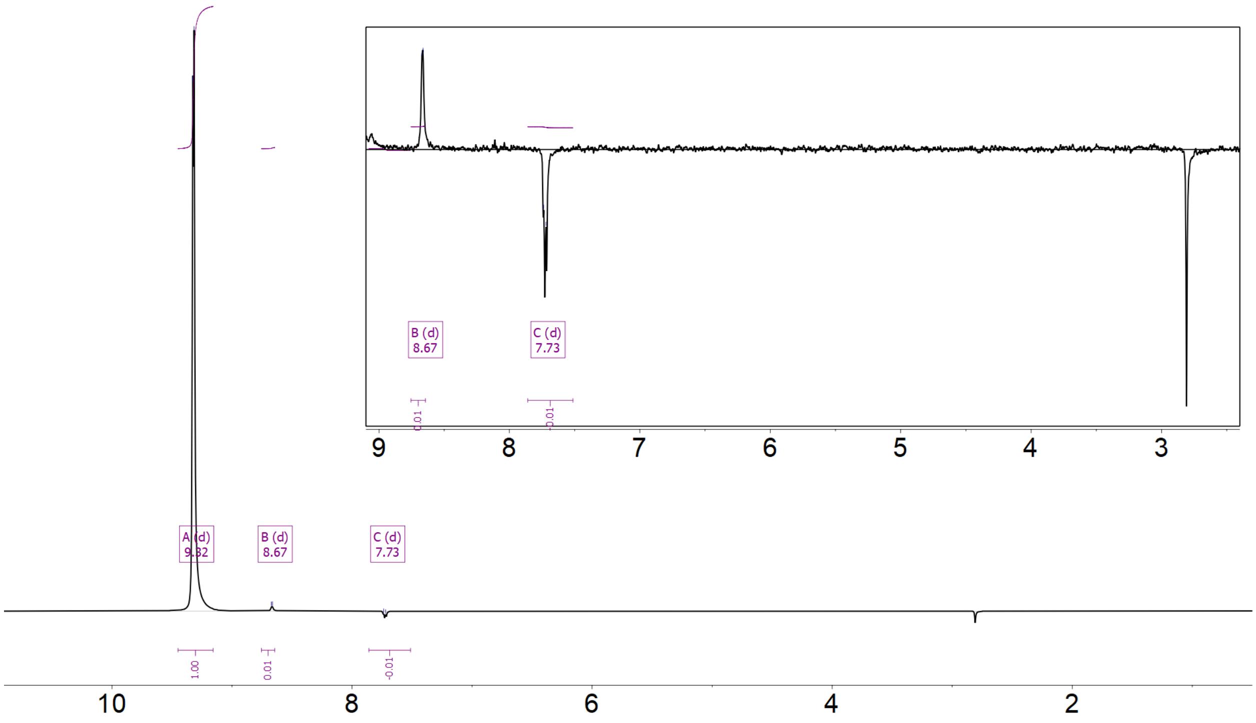

Figure S12. EXSY spectrum (300 MHz) showing chemical exchange (and NOE) arising from excitation of the α-pyridyl peak of **M**(**py**)$_2$ (i.e., α'). Complexes were formed 167.3 mM **py** and **M** (17.3 mM) in DMSO-d_6.

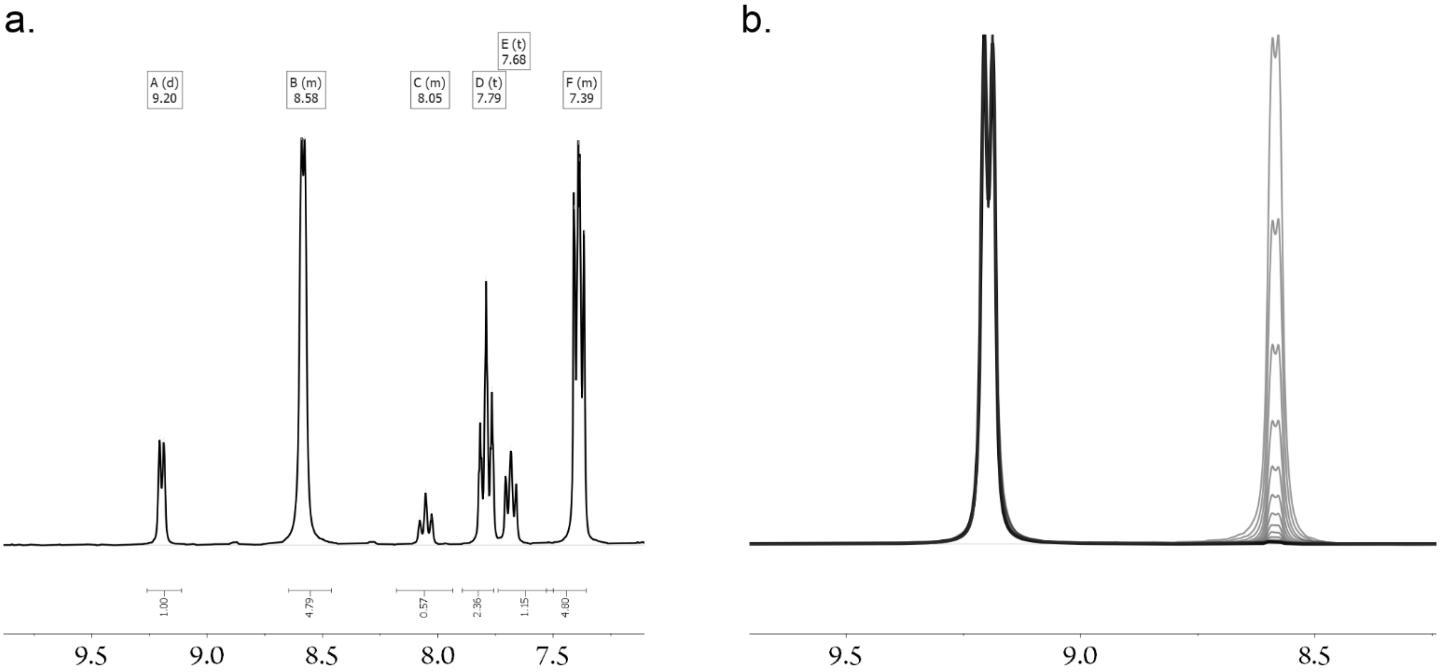

Figure S13. (a) α-pyridyl region ^{1}H-NMR spectrum (300 MHz) of **M**(**py**)$_2$(NO_3^-)$_2$ complexes formed with excess pyridine (167.3 mM) in DMSO-*d*$_6$. (b) VT-NMR spectra (normalized and overlaid) used to monitor the dissociation of **py** from **M**(**py**)$_2$ at temperatures between 300–355 K in 5 K increments with 10 minutes between each sample measurement. Complexes were formed by the complexation of **M**(NO_3^-)$_2$ ([**M**] = 17.3 mM) with excess **py** ([**py**] = 167.3 mM).

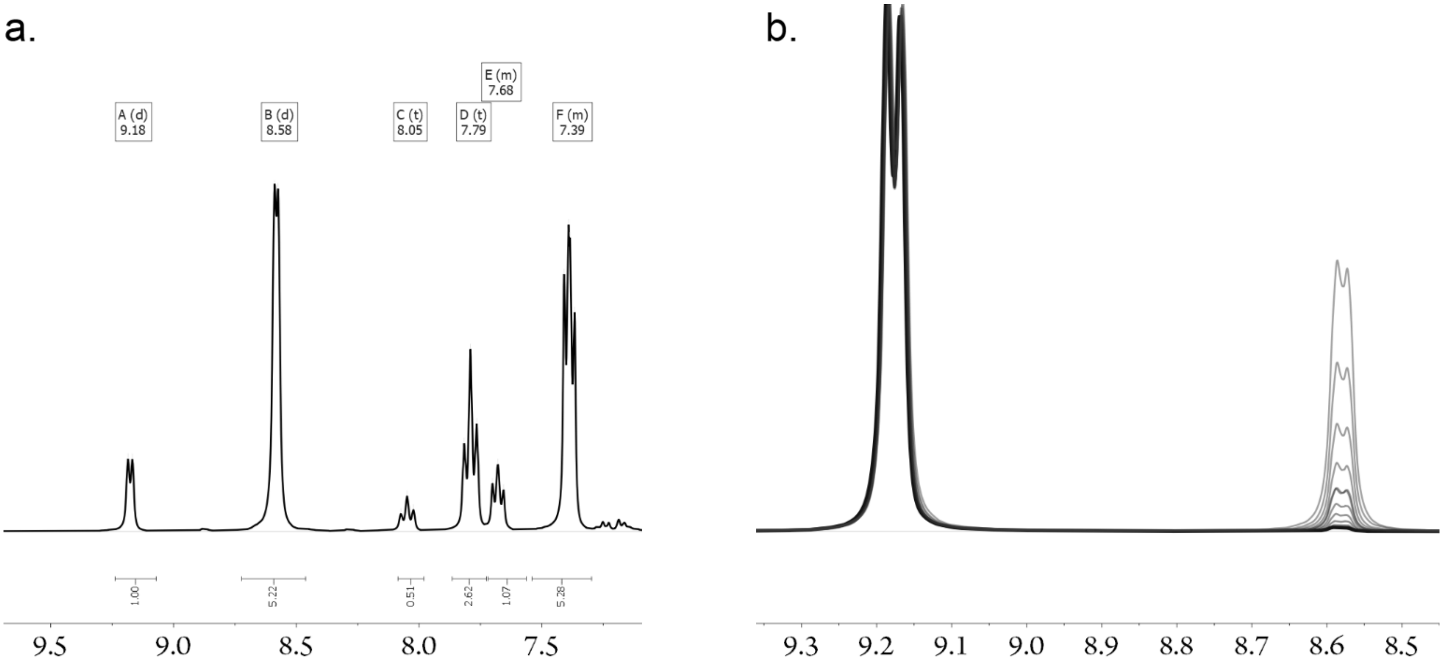

Figure S14. (a) α-pyridyl region ^{1}H-NMR spectrum (300 MHz) of **M**(**py**)$_2$(ClO_4^-)$_2$ complexes formed with excess pyridine (167.3 mM) in DMSO-*d*$_6$. (b) VT-NMR spectra (normalized and overlaid) used to monitor the dissociation of **py** from **M**(**py**)$_2$ at temperatures between 300–355 K in 5 K increments with 10 minutes between each sample measurement. Complexes were formed by the complexation of **M**(NO_3^-)$_2$ ([**M**] = 17.3 mM) with excess **py** ([**py**] = 167.3 mM).

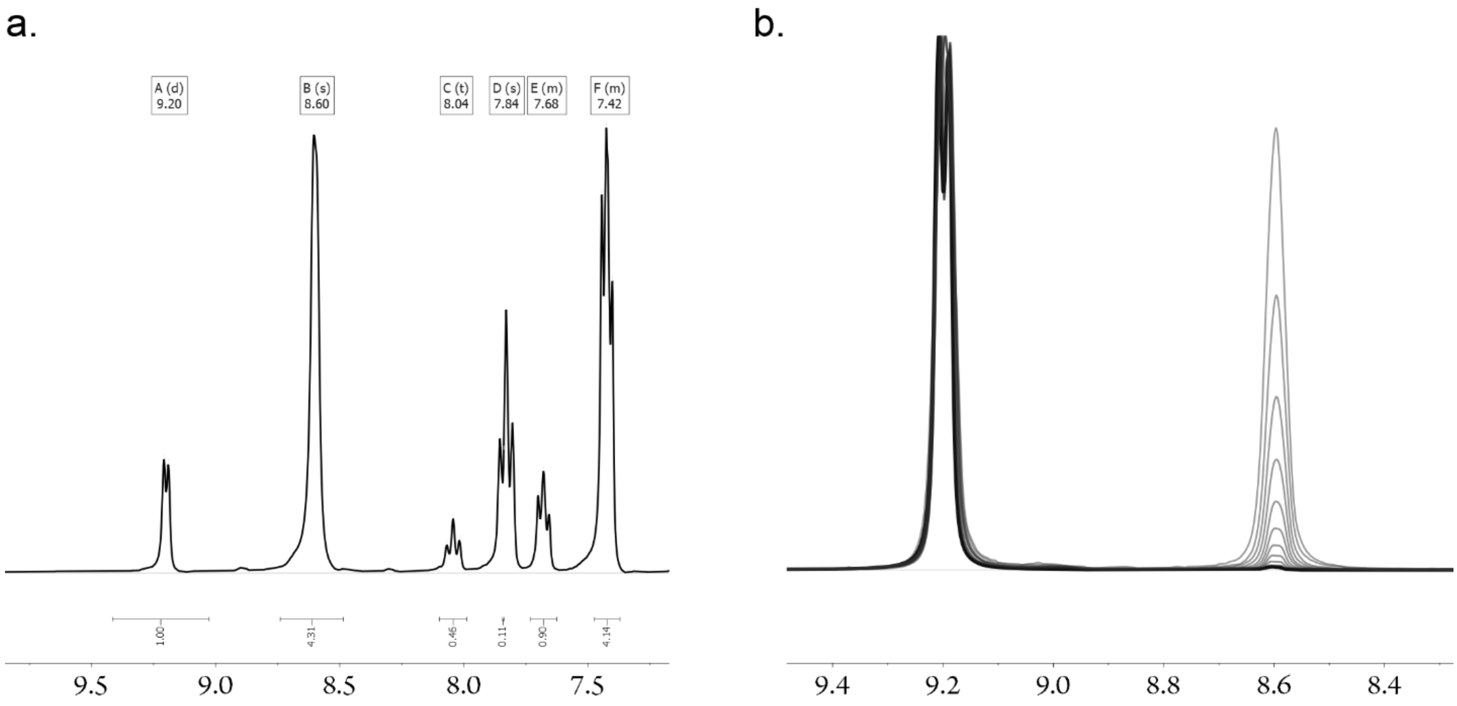

Figure S15. (a) α-pyridyl region ^{1}H-NMR spectrum (300 MHz) of **M**(**py**)$_2$(PF_6^-)$_2$ complexes formed with excess pyridine (167.3 mM) in DMSO-d_6. (b) VT-NMR spectra (normalized and overlaid) used to monitor the dissociation of **py** from **M**(**py**)$_2$ at temperatures between 300–355 K in 5 K increments with 10 minutes between each sample measurement. Complexes were formed by the complexation of **M**(NO_3^-)$_2$ ([**M**] = 17.3 mM) with excess **py** ([**py**] = 167.3 mM).

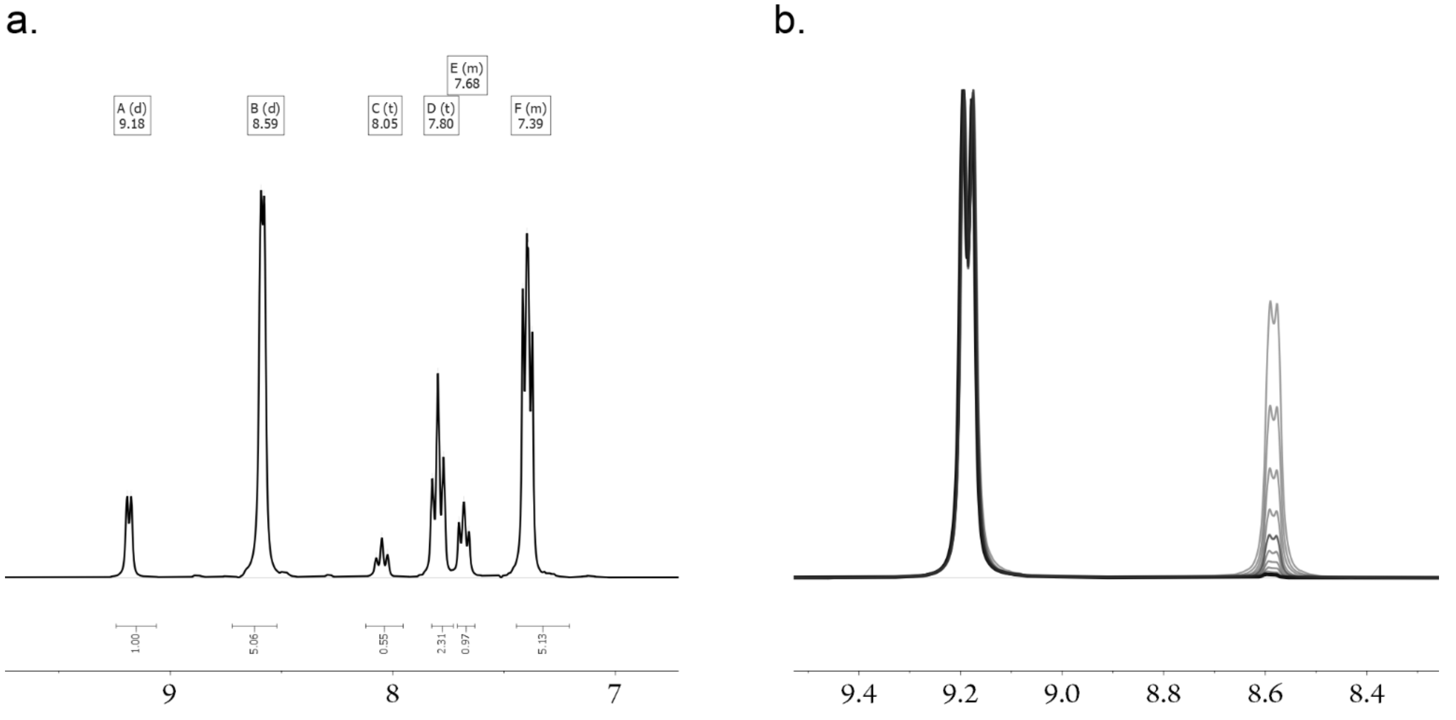

Figure S16. (a) α-pyridyl region ^{1}H-NMR spectrum (300 MHz) of **M**(**py**)$_2$(BF_4^-)$_2$ complexes formed with excess pyridine (167.3 mM) in DMSO-d_6. (b) VT-NMR spectra (normalized and overlaid) used to monitor the dissociation of **py** from **M**(**py**)$_2$ at temperatures between 300–355 K in 5 K increments with 10 minutes between each sample measurement. Complexes were formed by the complexation of **M**(NO_3^-)$_2$ ([**M**] = 17.3 mM) with excess **py** ([**py**] = 167.3 mM).

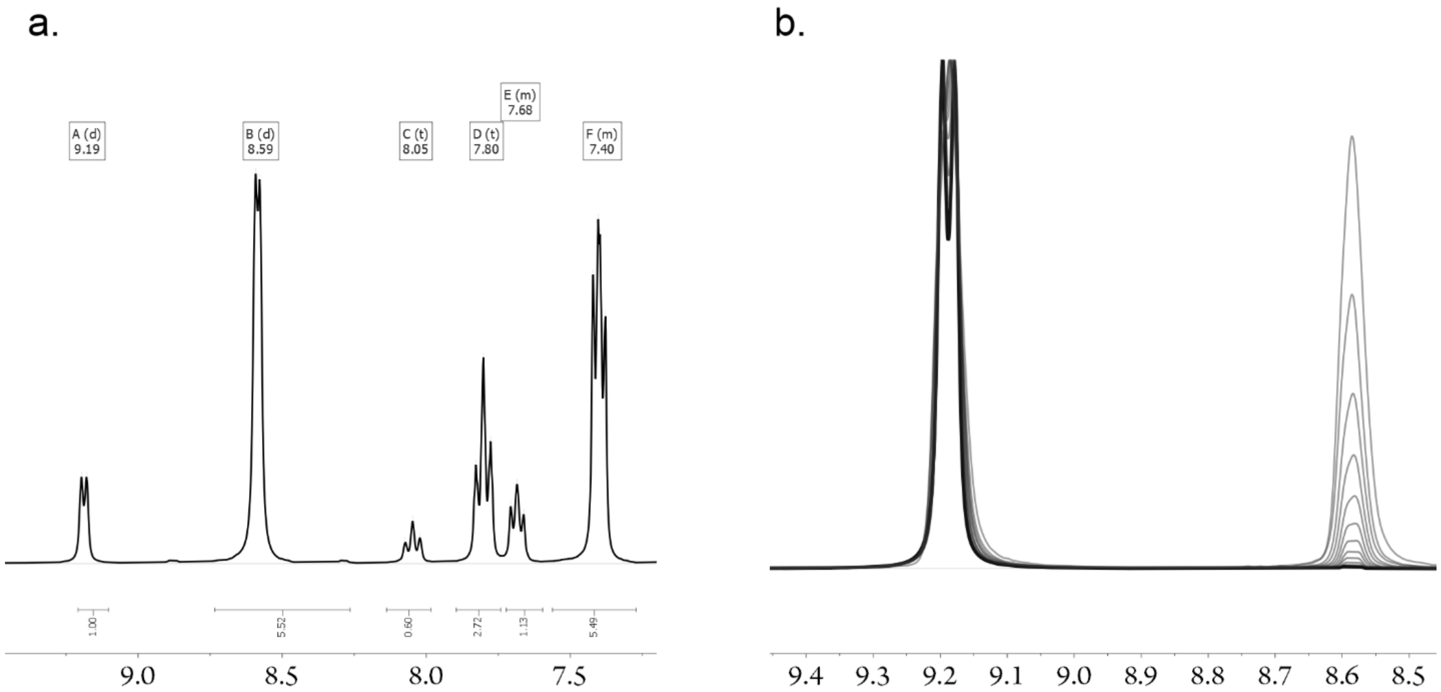

Figure S17. (a) α-pyridyl region ^{1}H-NMR spectrum (300 MHz) of **M**(**py**)$_2$(OTf$^-$)$_2$ complexes formed with excess pyridine (167.3 mM) in DMSO-d_6. (b) VT-NMR spectra (normalized and overlaid) used to monitor the dissociation of **py** from **M**(**py**)$_2$ at temperatures between 300–355 K in 5 K increments with 10 minutes between each sample measurement. Complexes were formed by the complexation of **M**(NO$_3^-$)$_2$ ([**M**] = 17.3 mM) with excess **py** ([**py**] = 167.3 mM).

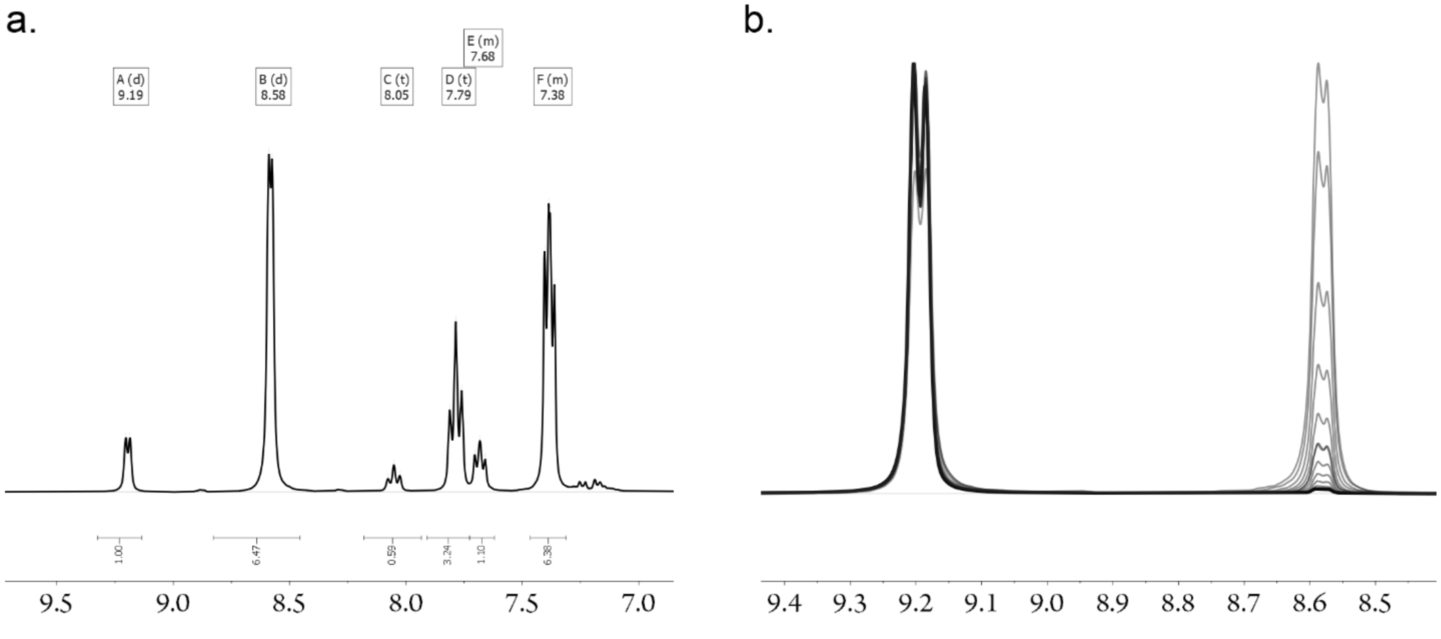

Figure S18. (a) α-pyridyl region ^{1}H-NMR spectrum (300 MHz) of **M**(**py**)$_2$(IO$_4^-$)$_2$ complexes formed with excess pyridine (167.3 mM) in DMSO-d_6. (b) VT-NMR spectra (normalized and overlaid) used to monitor the dissociation of **py** from **M**(**py**)$_2$ at temperatures between 300–355 K in 5 K increments with 10 minutes between each sample measurement. Complexes were formed by the complexation of **M**(NO$_3^-$)$_2$ ([**M**] = 17.3 mM) with excess **py** ([**py**] = 167.3 mM).

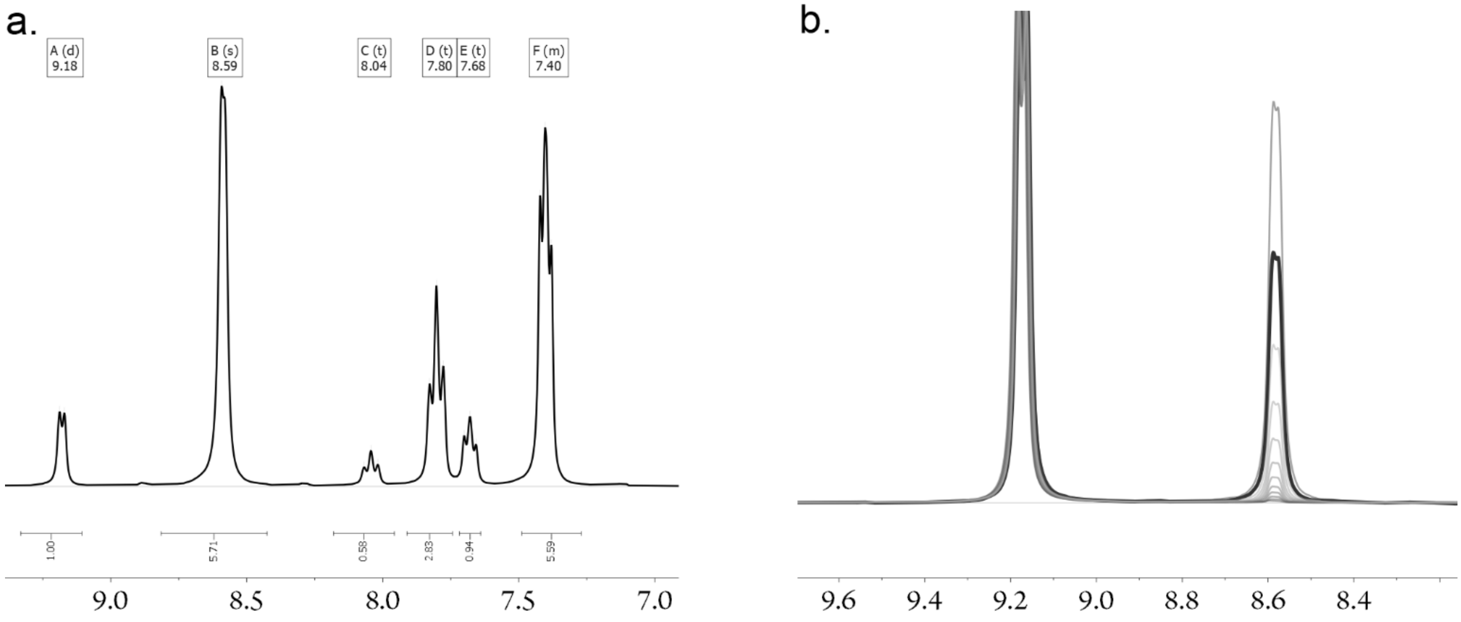

Figure S19. (a) α-pyridyl region ^{1}H-NMR spectrum (300 MHz) of **M**(**py**)$_2$(SbF_6^-)$_2$ complexes formed with excess pyridine (167.3 mM) in DMSO-d_6. (b) VT-NMR spectra (normalized and overlaid) used to monitor the dissociation of **py** from **M**(**py**)$_2$ at temperatures between 300–355 K in 5 K increments with 10 minutes between each sample measurement. Complexes were formed by the complexation of **M**(NO_3^-)$_2$ ([**M**] = 17.3 mM) with excess **py** ([**py**] = 167.3 mM).

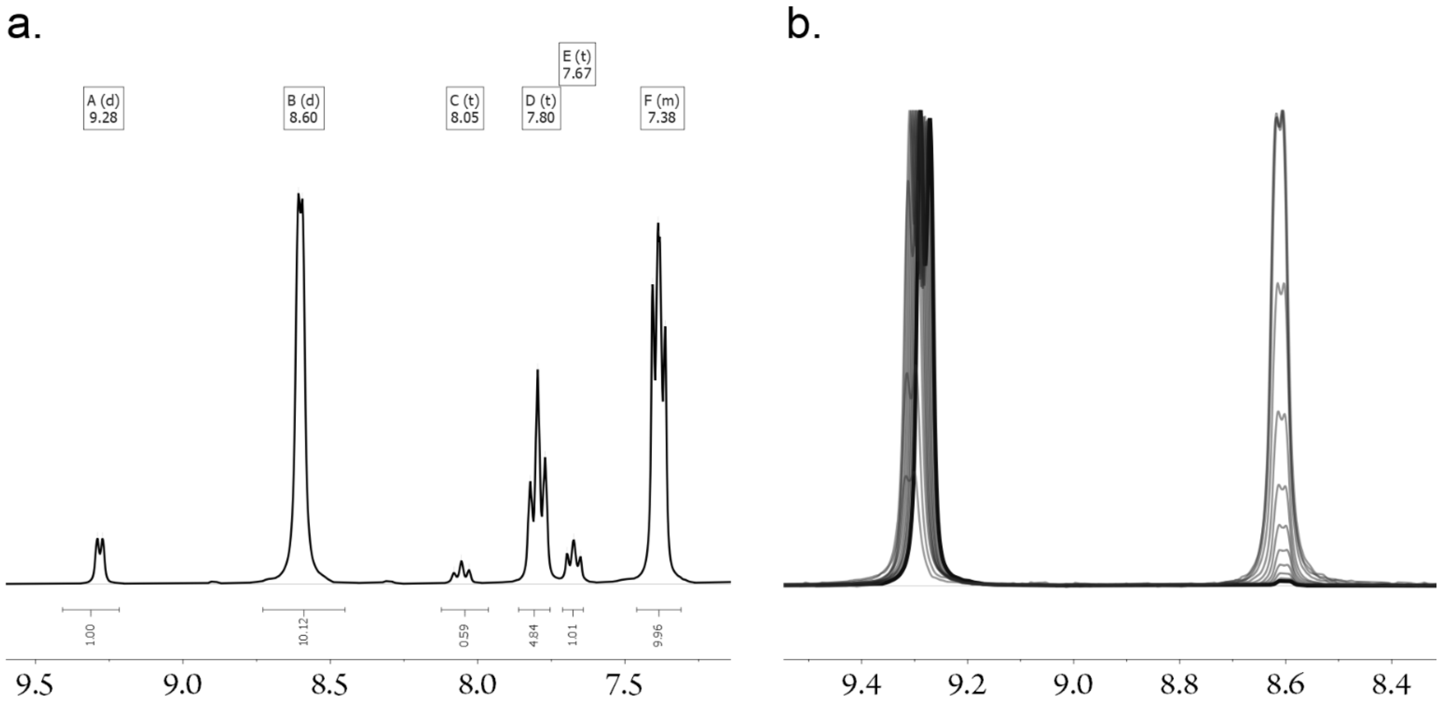

Figure S20. (a) α-pyridyl region ^{1}H-NMR spectrum (300 MHz) of **M**(**py**)$_2$(NO_3^-)$_2$ complexes formed with excess pyridine (167.3 mM) in $MeNO_2$-d_3. (b) VT-NMR spectra (normalized and overlaid) used to monitor the dissociation of **py** from **M**(**py**)$_2$ at temperatures between 300–355 K in 5 K increments with 10 minutes between each sample measurement. Complexes were formed by the complexation of **M**(NO_3^-)$_2$ ([**M**] = 17.3 mM) with excess **py** ([**py**] = 167.3 mM).

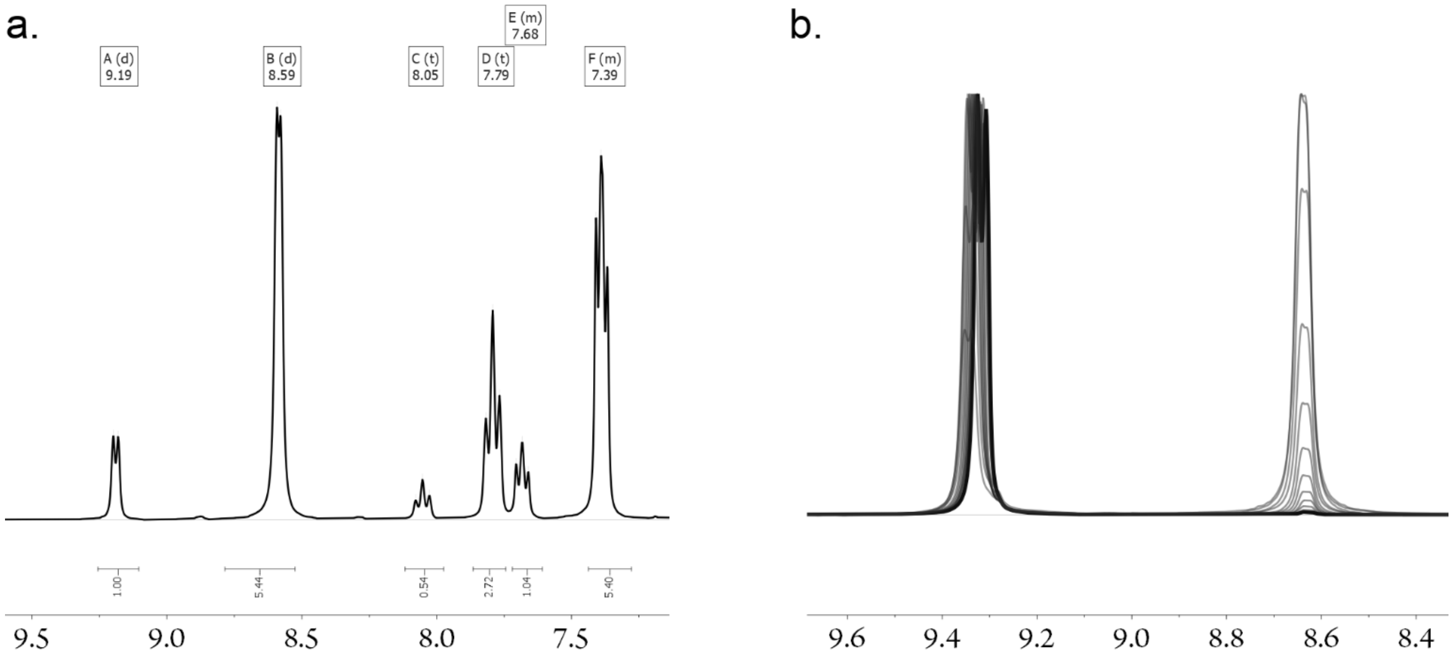

Figure S21. (a) α-pyridyl region ^{1}H-NMR spectrum (300 MHz) of **M**(**py**)$_2$(ClO_4^-)$_2$ complexes formed with excess pyridine (167.3 mM) in MeNO$_2$-*d*$_3$. (b) VT-NMR spectra (normalized and overlaid) used to monitor the dissociation of **py** from **M**(**py**)$_2$ at temperatures between 300–355 K in 5 K increments with 10 minutes between each sample measurement. Complexes were formed by the complexation of **M**(NO_3^-)$_2$ ([**M**] = 17.3 mM) with excess **py** ([**py**] = 167.3 mM).

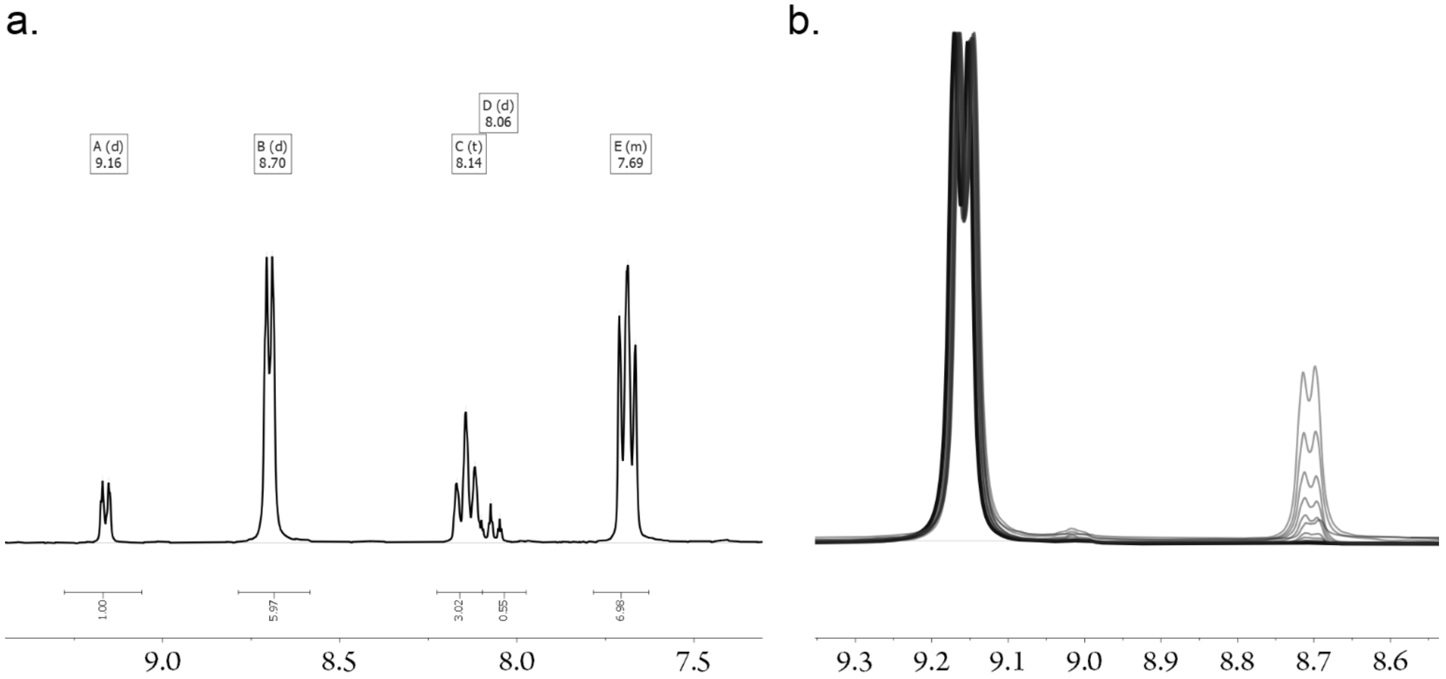

Figure S22. (a) α-pyridyl region ^{1}H-NMR spectrum (300 MHz) of **M**(**py**)$_2$(BF_4^-)$_2$ complexes formed with excess pyridine (167.3 mM) in MeNO$_2$-*d*$_3$. (b) VT-NMR spectra (normalized and overlaid) used to monitor the dissociation of **py** from **M**(**py**)$_2$ at temperatures between 300–355 K in 5 K increments with 10 minutes between each sample measurement. Complexes were formed by the complexation of **M**(NO_3^-)$_2$ ([**M**] = 17.3 mM) with excess **py** ([**py**] = 167.3 mM).

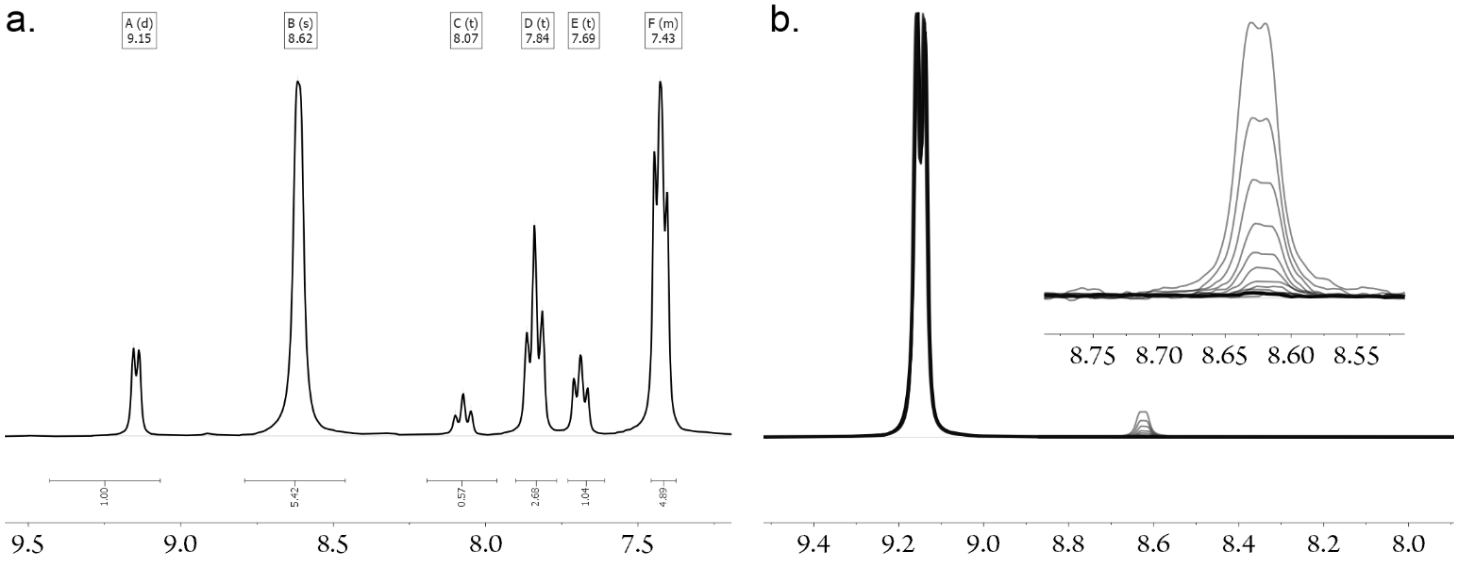

Figure S23. (a) α-pyridyl region ^{1}H-NMR spectrum (300 MHz) of **M**(**py**)$_2$(PF_6^-)$_2$ complexes formed with excess pyridine (167.3 mM) in $MeNO_2$-d_3. (b) VT-NMR spectra (normalized and overlaid) used to monitor the dissociation of **py** from **M**(**py**)$_2$ at temperatures between 300–355 K in 5 K increments with 10 minutes between each sample measurement. Complexes were formed by the complexation of **M**(NO_3^-)$_2$ ([**M**] = 17.3 mM) with excess **py** ([**py**] = 167.3 mM). Inset shown to magnify evolved **py** peak arising from dissociation.

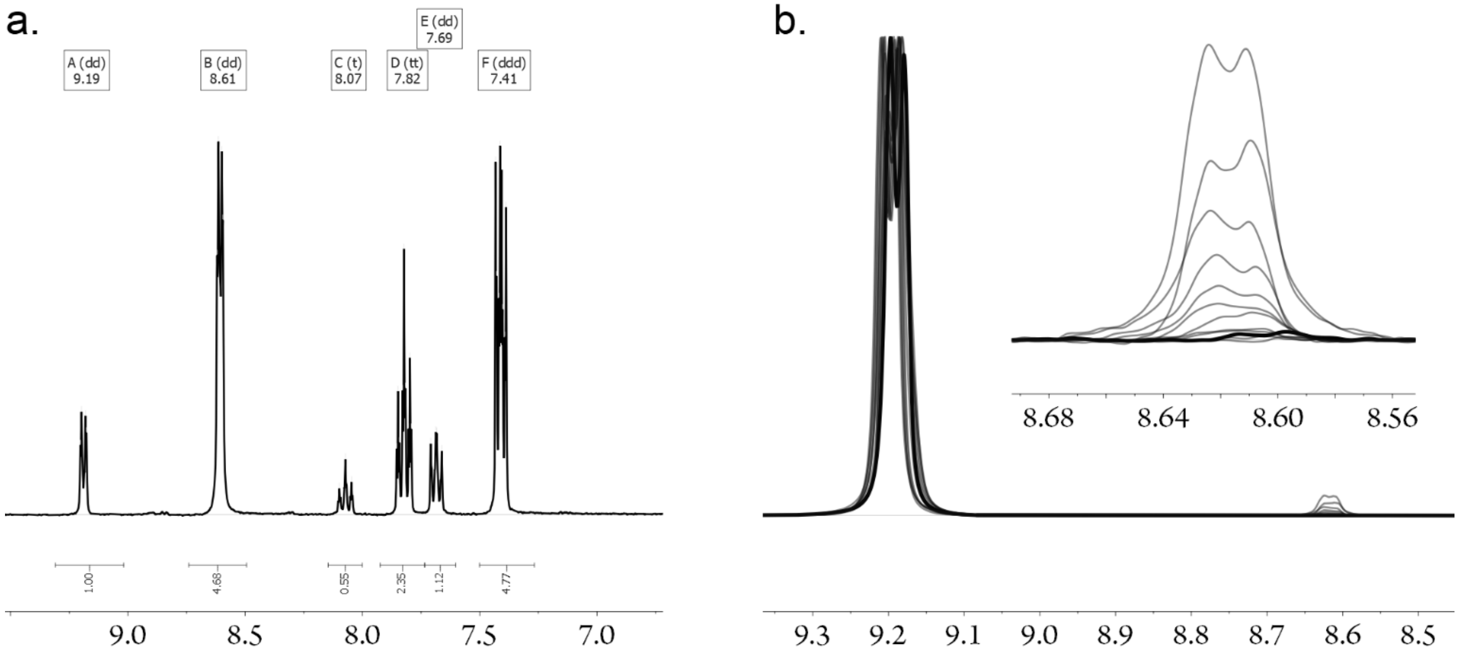

Figure S24. (a) α-pyridyl region ^{1}H-NMR spectrum (300 MHz) of **M**(**py**)$_2$(OTf^-)$_2$ complexes formed with excess pyridine (167.3 mM) in $MeNO_2$-d_3. (b) VT-NMR spectra (normalized and overlaid) used to monitor the dissociation of **py** from **M**(**py**)$_2$ at temperatures between 300–355 K in 5 K increments with 10 minutes between each sample measurement. Complexes were formed by the complexation of **M**(NO_3^-)$_2$ ([**M**] = 17.3 mM) with excess **py** ([**py**] = 167.3 mM). Inset shown to magnify evolved **py** peak arising from dissociation.

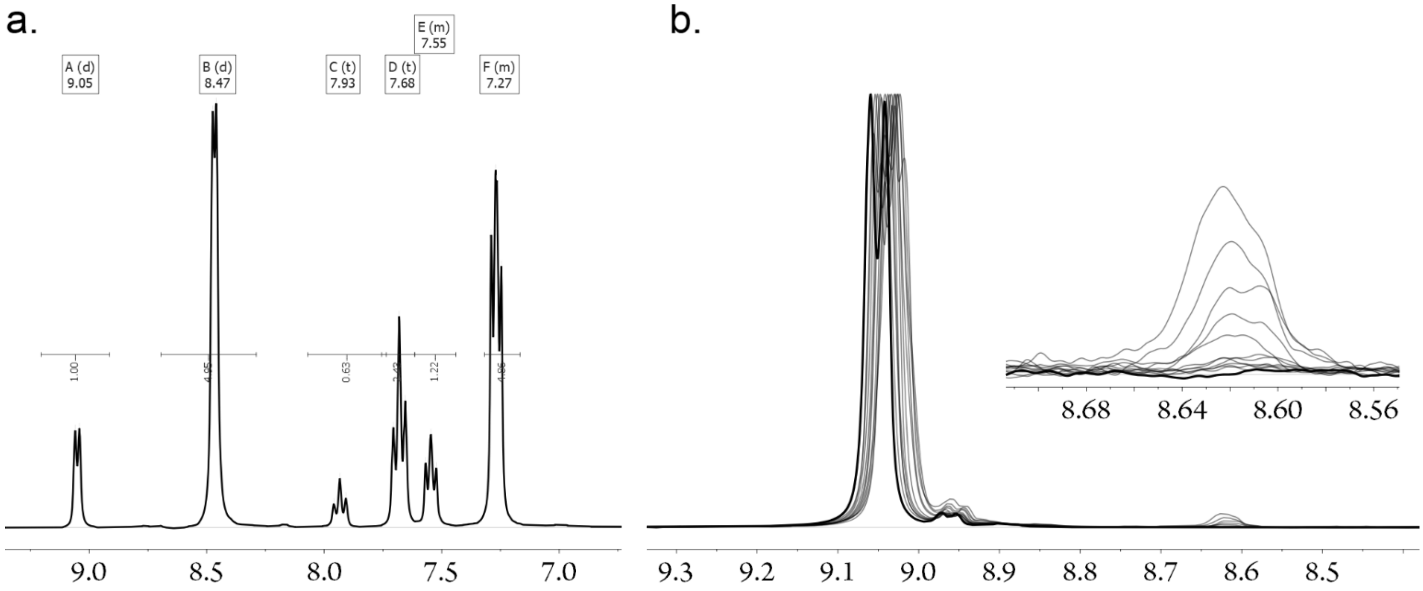

Figure S25. (a) α-pyridyl region ^{1}H-NMR spectrum (300 MHz) of **M**(**py**)$_2$(IO_4^-)$_2$ complexes formed with excess pyridine (167.3 mM) in $MeNO_2$-d_3. (b) VT-NMR spectra (normalized and overlaid) used to monitor the dissociation of **py** from **M**(**py**)$_2$ at temperatures between 300–355 K in 5 K increments with 10 minutes between each sample measurement. Complexes were formed by the complexation of **M**(NO_3^-)$_2$ ([**M**] = 17.3 mM) with excess **py** ([**py**] = 167.3 mM). Inset shown to magnify evolved **py** peak arising from dissociation.

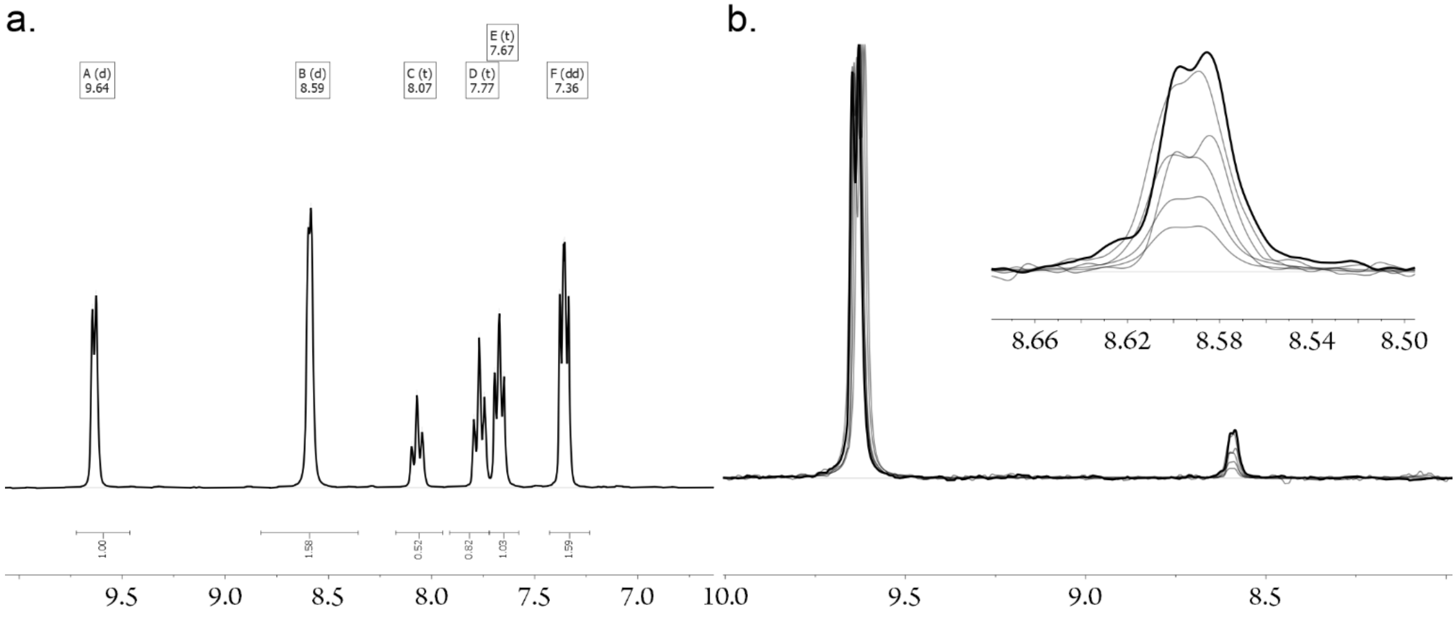

Figure S26. (a) α-pyridyl region ^{1}H-NMR spectrum (300 MHz) of **M**(**py**)$_2$(NO_3^-)$_2$ complexes formed with excess pyridine (167.3 mM) in Acetone-d_6. (b) VT-NMR spectra (normalized and overlaid) used to monitor the dissociation of **py** from **M**(**py**)$_2$ at temperatures between 300–325 K in 5 K increments with 10 minutes between each sample measurement. Complexes were formed by the complexation of **M**(NO_3^-)$_2$ ([**M**] = 17.3 mM) with excess **py** ([**py**] = 167.3 mM). Inset shown to magnify evolved **py** peak arising from dissociation. Inset shown to magnify evolved **py** peak arising from dissociation.

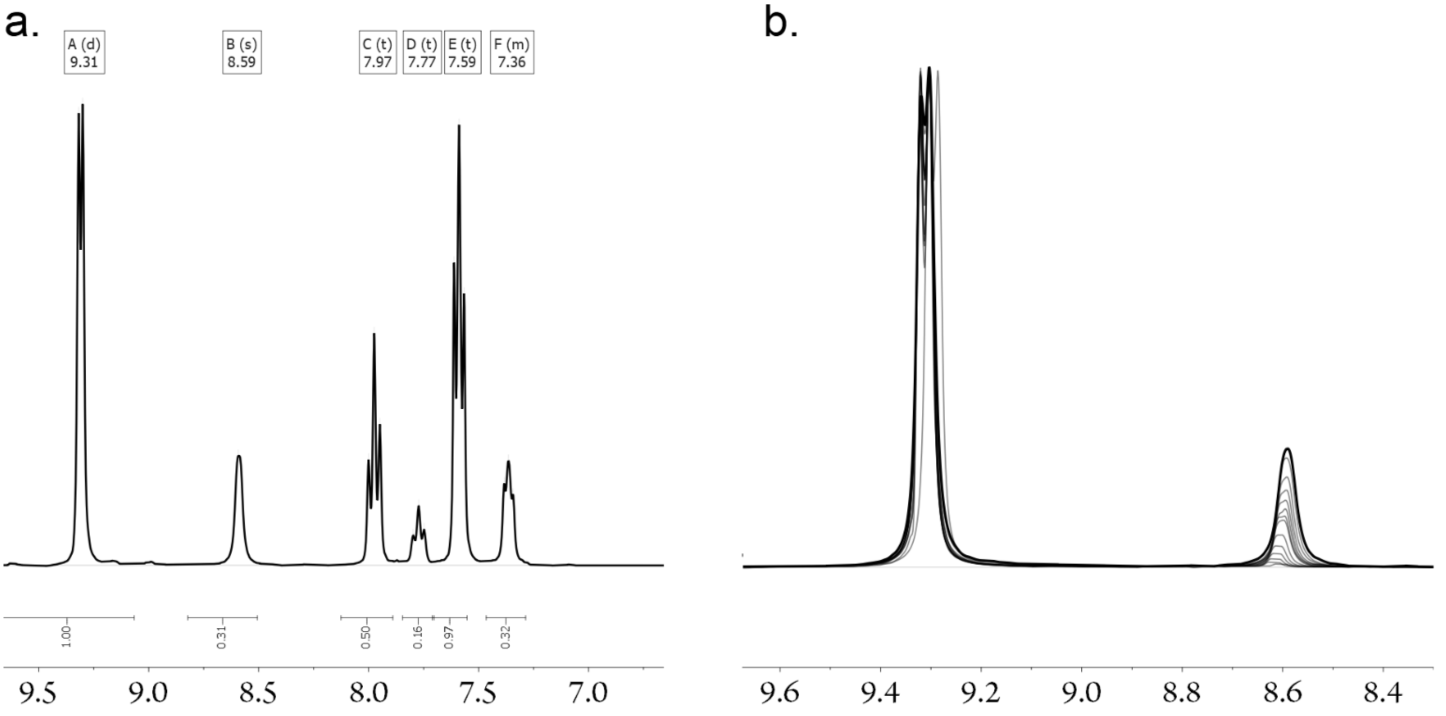

Figure S27. (a) α-pyridyl region ^{1}H-NMR spectrum (300 MHz) of **M**(**py**)$_2$(NO_3^-)$_2$ complexes formed with excess pyridine (167.3 mM) in MeCN-d_3. (b) VT-NMR spectra (normalized and overlaid) used to monitor the dissociation of **py** from **M**(**py**)$_2$ at temperatures between 300.0–347.5 K in 5.0 or 2.5 K increments with 10 minutes between each sample measurement. Complexes were formed by the complexation of **M**(NO_3^-)$_2$ ([**M**] = 17.3 mM) with excess **py** ([**py**] = 40.1 mM).

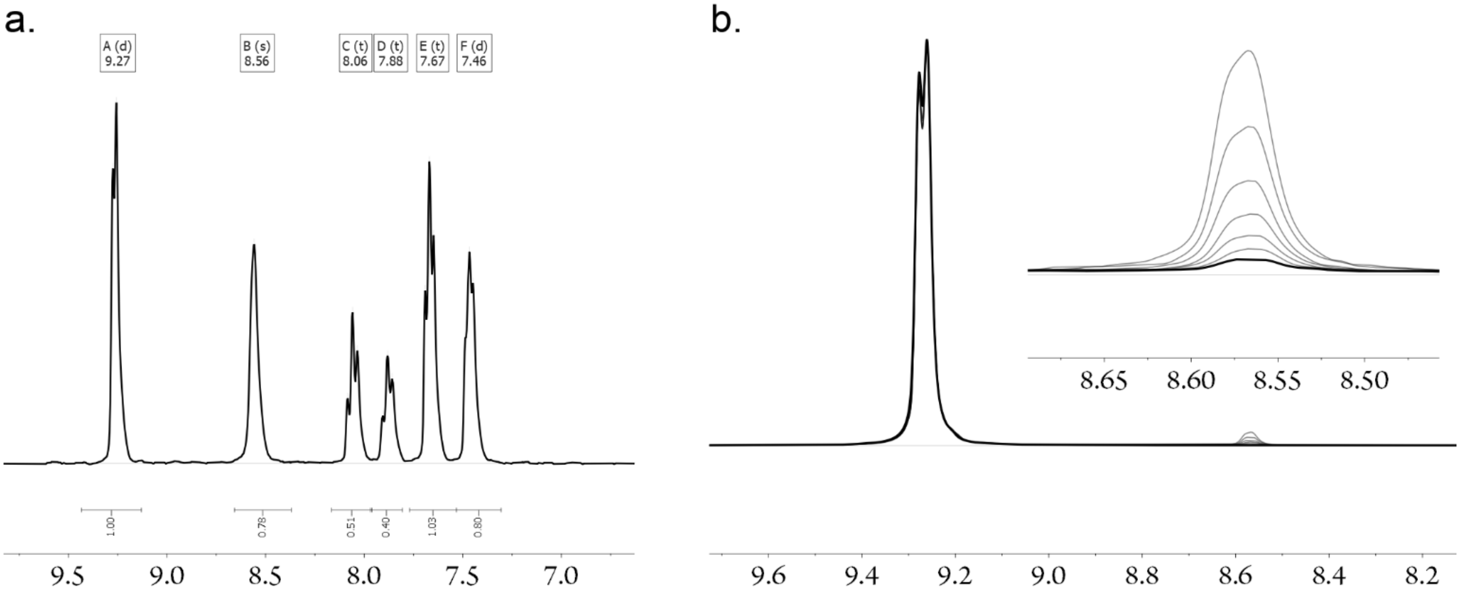

Figure S28. (a) α-pyridyl region ^{1}H-NMR spectrum (300 MHz) of **M**(**py**)$_2$(NO_3^-)$_2$ complexes formed with excess pyridine (167.3 mM) in MeOH-d_4. (b) VT-NMR spectra (normalized and overlaid) used to monitor the dissociation of **py** from **M**(**py**)$_2$ at temperatures between 300–355 K in 5 K increments with 10 minutes between each sample measurement. Complexes were formed by the complexation of **M**(NO_3^-)$_2$ ([**M**] = 17.3 mM) with excess **py** ([**py**] = 48.1 mM). Inset shown to magnify evolved **py** peak arising from dissociation.

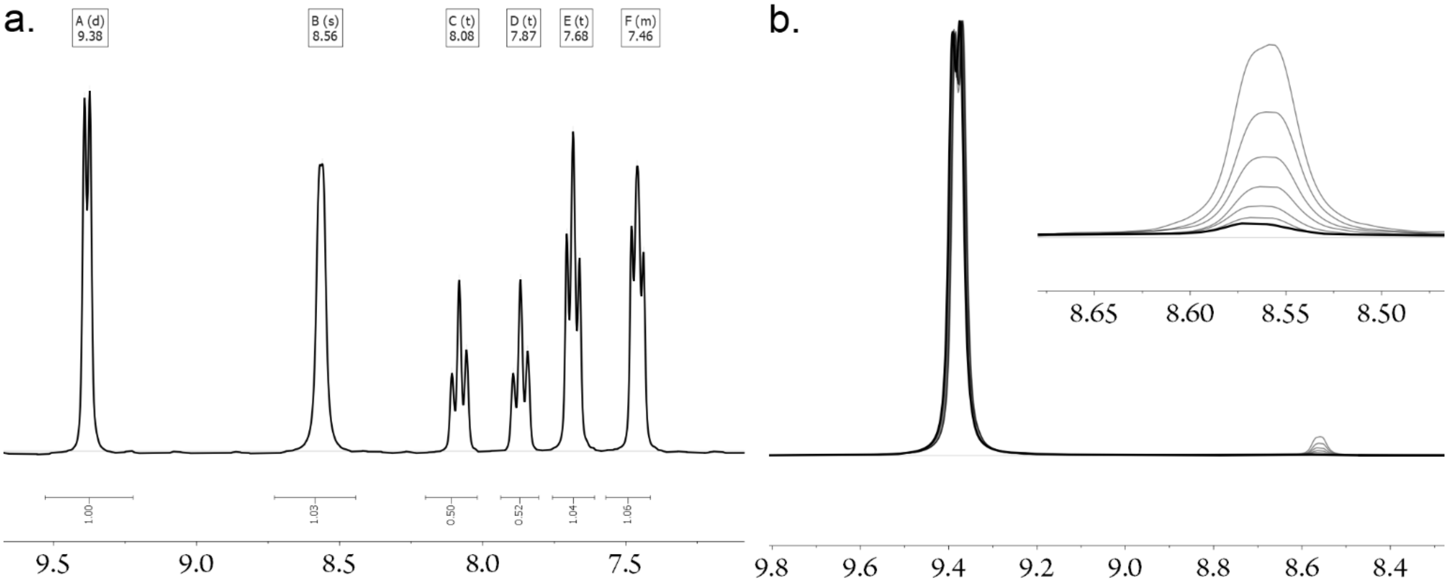

Figure S29. (a) α-pyridyl region ^{1}H-NMR spectrum (300 MHz) of **M**(**py**)$_2$(NO_3^-)$_2$ complexes formed with excess pyridine (167.3 mM) in EtOH-d_6. (b) VT-NMR spectra (normalized and overlaid) used to monitor the dissociation of **py** from **M**(**py**)$_2$ at temperatures between 300–355 K in 5 K increments with 10 minutes between each sample measurement. Complexes were formed by the complexation of **M**(NO_3^-)$_2$ ([**M**] = 17.3 mM) with excess **py** ([**py**] = 52.9 mM). Inset shown to magnify evolved **py** peak arising from dissociation.

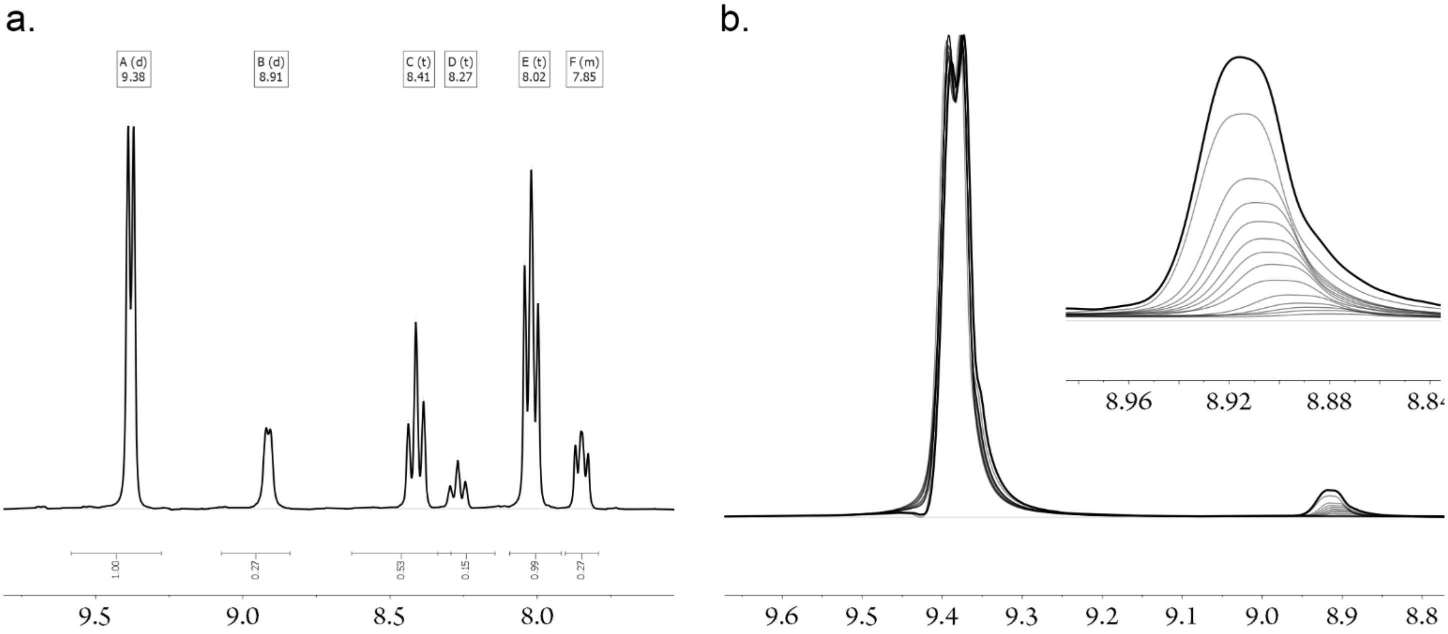

Figure S30. (a) α-pyridyl region ^{1}H-NMR spectrum (300 MHz) of **M**(**py**)$_2$(NO_3^-)$_2$ complexes formed with excess pyridine (167.3 mM) in Water-d_2 (i.e., D_2O). (b) VT-NMR spectra (normalized and overlaid) used to monitor the dissociation of **py** from **M**(**py**)$_2$ at temperatures between 300–365 K in 5 K increments with 10 minutes between each sample measurement. Complexes were formed by the complexation of **M**(NO_3^-)$_2$ ([**M**] = 17.3 mM) with excess **py** ([**py**] = 40.8 mM). Inset shown to magnify evolved **py** peak arising from dissociation.

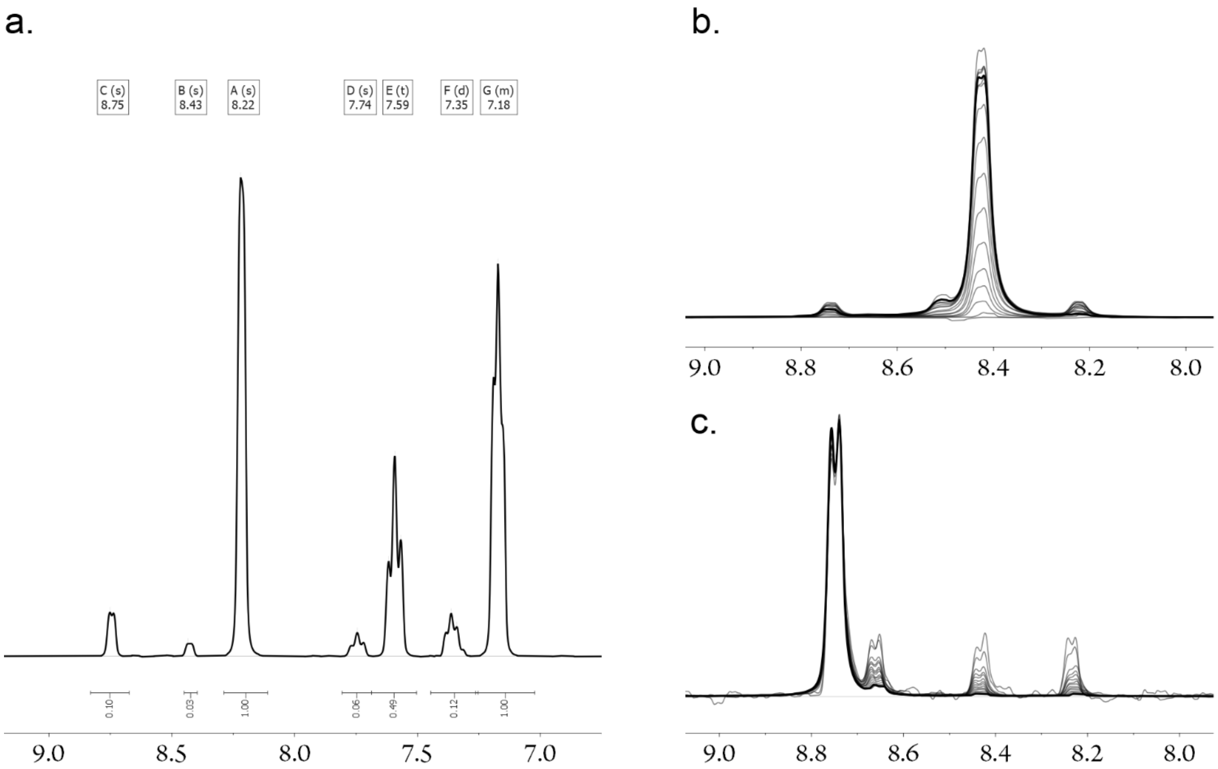

Figure S31. (a) α-pyridyl region ^{1}H-NMR spectrum (300 MHz) of **M**(**py**)$_2$(NO_3^-)$_2$ complexes formed with excess pyridine (167.3 mM) in neat 2,2,2 trifluoroethanol (with DMSO-d_6 used as reference in a coaxial insert). (b) VT-NMR spectra (overlaid) used to monitor the dissociation of **py** from **M**(**py**) at temperatures between 300–355 K in 2.5 K increments with 10 minutes between each sample measurement. (c) VT-NMR spectra (normalized and overlaid) used to monitor the dissociation of **py** from **M**(**py**)$_2$ at temperatures between 300–355 K in 5 K increments with 10 minutes between each sample measurement.

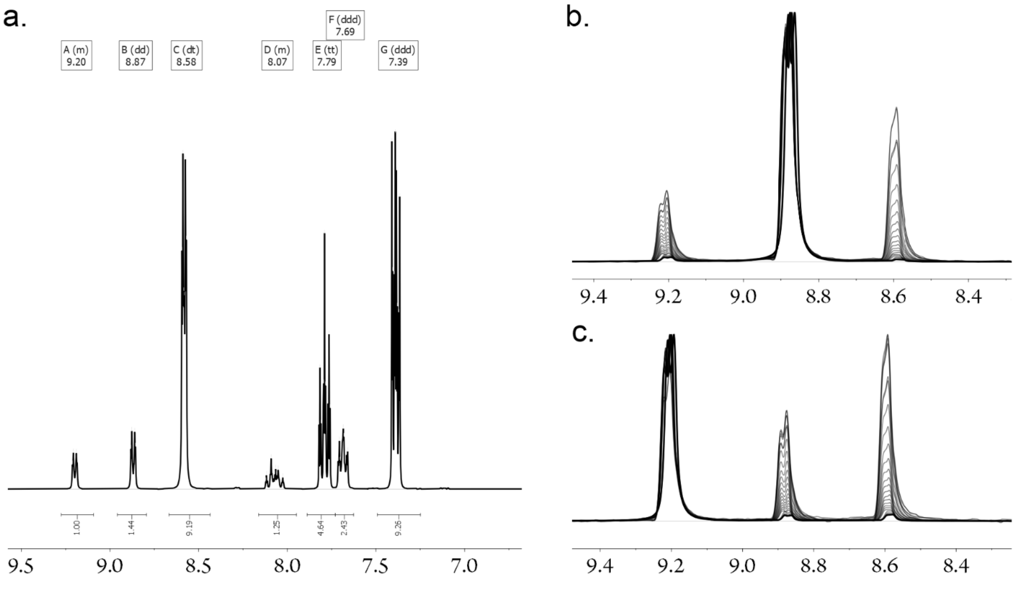

Figure S32. (a) α-pyridyl region ^{1}H-NMR spectrum (300 MHz) of **M**(**py**)$_2$(NO_3^-)$_2$ complexes formed with excess **py** (167.3 mM) in DMSO-d_6 with additive tetra(*n*-butyl)ammonium chloride added (8.2 mM). (b) VT-NMR spectra (normalized and overlaid) used to monitor the dissociation of **py** from **M**(**py**) at temperatures between 300–355 K in 2.5 K increments with 10 minutes between each sample measurement. (c) VT-NMR spectra (normalized and overlaid) used to monitor the dissociation of **py** from **M**(**py**)$_2$ at temperatures between 300–355 K in 5 K increments with 10 minutes between each sample measurement.

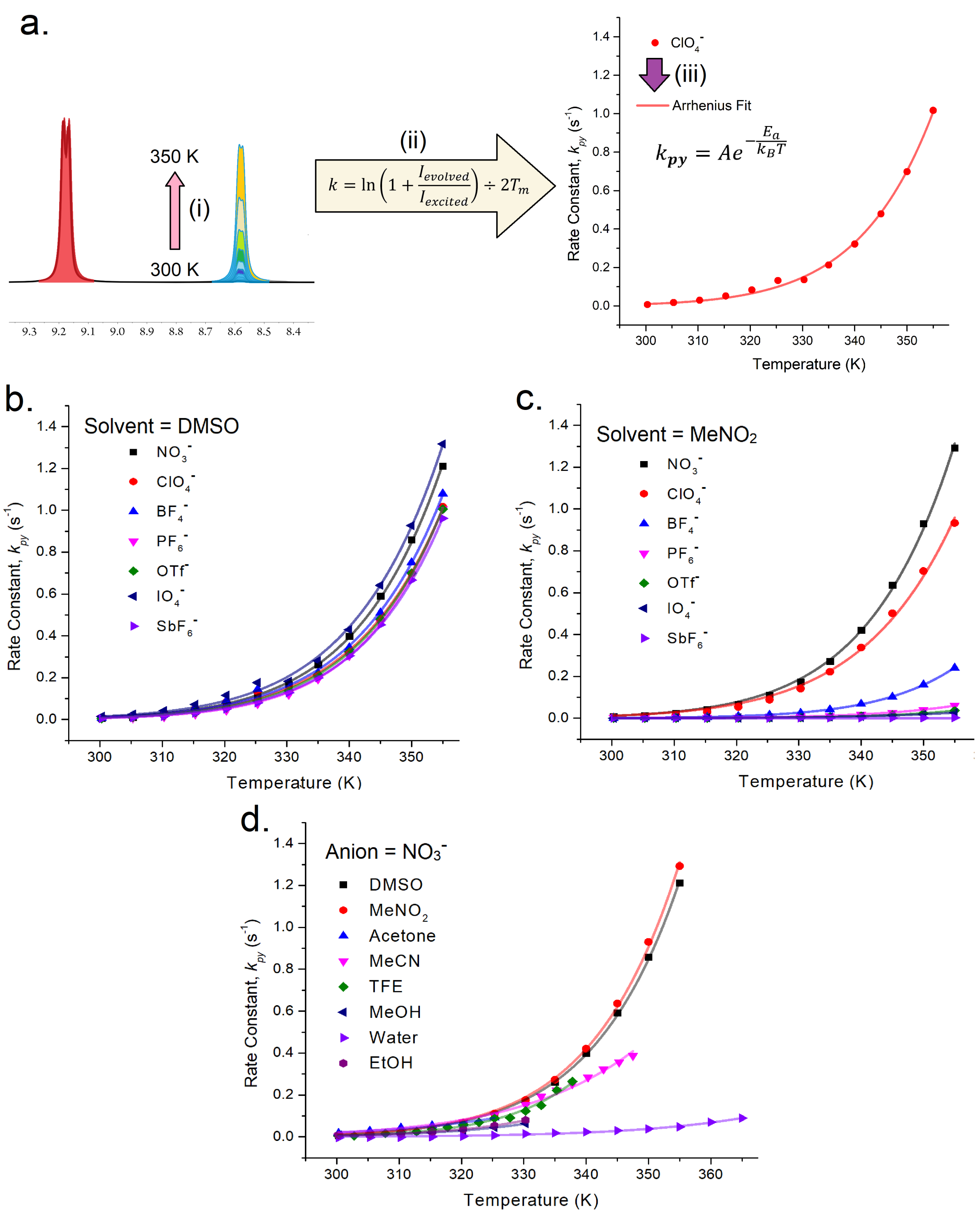

Figure S33. Rate constants computed from EXSY spectra for **py** dissociation (k_{py}) as a function of temperature with changing reaction conditions. (a) A schematic representation of the data analysis scheme: i) varying temperature leads to a relative increase in the evolved peak observed by EXSY, ii) the ratios of the excited and evolved peaks are converted to rate constants (k_{py}) for each temperature a with Equation 2,[7] iii) these rate constants are directly fit as a function of temperature with Arrhenius equation (Equation 3) to determine E_a. (b) Varying anion with DMSO solvent (Figures S13b–S19b), (c) Varying anion with $MeNO_2$ solvent (Figures S20b–S26b), (d) Varying solvent with NO_3^- anions (Figures S13b, S20b, S27b–S30b, S30c). The resulting data are tabulated below as Tables S1 and S2.

Table S1. Activation energies for pyridyl ligand substitution observed for $M(py)_2$ complexes with varying anions.

Anion	E_a (kcal mol^{-1})<br>Solvent = DMSO	E_a (kcal mol^{-1})<br>Solvent = $MeNO_2$
NO_3^-	12.08 ± 0.59	11.61 ± 0.59
ClO_4^-	12.14 ± 0.29	11.88 ± 0.21
BF_4^-	12.12 ± 0.55	12.60 ± 0.64
PF_6^-	12.08 ± 0.31	13.79 ± 0.21
OTf^-	12.24 ± 0.54	14.16 ± 0.36
IO_4^-	12.27 ± 0.50	14.95 ± 0.77
SbF_6^-	12.29 ± 0.41	16.27 ± 0.43

Table S2. Activation energies for pyridyl ligand substitution observed for $M(py)_2(NO_3^-)_2$ complexes with varying solvent.

Solvent	E_a (kcal mol^{-1})<br>Anion = NO_3^-
$MeNO_2$	11.61 ± 0.59
Acetone	11.14 ± 0.84
MeCN	12.20 ± 0.51
DMSO	12.08 ± 0.59
TFE[1]	15.17 ± 0.70
Water (D_2O)	16.94 ± 0.43
MeOH	19.57 ± 0.85
EtOH	21.26 ± 1.40

[1]- E_A estimate is based on overall rate, see Figure 7 and S34 for further details of involved microscopic processes.

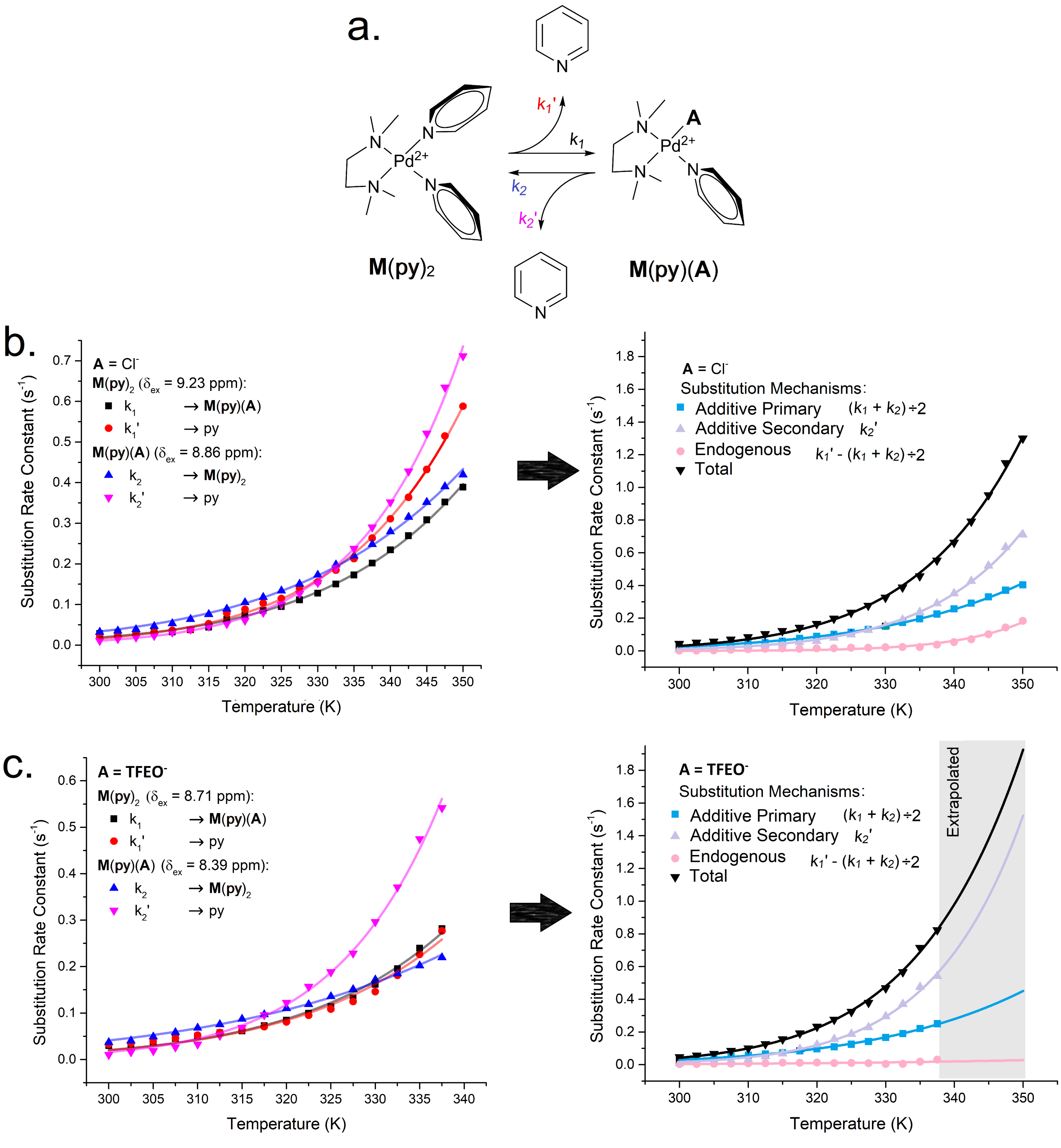

Figure S34. (a) Scheme of reactions measured using VT–EXSY. (b) Measured rate constants (k_1, k_1', k_2, and k_2') and the interpreted rate of pyridyl-ligand substitution arising from each mechanism for complexes formed from **M** (17.3 mM) and **py** (167.3 mM) with additive tetra(*n*-butyl)ammonium chloride (8.2 mM, **A** = Cl^-) in DMSO, see Figure S31 for spectra. (c) The same measured values, but for complexes formed from **M** (17.3 mM) and **py** (167.3 mM) in neat TFE (**A** = **TFEO**$^-$) without additional additives, see Figure S32 for spectra.

Models were constructed for each anion structure and optimized using Gaussian 16 (b3lyp/def2tzv), electrostatic potential (ESP) volumes were calculated using the cubegen utility included with GaussView 6.[2] The zero-potential surface—where there is no net electrostatic potential—was rendered using the volume viewer included with UCSF Chimera (see Figure S35).[3] The volume within this surface, V_{esp}, computed using the volume properties analyzer included with UCSF Chimera.

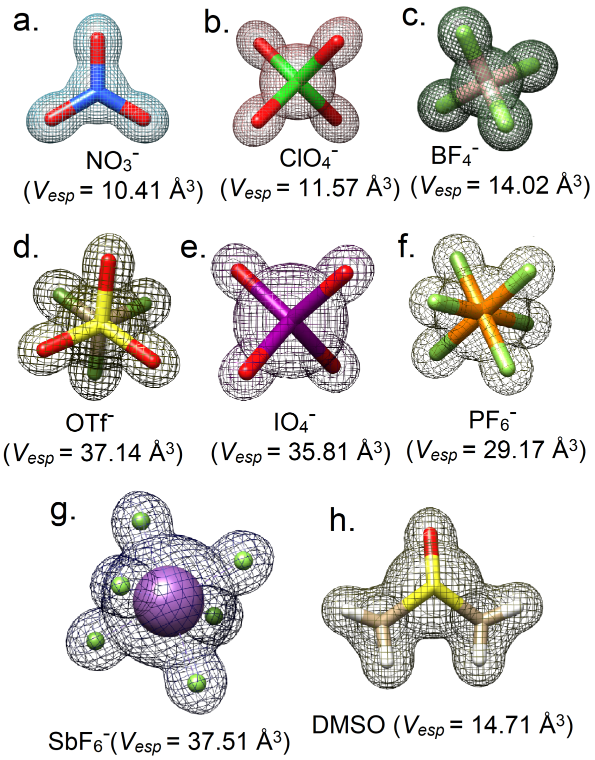

Figure S35. DFT-optimized models of anion (a–g) and solvent (h) molecules rendered with their zero-potential surface, and listed alongside their V_{esp} (i.e., the volume within the shown zero-potential surface).

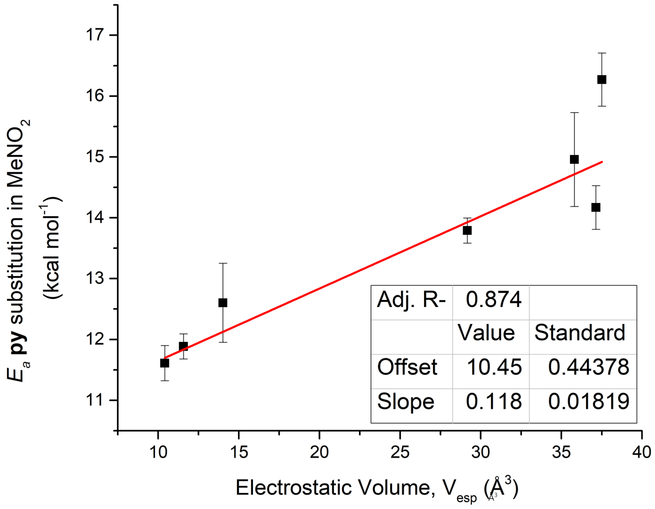

Figure S36. Correlation between DFT-computed V_{esp} (Figures S35) and experimentally determined E_a for **py** substitution in $MeNO_2$ as a non-coordinating solvent (Figure 3).

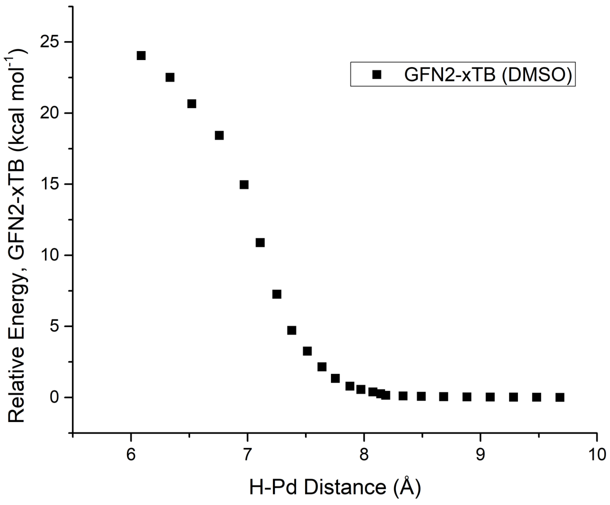

Figure S37. GFN2-xTB[5] simulated approach of H^+ to a pyridyl N-atom of **M**(**py**)$_2$ demonstrating the significant barrier imposed by electrostatic repulsion between H^+ and Pd^{2+}.

J. Molecular dynamics simulations

Parameters were optimized for **M**(**py**)$_2$ complexes previously reported protocol.[4] Quantum mechanical molecular dynamics trajectories were obtained for a model **M**$_2^{lin}$***L***$_3$ oligomer using *xtb* (GFN2-xTB),[5] which were subsequently used for single point DFT calculations using *Gaussian16 Rev C.* at a b3lyp/def2tzv theory level.[2] Parameters were then optimized using *paramfit* to obtain a high-fidelity reproduction of the DFT energies (Figure S3).[6]

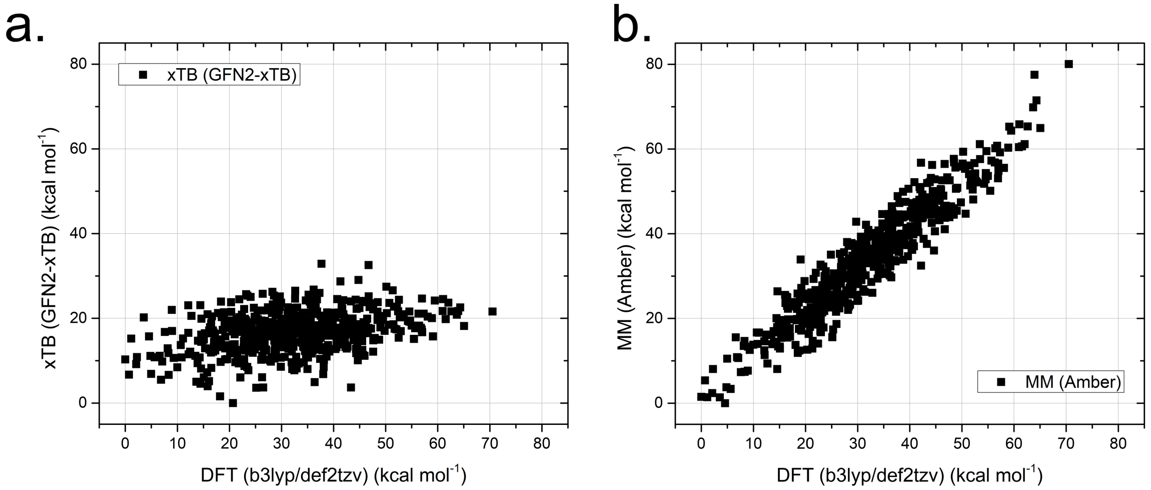

Figure S38. Comparison of DFT single point energies for configurations of the **M**$_2^{lin}$**L**$_3$ oligomer to energies obtained from (a) semi-empirical (GFN2-xTB) and (b) molecular-mechanical (optimized Amber forcefield). Frames were generated from a molecular dynamics simulation using xtb ($n_{configuraiton}$ = *512*).

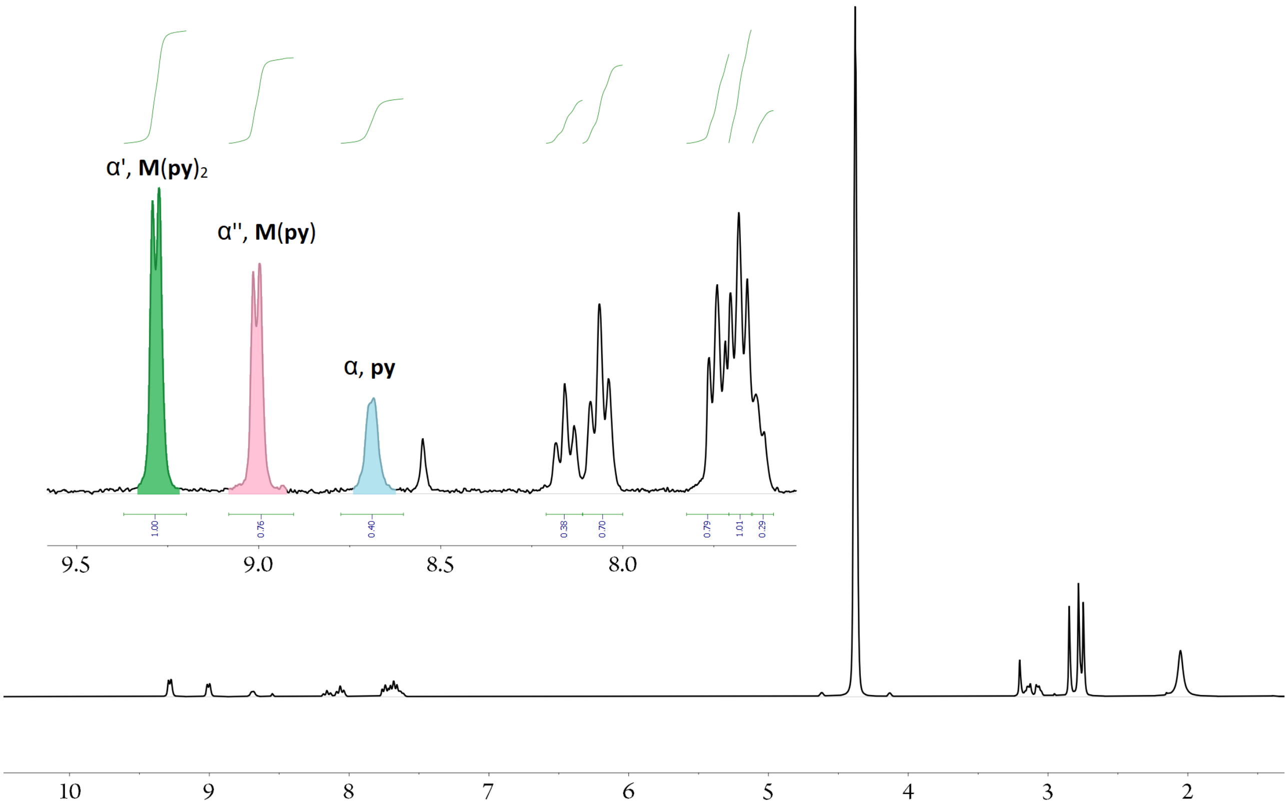

Figure S39.^{1}H-NMR spectrum (300 MHz) of **M**(**py**)$_2$ complexes formed with **py** (58.3 mM) and **M** (17.3 mM) in MeNO$_2$-d_3 at 350K. Peaks are labeled based on species, see Figure 1.

Absorption spectra were obtained using a Shimadzu UV-2600 with an attached temperature controller (C101-E121B). Complexes were formed by a mixture of **M** (17.3 mM) and excess **py** (157.6 mM) in a 2 mm pathlength quartz cuvette (Starna). Spectra were obtained at room temperature (298 K) and multiple elevated temperatures (varies by solvent), with 10 minutes equilibration time between each temperature setpoint. A series of difference spectra were produced by subtraction of the spectrum obtained at 298 K and used for further analysis by linear fitting to obtain a temperature-correlated absorption spectrum (Scheme S2). These were then compared to TDDFT calculations.

Using Gaussian 16,[2] models for each complex (and oppositional complex) were optimized using DFT at a b3lyp/def2tzv level of theory, with conductor-like polarizable continuum model solvation (CPCM) matching the experimental system. These optimized models were then used for TDDFT calculations at the same b3lyp/def2tzv level of theory.

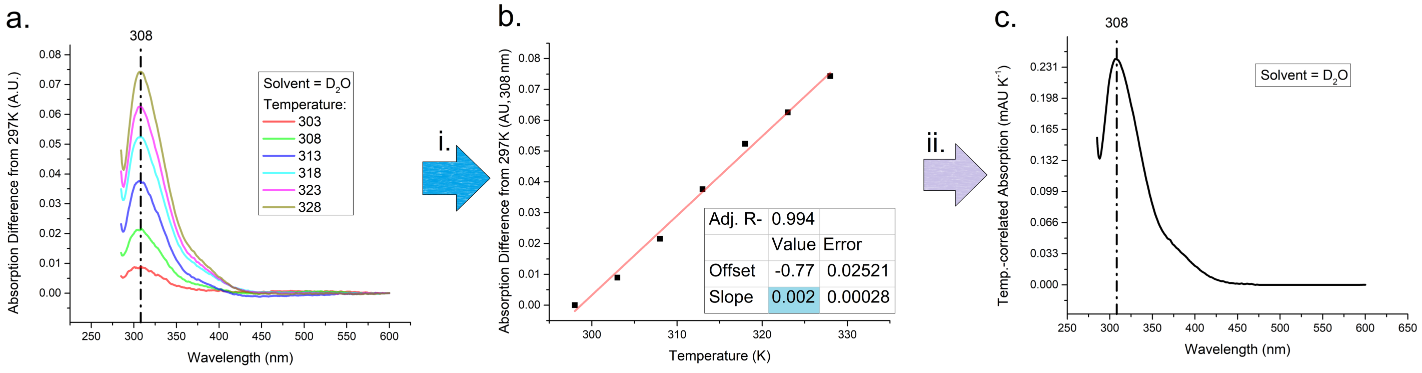

Scheme S2. A series of difference spectra (a) are obtained at 5–10 elevated temperature points as allowed by the sample (due to considerations such as evaporation). The changes in absorption are then considered as a function of temperature (i) and a series of linear fits (b) are conducted for each wavelength. The slope (b, highlighted in blue) is then plotted as a function of wavelength (ii) giving a temperature-correlated absorption spectrum (c). Note, the slope at 308 nm (b, blue, in AU) corresponds to the value of the temperature correlated spectrum (c, in mAU).

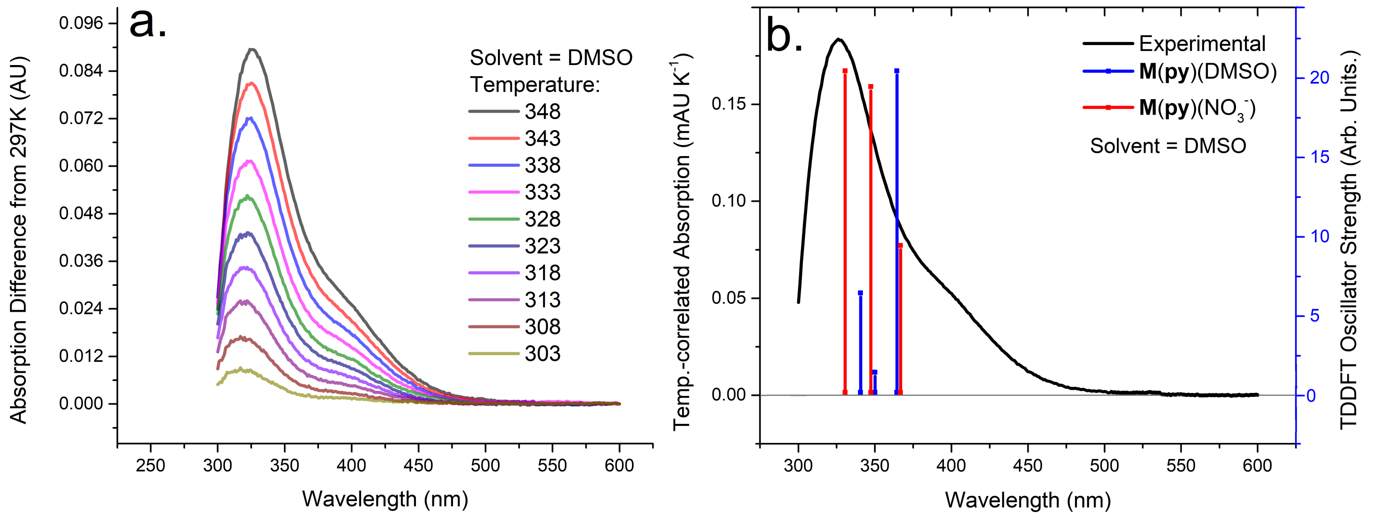

Figure S40. (a) A series of absorption difference spectra over a range of elevated temperatures obtained in DMSO. (b) Plot of the Temperature-correlated spectrum (black trace) compared to the vertical transitions computed for **M**(**py**)(DMSO) (blue) and **M**(**py**)(NO_3^-) (red). DFT and TDDFT calculations were carried out with CPCM(DMSO) solvation.

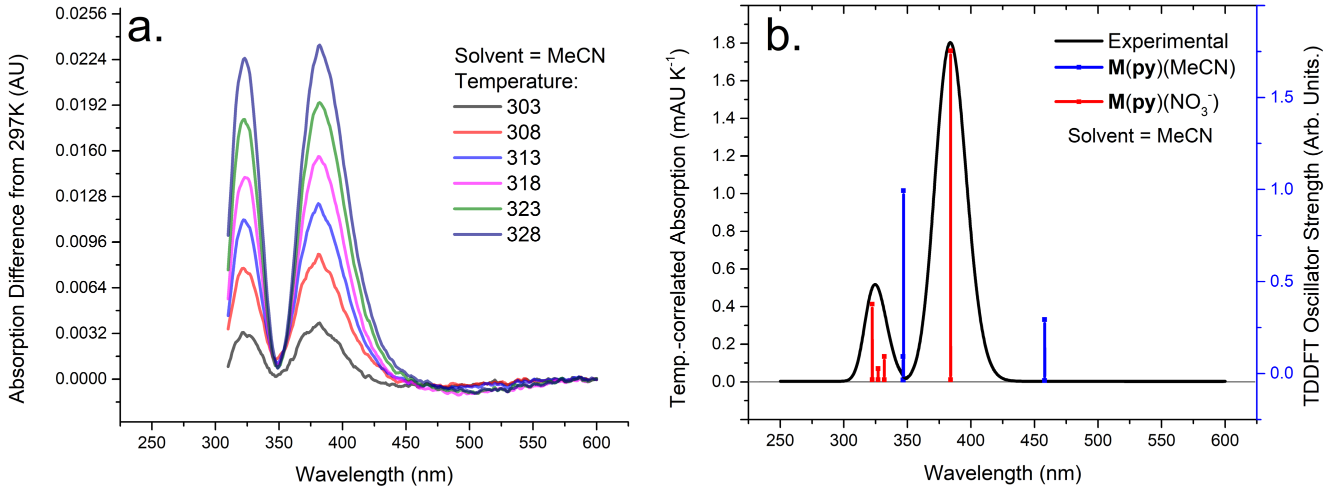

Figure S41. (a) A series of absorption difference spectra over a range of elevated temperatures obtained in MeCN. (b) Plot of the Temperature-correlated spectrum (black trace) compared to the vertical transitions computed for **M**(**py**)(MeCN) (blue) and **M**(**py**)(NO_3^-) (red). DFT and TDDFT calculations were carried out with CPCM(MeCN) solvation.

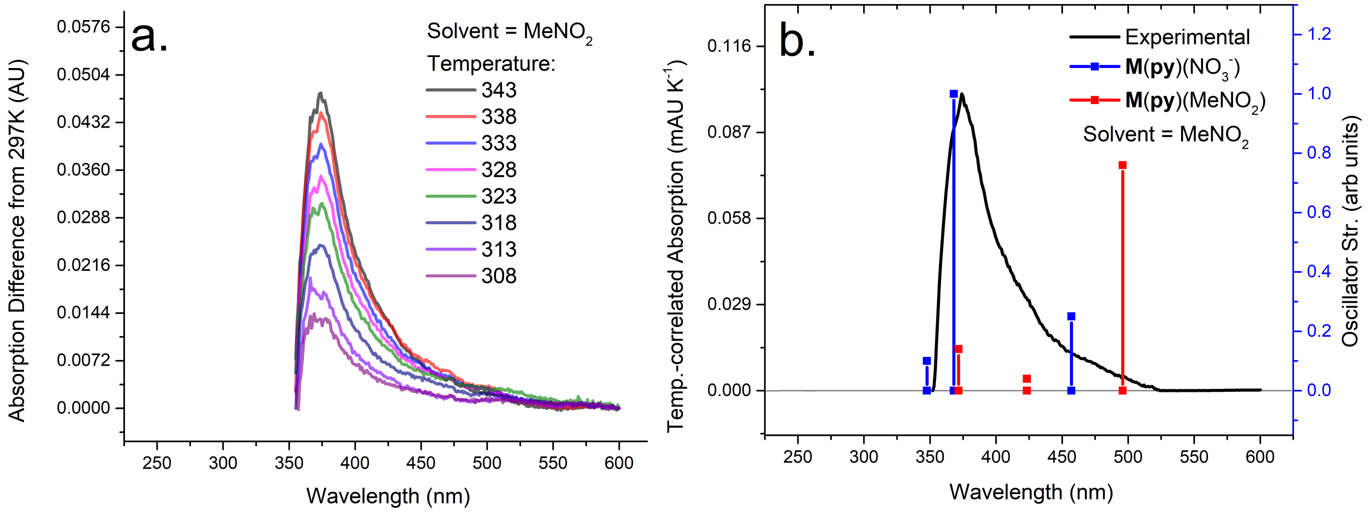

Figure S42. (a) A series of absorption difference spectra over a range of elevated temperatures obtained in DMSO. (b) Plot of the Temperature-correlated spectrum (black trace) compared to the vertical transitions computed for **M**(**py**)(NO_3^-) (blue) and **M**(**py**)($MeNO_2$) (red). DFT and TDDFT calculations were carried out with CPCM($MeNO_2$) solvation.

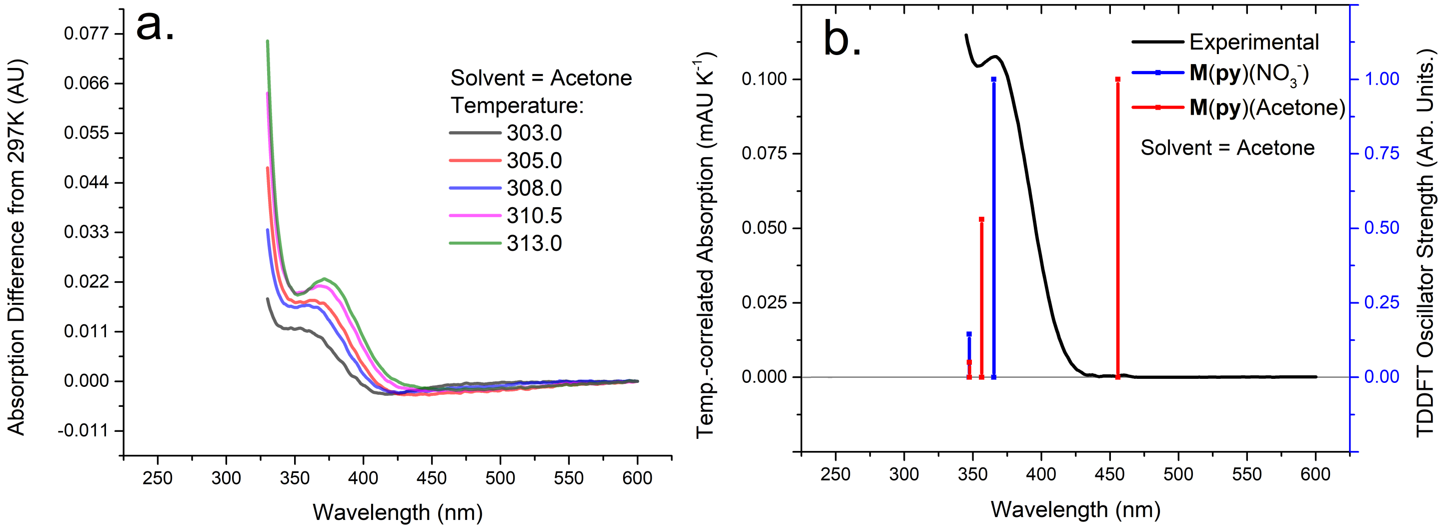

Figure S43. (a) A series of absorption difference spectra over a range of elevated temperatures obtained in acetone. (b) Plot of the temperature-correlated spectrum (black trace) compared to the vertical transitions computed for **M**(**py**)(NO_3^-) (blue) and **M**(**py**)(Acetone) (red). DFT and TDDFT calculations were carried out with CPCM(Acetone) solvation.

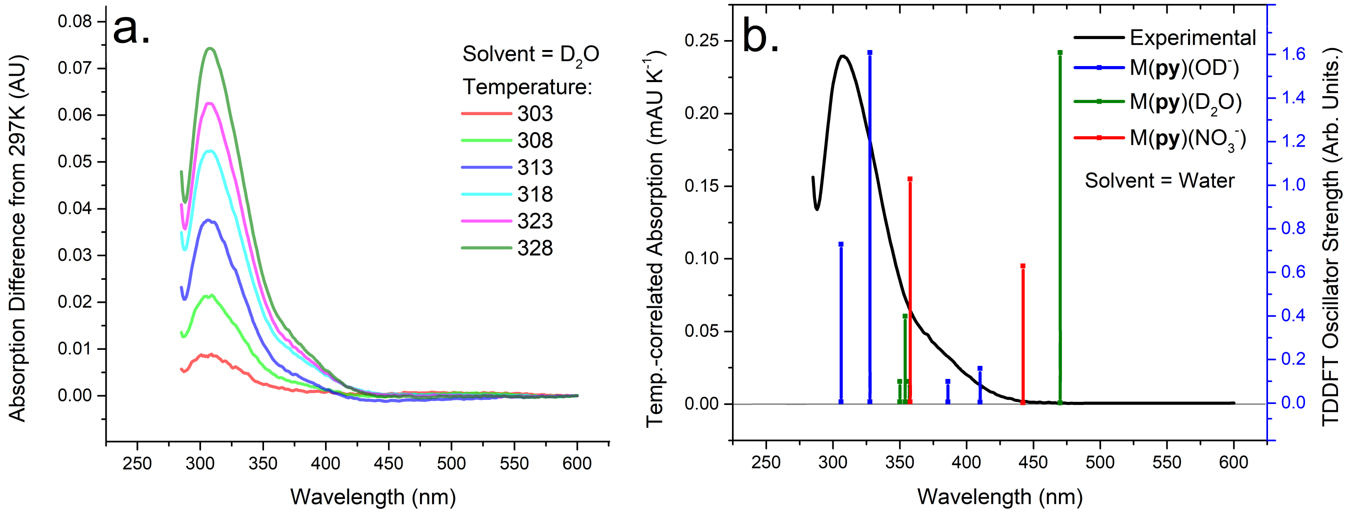

Figure S44. (a) A series of absorption difference spectra over a range of elevated temperatures obtained in D_2O (i.e., water-*d*$_2$). (b) Plot of the temperature-correlated spectrum (black trace) compared to the vertical transitions computed for **M**(**py**)(OD^-) (blue), **M**(**py**)(D_2O) (green), and **M**(**py**)(NO_3^-) (red). DFT and TDDFT calculations were carried out with CPCM(D_2O) solvation.

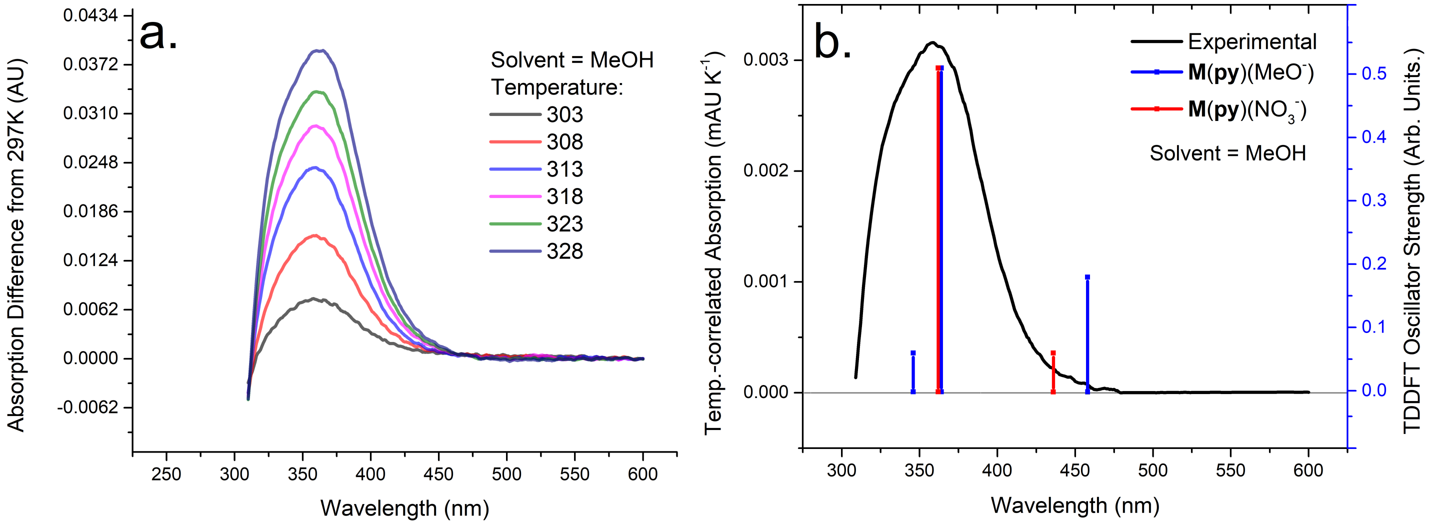

Figure S45. (a) A series of absorption difference spectra over a range of elevated temperatures obtained in MeOH. (b) Plot of the temperature-correlated spectrum (black trace) compared to the vertical transitions computed for **M**(**py**)(MeO^-) (blue) and **M**(**py**)(NO_3^-) (red). DFT and TDDFT calculations were carried out with CPCM(MeOH) solvation.

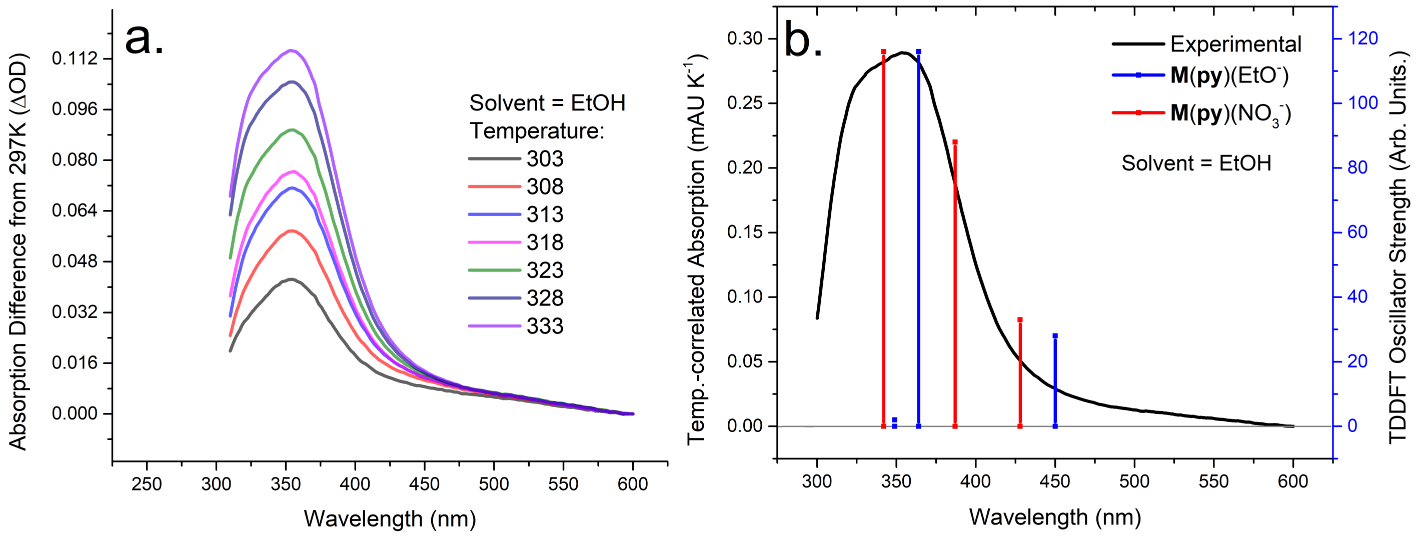

Figure S46. (a) A series of absorption difference spectra over a range of elevated temperatures obtained in EtOH. (b) Plot of the temperature-correlated spectrum (black trace) compared to the vertical transitions computed for **M**(**py**)(EtO^-) (blue) and **M**(**py**)(NO_3^-) (red). DFT and TDDFT calculations were carried out with CPCM(EtOH) solvation.

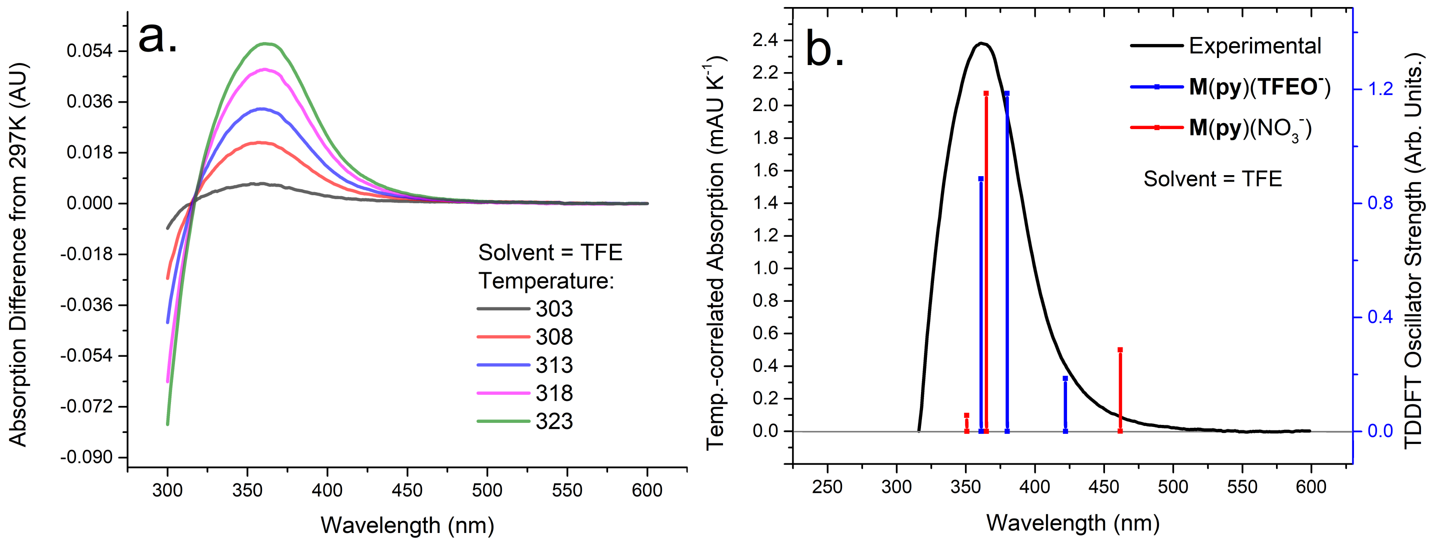

Figure S47. (a) A series of absorption difference spectra over a range of elevated temperatures obtained in TFE. (b) Plot of the temperature-correlated spectrum (black trace) compared to the vertical transitions computed for **M**(**py**)(**TFEO**$^-$) (blue) and **M**(**py**)(NO_3^-) (red). DFT and TDDFT calculations were carried out with CPCM(TFE) solvation.

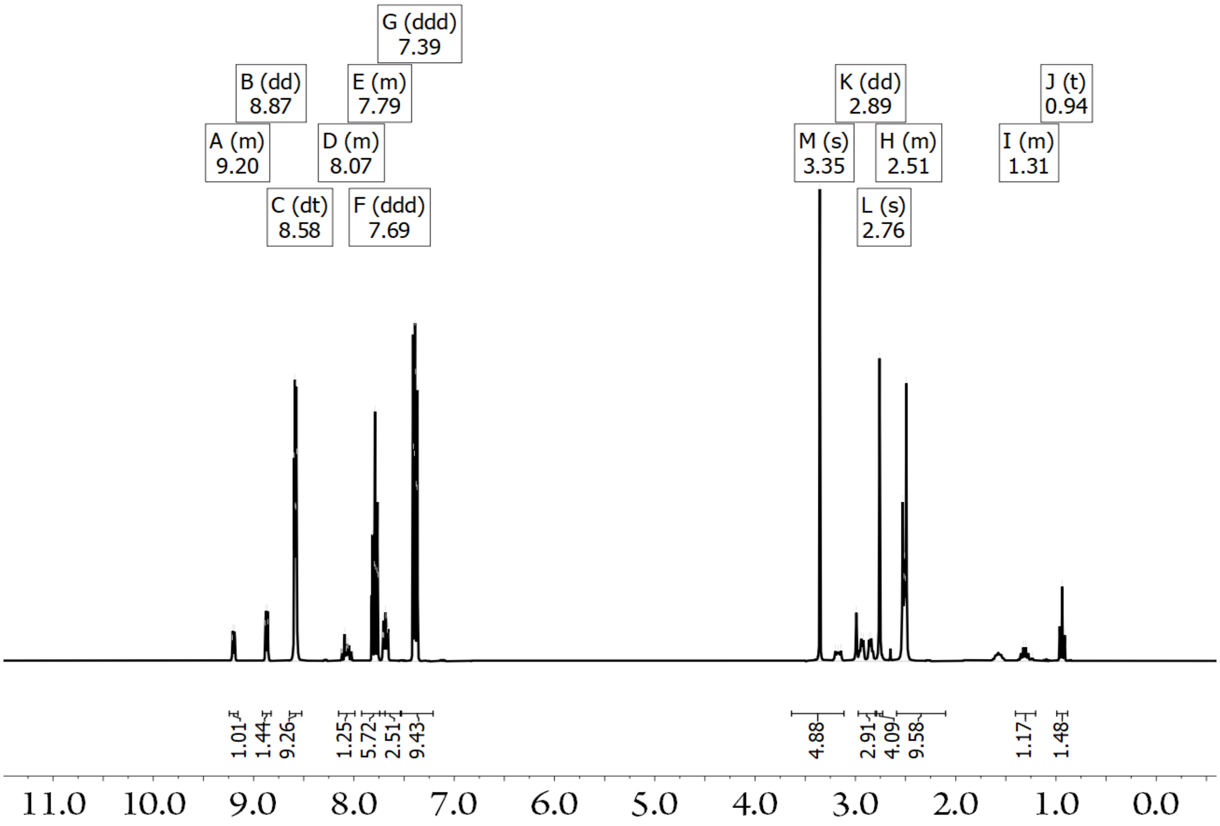

Figure S48.^{1}H-NMR spectrum (300 MHz) of **M**(**py**)$_2$ complexes formed with **py** (58.3 mM) and **M** (17.3 mM), with additive tetra(*n*-butyl)ammonium chloride (8.2 mM) in DMSO-d_6.

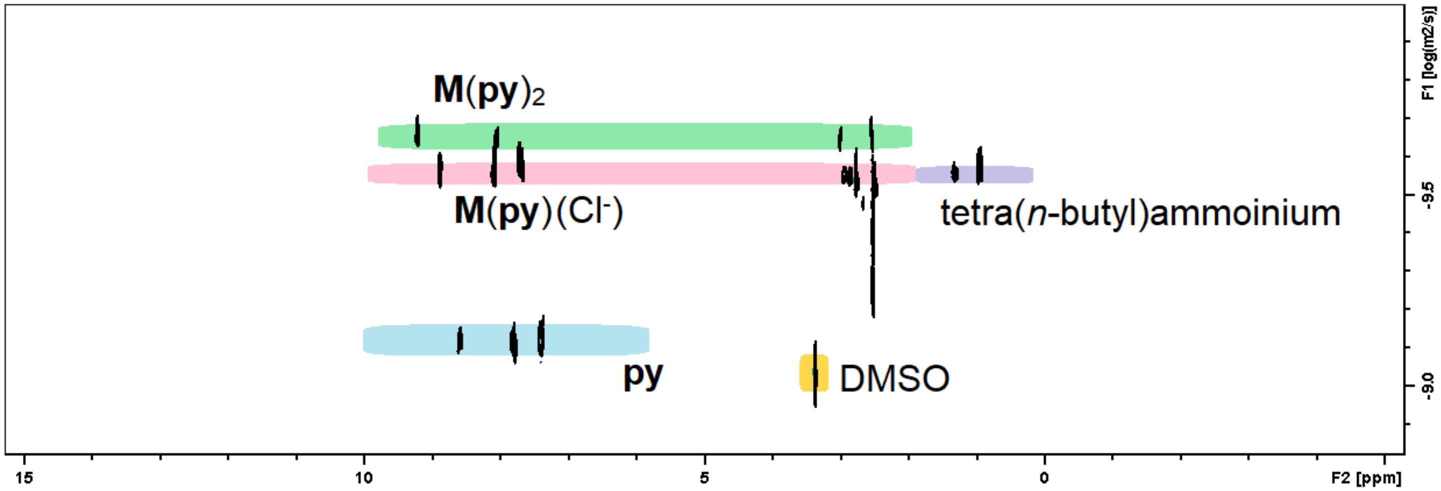

Figure S49. Diffusogram of **M**(**py**)$_2$ (green), **M**(**py**)(Cl$^-$) (pink), **py** (blue), and tetra(*n*-butyl)ammonium computed from the variable gradient pulse sequence (300 MHz, DMSO-d_6, 298 K, Δt = 100 ms, δ = 1 ms, 19 points, ledbpgp2s) using the dosy2d processing program in Topspin 4.0.9.

N. ^{1}H- and ^{19}F-NMR characterization of **M**(**py**)(**TFEO⁻**)

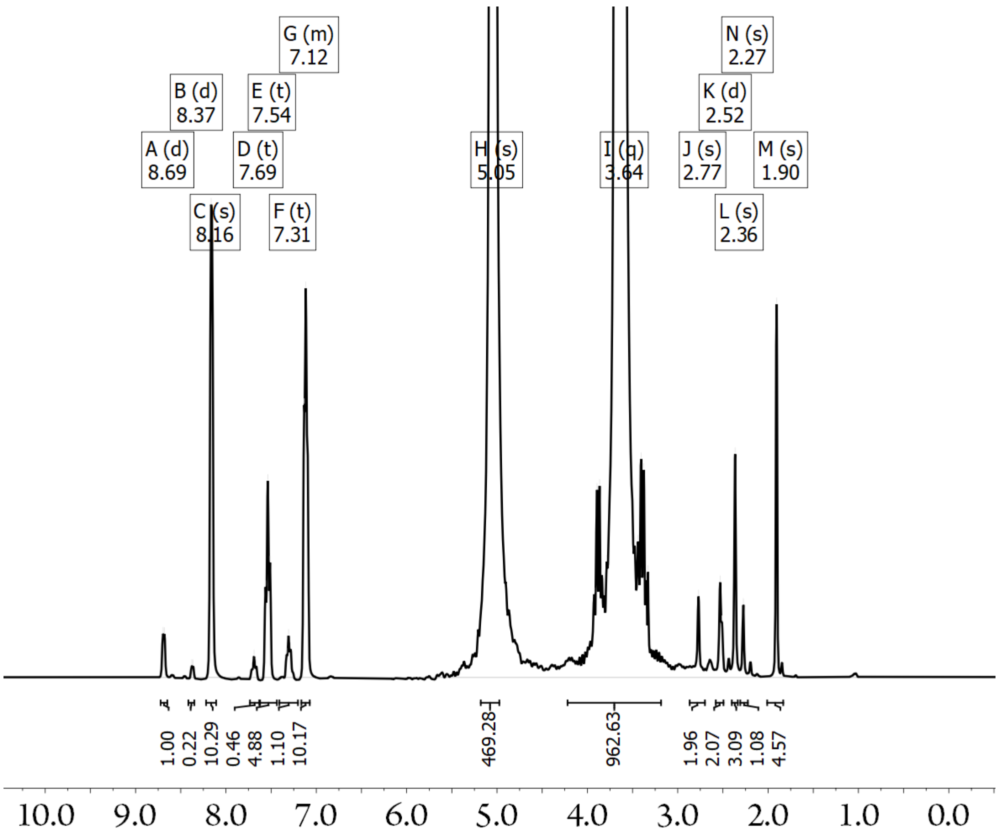

Figure S50. ^{1}H-NMR spectrum (300 MHz) of **M**(**py**)$_2$ complexes formed with **py** (58.3 mM) and **M** (17.3 mM) in pure TFE (peaks H and Q). These spectra were referenced to a coaxial insert tube containing DMSO-d_6.

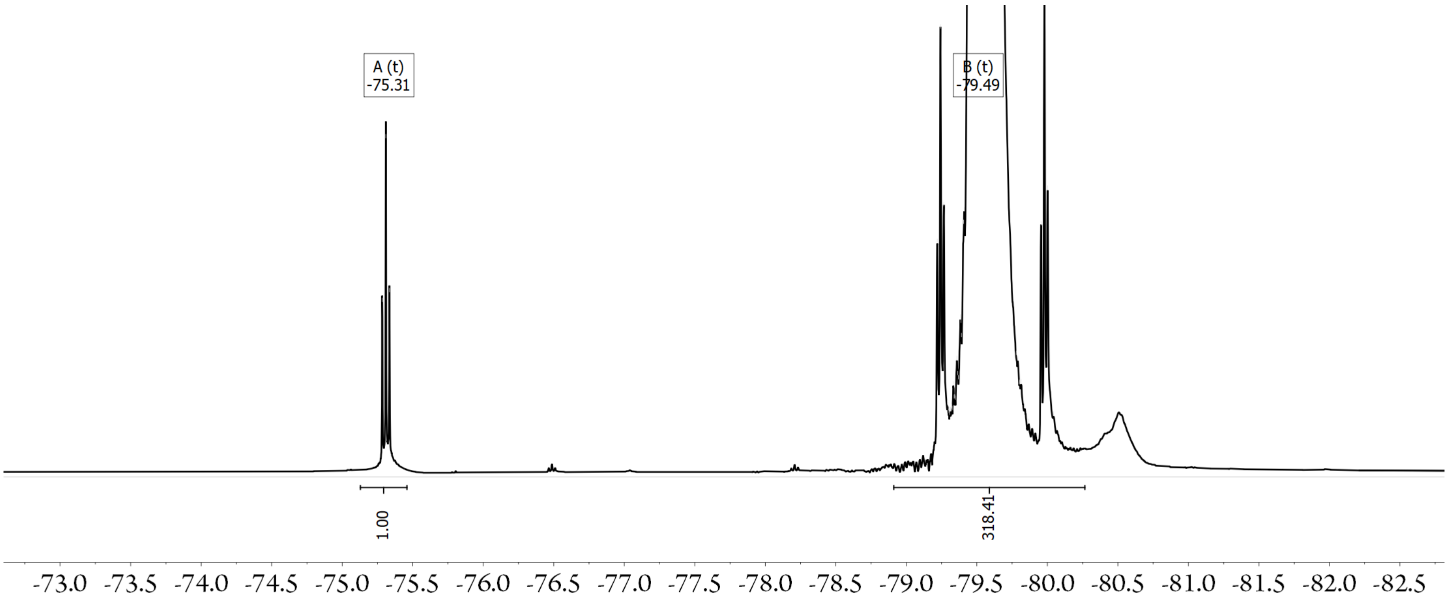

Figure S51. ^{19}F-NMR spectrum (376.3 MHz) of **M**(**py**)$_2$ complexes formed with **py** (58.3 mM) and **M** (17.3 mM) in pure TFE. Peak A corresponds to **M**(**py**)(**TFEO⁻**) (δ = -75.31 ppm) whereas B is the solvent TFE signal. These spectra were referenced to a coaxial insert tube containing DMSO-d_6.

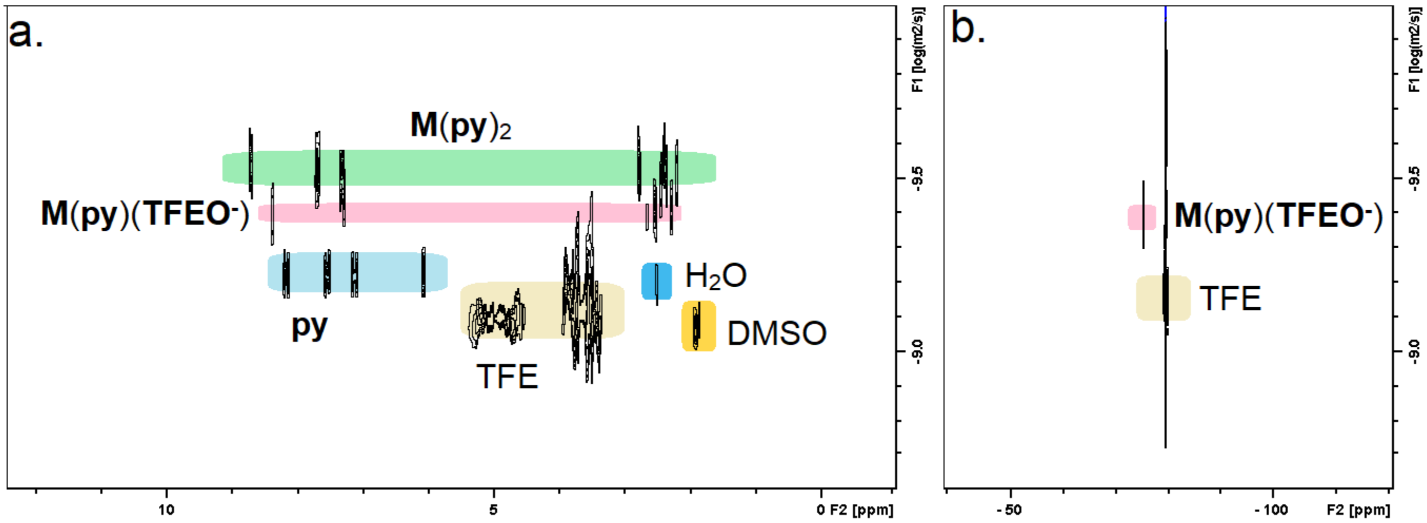

Figure S52. Diffusograms of **M**(**py**)$_2$ (green), **M**(**py**)(**TFEO**$^-$) (pink), and **py** (blue) obtained for both (a) ^{1}H-NMR (400 MHz) and ^{19}F-NMR (376.3 MHz) signals. Both measurements were computed from the variable gradient pulse sequence (TFE, 298 K, Δt = 100 ms, δ = 1 ms, 19 points, ledbpgp2s) using the dosy2d processing program in Topspin 4.0.9. These spectra were referenced to a coaxial insert tube containing DMSO-d_6.

O. References

1. M. Fujita, J. Yazaki, and K. Ogura, *J. Am. Chem. Soc.* 1990, 112, 14, 5645–5647, doi: 10.1021/ja00170a042.

2. M. J. Frisch, G. W. Trucks, H. B. Schlegel, G. E. Scuseria, M. A. Robb, J. R. Cheeseman, G. Scalmani, V. Barone, G. A. Petersson, H. Nakatsuji, X. Li, M. Caricato, A. V. Marenich, J. Bloino, B. G. Janesko, R. Gomperts, B. Mennucci, H. P. Hratchian, J. V. Ortiz, A. F. Izmaylov, J. L. Sonnenberg, D. Williams-Young, F. Ding, F. Lipparini, F. Egidi, J. Goings, B. Peng, A. Petrone, T. Henderson, D. Ranasinghe, V. G. Zakrzewski, J. Gao, N Rega, G. Zheng, W. Liang, M. Hada, M. Ehara, K. Toyota, R. Fukuda, J. Hasegawa, M. Ishida, T. Nakajima, Y. Honda, O. Kitao, H. Nakai, T. Vreven, K. Throssell, J. A. Montgomery, Jr., J. E. Peralta, F. Ogliaro, M. J. Bearpark, J. J. Heyd, E. N. Brothers, K. N. Kudin, V. N. Staroverov, T. A. Keith, R. Kobayashi, J. Normand, K. Raghavachari, A. P. Rendell, J. C. Burant, S. S. Iyengar, J. Tomasi, M. Cossi, J. M. Millam, M. Klene, C. Adamo, R. Cammi, J. W. Ochterski, R. L. Martin, K. Morokuma, O. Farkas, J. B. Foresman, and D. J. Fox, Gaussian 16 Revision C.01, Gaussian Inc., Wallingford CT, 2016.

3. E. F. Pettersen, T. D. Goddard, C. C. Huang, G. S. Couch, D. M. Greenblatt, E. C. Meng, and T.E. Ferrin. *J Comput. Chem*. 2004, **25**(13), 605–612, doi: 10.1002/jcc.20084.

4. D. A. Poole, III., E.O. Bobylev, S. Mathew, and J. N. H. Reek, J. N. H. *Chem. Sci.* 2020, **11**, 12350–12357, doi: 10.1039/D0SC03992F.

5. C. Bannwarth, S. Ehlert, and S. Grimme, J. Chem. Theory Comput. 2019, **15**(3), 1652–1671, DOI: 10.1021/acs.jctc.8b01176.

6. R. M. Betz and R. C. Walker, *J. Comput. Chem.* 2014, **36**(2), 79–87, DOI:10.1002/jcc.23775.

7. A.D. Bain *Prog. Nucl. Magn. Reson. Spectr.* **2003**, 43(3-4), 63–103, doi: 10.1016/j.pnmrs.2003.08.001
